# High‐Alkaline Water‐Splitting Activity of Mesoporous 3D Heterostructures: An Amorphous‐Shell@Crystalline‐Core Nano‐Assembly of Co‐Ni‐Phosphate Ultrathin‐Nanosheets and V‐ Doped Cobalt‐Nitride Nanowires

**DOI:** 10.1002/advs.202201311

**Published:** 2022-06-06

**Authors:** Thangjam Ibomcha Singh, Ashakiran Maibam, Dun Chan Cha, Sunghoon Yoo, Ravichandar Babarao, Sang Uck Lee, Seunghyun Lee

**Affiliations:** ^1^ Department of Chemical and Molecular Engineering Hanyang University ERICA Ansan 15588 Republic of Korea; ^2^ Center for Bionano Intelligence Education and Research Hanyang University ERICA Ansan 15588 Republic of Korea; ^3^ School of Science RMIT University Melbourne Victoria 3001 Australia; ^4^ Physical and Materials Division CSIR‐National Chemical Laboratory Pune 411 008 India; ^5^ Academy of Scientific and Innovative Research CSIR‐Human Resource Development Centre (CSIR‐HRDC) Campus Postal Staff College Area Ghaziabad Uttar Pradesh 201002 India; ^6^ Department of Applied Chemistry Hanyang University ERICA Ansan 15588 Republic of Korea; ^7^ Manufacturing CSIRO Normanby Road Victoria Clayton 3168 Australia

**Keywords:** core‐shell, hydrogen productions, metal nitrides, metal phosphates, synergistic effect, water‐splitting

## Abstract

Introducing amorphous and ultrathin nanosheets of transition bimetal phosphate arrays that are highly active in the oxygen evolution reaction (OER) as shells over an electronically modulated crystalline core with low hydrogen absorption energy for an excellent hydrogen evolution reaction (HER) can boost the sluggish kinetics of the OER and HER in alkaline electrolytes. Therefore, in this study, ultrathin and amorphous cobalt‐nickel‐phosphate (CoNiPO_x_) nanosheet arrays are deposited over vanadium (V)‐doped cobalt‐nitride (V_3%_‐Co_4_N) crystalline core nanowires to obtain amorphous‐shell@crystalline‐core mesoporous 3D‐heterostructures (CoNiPO_x_@V‐Co_4_N/NF) as bifunctional electrocatalysts. The optimized electrocatalyst shows extremely low HER and OER overpotentials of 53 and 270 mV at 10 mA cm^−2^, respectively. The CoNiPO_x_@V_3%_‐Co_4_N/NF (+/−) electrolyzer utilizing the electrocatalyst as both anode and cathode demonstrates remarkable overall water‐splitting activity, requiring a cell potential of only 1.52 V at 10 mA cm^−2^, 30 mV lower than that of the RuO_2_/NF (+)/20%‐Pt/C/NF (−) electrolyzer. Such impressive bifunctional activities can be attributed to abundant active sites, adjusted electronic structure, lower charge‐transfer resistance, enhanced electrochemically active surface area (ECSA), and surface‐ and volume‐confined electrocatalysis resulting from the synergistic effects of the crystalline V_3%_‐Co_4_N core and amorphous CoNiPO_x_ shells boosting water splitting in alkaline media.

## Graphical abstract

Benefiting from the synergistic effect of the amorphous‐shell and the crystalline‐core, cobalt‐nickel‐phosphate@vanadium‐doped cobalt‐nitride (CoNiPO_x_@V‐Co_4_N) amorphous‐shell@crystalline‐core mesoporous three‐dimensional heterostructure promotes water adsorption, dissociation, and optimal adsorption of the reaction intermediates resulting to excellent oxygen evolution reaction and hydrogen evolution reaction bifunctional activity in alkaline media.

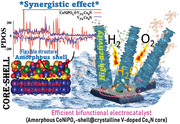

## 1 Introduction

Rising concern over global warming and the depletion of fossil reserves has accelerated the quest for alternative carbon‐free and sustainable fuels, leading to the rapid development of the H_2_‐based economy.^[^ ^1^ ^]^ Consequently, green hydrogen, produced from electrochemical water splitting powered by electricity generated from intermittent energy sources, has immense potential for sustainable energy owing to its high efficiency, large gravimetric energy density (≈140 MJ kg^−1^), and carbon‐free nature.^[^ ^2^ ^]^ However, the four proton‐coupled electron transfer (PCET) process at the anode and additional water dissociation step before proton reduction at the cathode greatly hinder the kinetics of the oxygen evolution reaction (OER) and hydrogen evolution reaction (HER), which require large overpotentials in alkaline media.^[^ ^3^, ^4^ ^]^ Therefore, the innovative design of highly active OER and HER bifunctional electrocatalysts is crucial for efficient water splitting in alkaline media by boosting their intrinsic and extrinsic catalytic properties.^[^ ^4^, ^5^, ^6^, ^7^, ^8^, ^9^ ^]^ Currently, Pt and its alloys show the best HER activities, whereas RuO_2_ and IrO_2_ are considered the best for OER; however, their large‐scale utilization is not economically sustainable because of their high cost and scarcity, thereby urgently demanding more economically viable earth‐abundant and low‐cost bifunctional electrocatalysts. Consequently, earth‐abundant first‐row transition metals such as Co‐ and Ni‐based metal phosphates (TMPOs) and metal nitrides (TMNs) with high OER and HER activities have recently been acknowledged as low‐cost and high‐efficiency electrocatalysts.^[^ ^10^, ^11^ ^]^ Binary TMNs, such as cobalt nitride of higher metallic cobalt (Co_4_N), showed metal‐like properties due to metallic Co—Co and Co—N bonds, thereby demonstrating excellent OER activities,^[^ ^12^ ^]^ while tuning the d‐band centers of metal nitrides via doping of secondary heteroatoms, such as V and Mo, can cause the downshifting of the d‐band away from the Fermi level owing to the filling up of the antibonding states, thereby facilitating the desorption of H_2_ and resulting in higher HER activities.^[^ ^13^ ^]^ TMPOs, on the other hand, also offer open frameworks in the layered structure along with the enriched redox behavior of the metal species and higher protonic conductivity resulting from the phosphate groups.^[^ ^14^ ^]^ Moreover, TMPOs in situ generate their corresponding metal (oxy)hydroxides during the OER reactions, which further boost the M‐OOH interactions for higher OER activities.^[^ ^14^ ^]^ However, both TMNs and TMPOs in their bulk state showed mediocre OER and HER performances because of the large dead volume, poor ionic conductivity, fewer active sites, and lower electrochemically active surface area (ECSA).

In this regard, it is necessary to tune the electrocatalytic properties both intrinsically and extrinsically via a combination of heteroatom doping and judicious interfacial engineering for generating abundant active sites, large specific surface area (SSA), and higher ECSA.^[^ ^15^, ^16^, ^17^, ^18^, ^19^ ^]^ Accordingly, core‐shell assembly of selected elemental compositions and electronically modulated different electroactive components can shorten ion diffusion paths, enhance abundant active sites, and boost the overall electrocatalytic activities owing to the synergistic effect resulting from the core and shell materials for OER and HER, respectively.^[^ ^20^ ^]^ Nanostructured TMNs such as 1D nanowires and TMPOs such as 2D nanosheets possess superior electrocatalytic performances owing to their higher SSA and large active exposed sites that facilitate ion transport and shorten the electrolyte diffusion paths.^[^ ^11^, ^21^, ^22^, ^23^ ^]^ Therefore, the fabrication of core‐shell heterostructures based on TMNs and TMPOs can result in superior OER and HER activities; however, the judicious choice of the electroactive components and their tandem heterostructures has not yet been explored to the best of our knowledge.

In addition, the electrocatalytic activities of such core‐shell heterostructures can be further improved by developing mesoporous and amorphous phases in tandem heterostructures. So far, developed core‐shell materials are mostly based on crystalline materials for both the core and shell.^[^ ^20^ ^]^ Crystalline materials show a lower metal dissolution rate and higher structural stability during long‐term electrochemical evaluation; however, owing to surface‐confined electrocatalysis, their utilization as shells may greatly hinder the overall electrocatalytic activities of the core‐shell heterostructures.^[^ ^24^, ^25^ ^]^ Despite controlling the thickness of the crystalline shells, the overall electrocatalytic performance of core‐shell heterostructures still suffers because of lower participation of the active sites from the internal core material caused by the structural rigidity of the crystalline shell, which hinders the easy diffusion of electrolyte ions into the core and results only in surface‐confined electrocatalysis.^[^ ^24^, ^25^ ^]^ In such cases, the utilization of amorphous materials as shells can offer easy electrolyte diffusion into the core owing to their structural flexibility, resulting in both surface‐ and volume‐confined electrocatalysis.^[^ ^24^, ^25^ ^]^ Moreover, unlike crystalline materials, amorphous materials possess coordination defects and high resistance to surface corrosion during long‐term operation because of their self‐healing properties.^[^ ^25^, ^26^ ^]^ For example, amorphous Ni‐doped Co phosphates,^[^ ^27^ ^]^ amorphous NiFe alloys,^[^ ^26^ ^]^ and amorphous/crystalline CoV‐Fe_0.28_ nanosheets,^[^ ^28^ ^]^ showed excellent OER activity. Thus, a highly OER‐active material of amorphous phase as the shell grown directly onto a highly HER active crystalline material as the core can harness the structural advantages of both amorphous and crystalline materials, which opens a new avenue for designing high‐performance core‐shell heterostructures as bifunctional electrocatalysts for efficient water splitting in alkaline media.

Considering the above limitations and the motivations, in this study, amorphous‐shell @crystalline‐core heterostructures consisting of ultrathin nanosheets of CoNiPO_x_ amorphous shell and a V‐doped Co_4_N nanowire crystalline core directly grown over a conductive 3D Ni foam substrate were designed to obtain a 3D mesoporous and binder‐free bifunctional electrocatalyst (CoNiPO_x_@V‐Co_4_N/NF) via a combined technique of nitridation and subsequent electrodeposition for OER, HER, and overall water splitting in alkaline media. The developed amorphous‐shell@crystalline‐core CoNiPO_x_@V‐Co_4_N/NF electrocatalysts exhibited impressive OER and HER electrocatalytic activity compared to NiPO_x_@V‐Co_4_N/NF, CoPO_x_@V‐Co_4_N/NF, V‐Co_4_N/NF, Co_4_N/NF, and their crystalline‐shell@crystalline‐core counterparts viz. (*C*)‐CoNiPO_x_@V‐Co_4_N/NF, (*C*)‐NiPO_x_@V‐Co_4_N/NF, (*C*)‐CoPO_x_@V‐Co_4_N/NF along with various other recently reported OER and HER electrocatalysts, indicating their superior bifunctional properties and robust stability. In addition, insight on the electronic modulation upon V‐doping and formation of amorphous‐shell@crystalline‐core heterostructures are also deciphered based on DFT‐based theoretical calculations for investigating the synergistic effects of the amorphous‐shell and the crystalline‐core and the associated mechanism for their electrocatalytic activities. This study presents a new approach for developing amorphous‐shell@crystalline‐core heterostructures to boost both intrinsic and extrinsic catalytic properties in the design of high‐performance OER and HER bifunctional electrocatalysts for overall water splitting in alkaline media.

## 2 Experimental Section

### 2.1 Chemical and Materials

Cobalt (II) nitrate hexahydrate (Co(NO_3_)_2_·6H_2_O, ACS reagent, ≥ 98.0%), Nickel nitrate hexahydrate (Ni(NO_3_)_2_.6H_2_O, ACS reagent, ≥ 98.0%), ammonium metavanadate (NH_4_VO_3_, ACS reagent, ≥ 98.0%), sodium hypophosphite (NaH_2_PO_2_, MW:87.98 g mol^−1^), urea (powder, Bioreagent), ammonium fluoride (NH_4_F, ACS reagent, ≥ 98.0%), and potassium hydroxide (KOH, ACS reagent, ≥ 85%, pellets), Pt on activated carbon (20% Pt loading), ruthenium (IV) oxide, and nafion 117 containing solution were obtained from Sigma‐Aldrich while Ni foam (porosity ≈98%, Ni percentage ≈ 99.9%, length ≈250 mm, thickness ≈ 1.5 mm, pore size ≈0.2–0.5 mm and density ≈ 380 g m^2^ ± 20,) was obtained from China (Taiyuan Liyuan Lithium Technology Co. Ltd.). All chemicals were used as received without any further purification.

### 2.2 Synthesis of Cobalt Carbonate Hydroxide Hydrate (Co‐CHH) Nanowires on Ni Foam (Co‐CHH/NF)

Co‐CHH/NF was prepared via a hydrothermal method, as reported previously, with slight modifications.^[^ ^29^ ^]^ Typically, cobalt nitrate hexahydrate (2 mmol), urea (4 mmol), and ammonium tetrafluoride (2 mmol) were dissolved in 50 mL DI water in a beaker under continuous magnetic stirring at 400 rpm for 30 min. Then, the solution was transferred to a Teflon‐lined stainless‐steel autoclave (100 mL), along with a pair of pre‐cleaned Ni foam (2 × 4 cm^−2^) pieces, and sealed. The stainless‐steel autoclave was then placed inside an electric oven and heated to a temperature of 120 °C for 6 h. Finally, the autoclave was allowed to cool to room temperature (25 °C) and the NF pieces were collected, washed with pure water and ethanol several times, and finally dried at 60 °C for 12 h. The precipitates obtained at the bottom of the Teflon cup were also collected by centrifugation and dried in an oven at the same temperature.

### 2.3 Synthesis of Vanadium‐Doped Co‐CHH/NF (V_x_‐Co‐CHH/NF, x = 0, 1, 3, and 5 at. % Respectively)

V_x_‐Co‐CHH/NF was also prepared under experimental conditions similar to those of Co‐CHH/NF by adding different quantities of ammonium metavanadate as the V source (1, 3, and 5 atomic %) to Co‐CHH/NF precursor solutions such that the total concentration of the metal salts remained the same (2 mmol). Finally, the samples were collected, washed with pure water and ethanol, and dried at 60 °C for 12 h.

### 2.4 Synthesis of Vanadium Doped Cobalt‐Nitride Nanowires on Ni Foam (V_x_‐Co_4_N/NF, x = 0, 1, 3, 5 at. %)

V_x_‐Co_4_N/NF was prepared via thermal nitridation of the as‐prepared V_x_‐Co‐CHH/NF samples using ammonia gas as the N source, similar to that previously reported with slight modifications.^[^ ^12^ ^]^ Typically, a piece of V_x_‐Co‐CHH/NF was initially placed on a quartz boat inside the chemical vapor deposition (CVD) and vacuumed to low pressure (approximately 10^−3^ Torr). Then, 500 sccm of NH_3_ gas was passed into the chamber and the temperature was raised at a slow heating rate of 2.5 °C min^−1^ to 450 °C and then maintained for 2 h. Finally, the heating was stopped, and the sample was cooled to room temperature (25 °C). Compared to all other concentrations, V_3%_‐Co_4_N/NF (3 at. %) was found to be the best sample. The mass loadings of Co_4_N and V_3%_‐Co_4_N electrocatalysts over 1 cm^−2^ area of Ni foam were found to be ≈ 3.46 and 3.58 mg respectively.

### 2.5 Synthesis of Amorphous‐Shell@Crystalline‐Core CoNiPO_x_@V_3%_‐Co_4_N/NF Heterostructures

CoNiPO_x_@V_3%_‐Co_4_N/NF heterostructures were prepared by electrodepositing a CoNiPO_x_ nanosheet shell on a pre‐synthesized V_3%_‐Co_4_N/NF core using cyclic voltammetry (CV), similar to that previously reported.^[^ ^30^ ^]^ Typically, the electrodeposition process was carried out using V_3%_‐Co_4_N/NF (2 × 2 cm^−2^) as the working electrode, Ag/AgCl (vs sat. KCl) as the reference electrode, and Pt wire as the counter electrode. The solution for electrodeposition of the CoNiPO_x_ shell was prepared by dissolving 1 mmol each of Co(NO_3_)_2_. 6H_2_O, Ni(NO_3_)_2_.6H_2_O, and NaH_2_PO_2_ (MW:87.98 g mol^−1^) as Co, Ni, and P sources in a 100 mL solution mixture of ethanol and DI water in a volume ratio of 1:1. CV was employed for the electrodeposition at different scan rates of 3, 5, 6, and 7 mV s^−1^ for two segments in the potential range of −1.2 to 0.2 V (vs Ag/AgCl) to determine the optimum condition. Among the various conditions, the growth of CoNiPO_x_ nanosheets was observed to be the most uniform, well‐developed, and ultrathin at a scan rate of 6 mV s^−1^ and was chosen as the optimum condition for the electrodeposition of CoNiPOx nanosheets throughout the experiment. For comparison, binary CoPO_x_ and NiPO_x_ nanosheets were also electrodeposited under the same optimum conditions (6 mV s^−1^ for two segments) using their corresponding electrodeposition solutions. Finally, the electrodeposited samples were washed in DI water several times and dried in an oven for 12 h at 60 °C. The mass loadings of the CoNiPO_x_@V_3%_‐Co_4_N, CoPO_x_@V_3%_‐Co_4_N, and NiPO_x_@V_3%_‐Co_4_N electrocatalysts over 1 cm^−2^ area of Ni foam were found to be ≈6.3, 5.23, and 4.67 mg respectively.

### 2.6 Synthesis of Crystalline‐Shell@Crystalline‐Core (*C*)‐CoNiPO_x_@V_3%_‐Co_4_N/NF, (*C*)‐NiPO_x_@V_3%_‐Co_4_N/NF, and (*C*)‐CoPO_x_@V_3%_‐Co_4_N/NF Heterostructures

For the preparation of crystalline‐shell@crystalline‐core heterostructures, the as‐prepared electrodeposited CoNiPO_x_@V_3%_‐Co_4_N/NF, NiPO_x_@V_3%_‐Co_4_N/NF, and CoPO_x_@V_3%_‐Co_4_N/NF amorphous‐shell@crystalline‐core heterostructures were annealed at 400 °C under argon (Ar) atmosphere for a duration of 2 h. The heating rate and the flow of Ar gas were maintained at 2 °C per min and 500 sccm throughout the annealing process. Finally, the annealed samples were cool down to room temperature. These samples were denoted as (*C*)‐CoNiPO_x_@V_3%_‐Co_4_N/NF, (*C*)‐NiPO_x_@V_3%_‐Co_4_N/NF, and (*C*)‐CoPO_x_@V_3%_‐Co_4_N/NF to represent the crystalline‐shell@crystalline‐core heterostructures.

For comparison of the electrocatalytic performance, RuO_2_/NF and Pt/C/NF (20% Pt loading) electrocatalysts were prepared using a catalyst ink coating method, similar to those previously reported.^[^ ^9^, ^31^ ^]^ For this, 5 mg each of commercial RuO_2_ and 20% Pt‐loaded graphitic carbon were separately dispersed in a solution mixture of 750 µL of isopropanol, 200 µL of DI water, and 50 µL of Nafion solution by ultrasonication for 2 h. The prepared catalyst ink was then coated on two pieces of Ni foam (1×1 cm^−2^ area). The coated Ni foam pieces were vacuum dried in a vacuum oven at 60 °C for 24 h.

### 2.7 Electrochemical Measurements

#### 2.7.1 Evaluations in Three Electrodes and Two Electrode Configurations

Linear sweep voltammetry (LSV), Cyclic voltammetry (CV), and electrochemical impedance spectroscopy (EIS) were employed in a three‐electrode configuration using a ZIVE SP1, WonAtech electrochemical workstation. All EIS measurements were performed in the frequency range of 1^−2^ –10^5 ^Hz with a voltage amplitude of 5 mV. Freshly prepared 1 m KOH (pH = 14), 1 cm^2^ area of the prepared materials, graphite rod, and Hg/HgO were used as the electrolyte, working electrode, counter electrode, and reference electrode, respectively, during the electrochemical evaluation of the OER and HER. To check the effect of O_2_/H_2_ gas saturating the 1 m KOH electrolyte on OER/HER activities, 1 m KOH electrolytes saturated with O_2_ and H_2_ gas were also used for OER and HER LSV measurements.

All the experimentally measured potentials for each half‐cell reaction were converted to the reversible hydrogen electrode (RHE) scale using Equation (1):^[^ ^21^ ^]^

(1) $$E\left(V\mathrm{vs}R\mathrm{HE}\right)=E\left(V\mathrm{vs}\mathrm{Hg}/\mathrm{HgO}\right)+0.059\times \mathrm{pH}+E{}_{\mathrm{Hg}/\mathrm{HgO}}^{\circ }$$

where *E* (*V* vs Hg/HgO) is the experimentally measured potential with respect to the Hg/HgO reference electrode and *E°*_Hg/HgO_ is the electrode potential of the Hg/HgO reference electrode in 1 m KOH (pH = 14) electrolyte and is equal to 0.098 V.

The iR‐correction of the measured potentials from the LSV profiles for both the OER and HER was performed using Equation (2):^[^ ^21^ ^]^

(2) $$E_{\mathrm{iR}}=E\left(V\mathrm{vs}\mathrm{RHE}\right)-iR_{s}$$

where *i* is the current and *R_s_* is the series resistance obtained from the Nyquist plots.

The Tafel plot was obtained according to Equation (3),

(3) $$\eta =b*\log\left[i\right]+a$$

where *η* is the overpotential (V), and *b* is the Tafel slope (mV dec^−1^).^[^ ^21^ ^]^

The electrochemically active surface area (ECSA) of the electrocatalysts was calculated using Equation (4),^[^ ^32^ ^]^

(4) $$\mathrm{ECSA}=\frac{C_{\mathrm{dl}}}{C_{s}}$$

where *C*_dl_ is the electric double‐layer capacitance calculated from the non‐Faradaic region, and *C*_s_ is the specific capacitance of a flat, smooth electrode surface, whose value was numerically taken as 40 *μ*F cm^−2^.^[^ ^9^ ^]^ For the calculation of *C*_dl_, cyclic voltammograms of the electrode material were recorded in the non‐Faradaic region at various scan rates, and a graph of scan rates versus current densities (Δ*j = j*_anodic_ − *j*_cathodic_) was plotted to determine the slope of the graph through a linear fitting. The value of the slope was numerically equal to twice the *C*_dl_ value; therefore, *C*_dl_ was half the slope value.

### 2.8 Material Characterizations

Powder X‐ray diffraction (P‐XRD, Miniflex 600, Rigaku Corporation, Japan) with Cu K*α* radiation (wavelength = 0.15406 nm) in the 2*θ* range of 10–80*°* at a scan rate of 3*°* min^−1^ was used to investigate the crystallinity and phase of the prepared materials. Field‐emission scanning electron microscopy (FE‐SEM) images and energy‐dispersive X‐ray spectroscopy (EDS) were obtained using a Hitachi S‐4300 FE‐SEM equipped with a Horiba EMAXx‐stream2 EDS system at Hanyang University ERICA campus, Ansan, South Korea, and field‐emission transmission electron microscopy (FE‐TEM) (JEM‐F200, JEOL, Ltd., Japan, 200 kV) at Seoul National University, Seoul, South Korea were employed to investigate the surface morphology, internal structure, and elemental composition. Further, X‐ray photoelectron spectroscopy (XPS) (Theta Probe; Thermo Fisher Scientific, UK) was used to analyze the surface chemical compositions and valence states of the elements. The SSA and pore size distributions were determined using the Brunauer–Emmett–Teller (BET) and Barrett–Joyner–Halenda (BJH) methods based on multipoint nitrogen adsorption‐desorption experiments at 77 K using a BELSORP‐mini II (BEL Inc., Japan) analyzer after degassing the samples at 100 *°*C for 15 h under dynamic vacuum.

## 3 Result and Discussion

**Figure 1:**
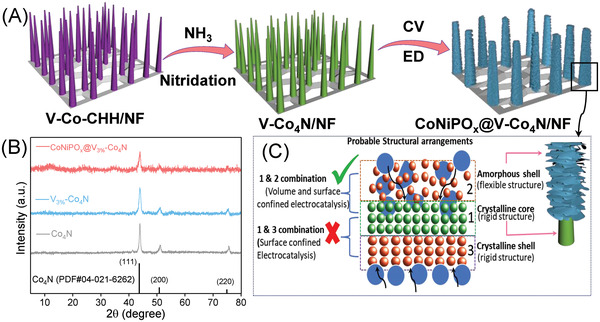
A) Schematic diagram for the synthesis of amorphous‐shell@crystalline‐core CoNiPO_x_@V‐Co_4_N 3D heterostructures on conductive and porous Ni foam substrate, B) P‐XRD patterns of the crystalline Co_4_N, V_3%_‐Co_4_N nanowires, and CoNiPO_x_@V_3%_‐Co_4_N amorphous‐shell@crystalline‐core 3D heterostructures, and C) schematic representation of the plausible structural arrangements of the crystalline (1, 3) and amorphous (2) materials, resulting in surface‐ and volume‐confined electrocatalysis.

The amorphous‐shell@crystalline‐core of the CoNiPO_x_@V‐Co_4_N/NF 3D heterostructure was developed using combined hydrothermal, thermal nitridation, and electrodeposition techniques, as shown schematically in **Figure** **1**. Initially, using a simple hydrothermal method, different concentrations of V‐doped Co‐CHH nanowires were prepared on a Ni foam substrate (V_x_‐Co‐CHH/NF, x = 0, 1, 3, and 5 at .%), which was then converted into their corresponding metal nitrides (V_x_‐doped Co_4_N/NF) via thermal nitridation using NH_3_ gas followed by the electrodeposition of amorphous CoNiPO_x_ nanosheets using the CV technique (Figure 1A).

P‐XRD was used to determine the phases of the prepared materials in their powder forms without the Ni foam substrates, as shown in Figure 1B and Figures S1 and S2, Supporting Information. The P‐XRD pattern of the as‐prepared Co‐CHH closely resembles that of Co(CO_3_)_0.5_OH 0.11H_2_O (JCPDS card no. 48–0083)^[^ ^29^ ^]^ and Co_6_(CO_3_)_2_(OH)_8_.H_2_O, which is isostructural to Co(Co_3_)_0.35_Cl_0.20_(OH)_1.10_.1.74H_2_O (JCPDS card no. 038–0547) (Figure S1A,B, Supporting Information).^[^ ^33^ ^]^ However, a careful analysis, through a thorough comparison of the stick patterns of the two JCPDS card numbers with our P‐XRD pattern, revealed a stronger correlation of the resulting P‐XRD patterns with JCPDS card no. 038–0547 compared with 48–0083 (Figure S1B, Supporting Information), indicating that the prepared Co‐CHH exhibits the Co_6_(CO_3_)_2_(OH)_8_.H_2_O phase (Figure S1B, Supporting Information). This result is also in agreement with the previous investigation, which concluded that the phase of the hydrothermally prepared Co‐CHH should be Co_6_(CO_3_)_2_(OH)_8_.H_2_O (JCPDS card no. 038–0547) and not Co(CO_3_)_0.5_OH·0.11H_2_O (JCPDS card no. 48–0083).^[^ ^33^ ^]^ Even after doping with V at different concentrations, the corresponding P‐XRD patterns of the V‐doped Co‐CHH exhibited peaks identical to those of Co‐CHH without the evolution of any other new peaks, indicating the absence of any other impurity phases (Figure S1A, Supporting Information). After nitridation of V_x_‐Co‐CHH (x = 0, 1, 3, and 5 at. %), at 450 °C under NH_3_, the resulting materials showed identical P‐XRD peaks that matched well with the (111), (200), and (220) planes of Co_4_N (ICDD PDF no. 04‐021‐6262), confirming their successful conversion to nitrides (Figure S2A,B, Supporting Information).^[^ ^13^ ^]^ Although the P‐XRD pattern of Co_4_N nanowires (Figure S2A, Supporting Information) appeared to be similar to those of their metallic cobalt phases viz. metallic Co (PDF#00‐001‐1255), and Co_4_ (PDF#96‐901‐1624), etc., a careful analysis of the peak positions showed that the obtained P‐XRD pattern of the Co_4_N nanowires was more closely matched to those of Co_4_N (PDF#04‐021‐6262), thus, confirming the formation of Co_4_N phase and not the metallic Co phases.

Preliminary electrochemical investigations further revealed that V‐doping of 3 at. % showed better OER and HER performance compared with other concentrations of V‐doping (Figure S3A,B, Supporting Information) owing to the lowest charge‐transfer resistance (*R*_ct_) (Figure S3C, Supporting Information) and higher ECSA (Figure S3D–H, Supporting Information). Thus, V_3%_‐Co_4_N/NF was used as the optimized core material for the synthesis of core‐shell materials throughout the following synthesis steps. However, after electrodeposition, the P‐XRD patterns of the resulting CoNiPO_x_@V_3%_‐Co_4_N/NF, CoPO_x_@V_3%_‐Co_4_N/NF, and NiPO_x_@V_3%_‐Co_4_N/NF heterostructures (Figure 1B and Figure S2C, Supporting Information) showed peaks corresponding to only the core V_3%_‐Co_4_N material, and no extra distinct peaks corresponding to the electrodeposited CoNiPO_x_, NiPO_x_, and CoPO_x_ shells were observed, indicating their amorphous nature. The amorphous nature of the electrodeposited materials has also been observed in many previous reports.^[^ ^34^, ^35^, ^36^ ^]^ Thus, P‐XRD analysis indicated the formation of amorphous‐shell@crystalline‐core heterostructures for the CoNiPO_x_@V_3%_‐Co_4_N/NF, NiPO_x_@V_3%_‐Co_4_N/NF, and CoPO_x_@V_3%_‐Co_4_N/NF electrocatalysts. Interestingly, the presence of such amorphous materials as shells is expected to enhance the electrocatalytic properties of the developed core‐shell heterostructures because of the existence of both surface‐ and volume‐confined electrocatalysis superior to those of the crystalline shells.^[^ ^25^ ^]^

**Figure 2:**
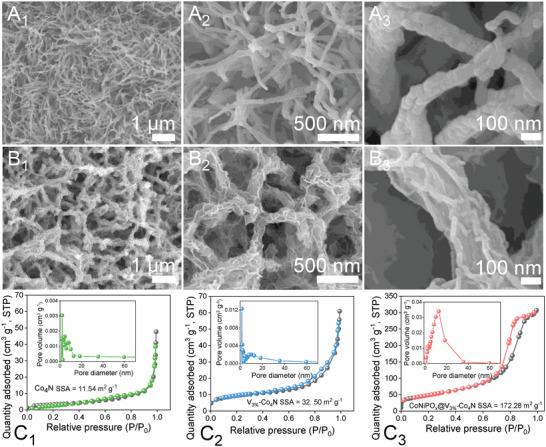
Low‐ and high‐magnification FE‐SEM images of A_1_–A_3_) V_3%_‐Co_4_N and B_1_–B_3_) CoNiPO_x_@V_3%_‐Co_4_N; BET SSA and BJH pore‐size distribution of C_1_) Co_4_N, C_2_) V_3%_‐Co_4_N, and C_3_) CoNiPO_x_@V_3%_‐Co_4_N, respectively.

Further, the surface morphology of the prepared electrocatalysts was investigated at every synthesis step using the FE‐SEM technique (**Figure** **2** and Figures S4–S7, Supporting Information). The FE‐SEM images of the pristine Co‐CHH/NF (Figure S4, Supporting Information) showed uniform and thin nanowire structures, and upon increasing the V doping from 1 to 5 at. %, the resulting V_x_‐Co‐CHH demonstrated almost identical nanowire structures, with a slight decrease in the size of the nanowires (Figure S5, Supporting Information). However, the overall growth of the nanowires on the NF substrate became highly agglomerated, forming microspheres at higher V doping (Figure S5A_3_, Supporting Information), which indicated that 3 at. % is the optimum concentration owing to more uniform and well‐interspaced nanowires compared to those of 1 at. % and 5 at. % V‐doped nanowires (Figure S5B_1_,B_3_, Supporting Information). After nitridation under NH_3_ gas, the FE‐SEM images of V_3%_‐Co_4_N/NF showed enormous surface defects and roughness compared with pristine Co‐CHH/NF‐derived Co_4_N/NF (Figure 2A and Figure S6, Supporting Information). Furthermore, FE‐SEM images of the electrodeposited CoNiPO_x_@V_3%_‐Co_4_N/NF prepared under different electrodeposition conditions (various scan rates of 3, 5, 6, and 7 mV s^−1^ for two segments, as shown in Figure S7, Supporting Information, and Figure 2B_1_–B_3_) demonstrate the growth of the CoNiPO_x_ nanosheet shells during electrodeposition. At a slow scan rate of 3 mV s^−1^, apart from the extremely thick and bulky agglomerations, no well‐defined nanosheet structures were formed (Figure S7A_1_–A_3_, Supporting Information). As the scan rates increased to 5, 6, and 7 mV s^−1^, well‐developed CoNiPO_x_ nanosheets were observed to grow on the core materials more uniformly but at varying thicknesses (Figure 2B_1_–B_3_ and Figure S7, Supporting Information). At a scan rate of 5 mV s^−1^, the growth of CoNiPO_x_ nanosheets was too low and not uniformly developed (Figure S7B_1_–B_3_, Supporting Information), whereas the nanosheets were much larger and thicker at a scan rate of 7 mV s^−1^ (Figure S7C_1_–C_3_, Supporting Information). In comparison, the FE‐SEM images of the CoNiPO_x_ nanosheets prepared at a scan rate of 6 mV s^−1^ showed the most uniform growth over the core material with a desirable thickness, as can be seen in Figure 2B_1_–B_3_. Such uniform growth of the ultrathin nanosheets is expected to enhance the electrode‐electrolyte interactions, facilitate the ion diffusion process, and enhance the ECSA, thereby improving the electrocatalytic activities.^[^ ^37^, ^38^, ^39^ ^]^ Interestingly, preliminary electrochemical evaluations also showed that CoNiPO_x_@V_3%_‐Co_4_N/NF prepared at a scan rate of 6 mV s^−1^ showed the best OER and HER activity compared to those prepared at 5 and 7 mV s^−1^ (Figure S8, Supporting Information), which could be attributed to its higher ECSA resulting from more uniform and ultrathin nanosheets (Figure S8C–F, Supporting Information). The electrodeposition process of CoNiPO_x_ nanosheets can be represented by the following reaction steps:^[^ ^30^, ^40^, ^41^ ^]^

(5) $$\begin{array}{c} \mathrm{Na}H_{2}PO_{2}+H_{2}O\overset{\left(\mathrm{electro}-\mathrm{oxidation}\right)}{\to }H_{2}P{O_{3}}^{-}+Na^{+}+2H^{+}+2e^{-} \end{array}$$

(6) $$H_{2}P{O_{3}}^{-}⇋\mathrm{HP}{O_{3}}^{2-}+H^{+}$$

(7) $$\mathrm{HP}{O_{3}}^{2-}+H_{2}O\overset{\left(\mathrm{electro}-\mathrm{oxidation}\right)}{\to }\mathrm{HP}{O_{4}}^{2-}+2H^{+}+2e^{-}$$

(8) $$Ni^{2+}+Co^{2+}+2\mathrm{HP}{O_{4}}^{2-}\to \mathrm{NiCo}{\left(\mathrm{HP}O_{4}\right)}_{2}$$

Net reaction can be represented as:

(9) $$\begin{array}{ccc} & & 2\mathrm{Na}H_{2}PO_{2}+Ni^{2+}+Co^{2+}+4H_{2}O\to \mathrm{NiCo}{\left(\mathrm{HP}O_{4}\right)}_{2}\\ & & \;+\;10H^{+}+2Na^{+}+8e^{-} \end{array}$$

For comparison, CoPO_x_ and NiPO_x_ nanosheets were also synthesized over the V_3%_‐Co_4_N/NF core under the same electrodeposition conditions (6 mV s^−1^ for two segments) to develop CoPO_x_@V_3%_‐Co_4_N/NF and NiPO_x_@V_3%_‐Co_4_N/NF heterostructures, and their corresponding FE‐SEM images are shown in Figure S9A,B, Supporting Information. Compared to the larger and thicker nanosheets of CoPO_x_, the electrodeposited NiPO_x_ nanosheets showed much smaller and thinner nanosheets; however, both samples were less uniform and slightly agglomerated compared to CoNiPO_x_ nanosheets, as observed in Figure 2B_1_–B_3_. Thus, FE‐SEM analysis showed that CoNiPO_x_@V_3%_‐Co_4_N/NF heterostructures showed the optimum morphology in terms of high uniformity with ultrathin nanosheets compared with NiPO_x_@V_3%_‐Co_4_N/NF and CoPO_x_@V_3%_‐Co_4_N/NF. Nonetheless, the direct growth of ultrathin bimetallic metal phosphate nanosheets is expected to enhance the number of active sites, shorten the ion transport path, and improve the porosity and SSA. Accordingly, the BET SSA and BJH pore‐size distributions were evaluated from N_2_ sorption isotherm measurements, as shown in Figure 2C_1_–C_3_. The electrodeposited amorphous‐shell@crystalline‐core CoNiPO_x_@V_3%_‐Co_4_N heterostructure showed a typical Type‐IV isotherm curve with a higher SSA of 172.28 m^2^ g^−1^ (Figure 2C_3_) and mesoporous nature (inset Figure 2C_3_) compared with those of V_3%_‐Co_4_N (32.50 m^2^ g^−1^) (Figure 2C_2_) and Co_4_N (11.54 m^2^ g^−1^) (Figure 2C_1_), respectively, suggesting that the in situ growth of ultrathin and amorphous bimetallic CoNiPO_x_ nanosheets greatly improved the SSA and porosity of the resultant CoNiPO_x_@V_3%_‐Co_4_N heterostructure, which greatly facilitated electrocatalytic activities.^[^ ^9^ ^]^

**Figure 3:**
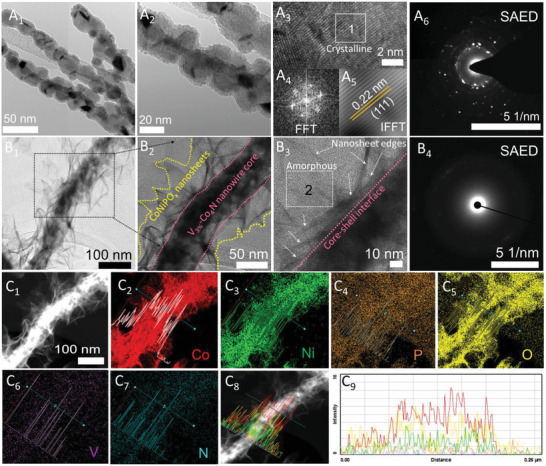
(A_1_,A_2_ and B_1_,B_2_) Low‐resolution TEM images, (A_3_ and B_3_) high‐resolution TEM images, (A_4_ and A_5_) FFT and IFFT of region 1 of A_3_, (A_6_ and B_4_) SAED of region 1 in A_3_ of V_3%_‐Co_4_N core and region 2 in B_3_ of CoNiPO_x_ nanosheets shell, (C_1_) HAADF‐STEM image of CoNiPO_x_@V_3%‐_Co_4_N heterostructures, (C_2_‐C_9_) elemental color mapping showing the presence of Co (C_2_), Ni (C_3_), P (C_4_), O (C_5_), V (C_6_), N (C_7_), and the corresponding overlapped line mappings (C_8_,C_9_) for CoNiPO_x_@V_3%_‐Co_4_N electrocatalysts.

Further, the internal structure of the prepared electrocatalysts, such as CoNiPO_x_@V_3%_‐Co_4_N/NF, V_3%_‐Co_4_N/NF, and pristine Co_4_N/NF electrocatalysts were examined using transmission electron microscopy (TEM), as shown in **Figure** **3** and Figures S10 and S11, Supporting Information. Compared with pristine Co_4_N nanowires (Figure S10A,B, Supporting Information), the TEM of V_3%_‐Co_4_N nanowires showed a higher degree of defect and roughness on the edges of the nanowires (Figure 3A_1_,A_2_ and Figure S11A_1_, Supporting Information). The Fast Fourier transform (FFT) and the corresponding inverse Fast Fourier transform (IFFT) analyses of region 1 of the high‐resolution TEM (HR‐TEM) image in Figure S10C, Supporting Information, of the Co_4_N nanowire indicated an interplanar distance of 0.267 nm that matched well with that of the (111) plane of Co_4_N (04‐021‐6262), a result that correlated with the P‐XRD analysis (Figure 1B). Furthermore, the high‐angle annular dark‐field scanning transmission electron micropscopy (HAADF‐STEM) image (Figure S10F_1_, Supporting Information) and elemental color mapping of the Co_4_N nanowire showed the presence of Co and N as its constituent elements and their spatial distribution (Figure S10F_2_,F_3_, Supporting Information). On the other hand, the FFT and the corresponding IFFT analysis of the HR‐TEM image of V_3%_‐Co_4_N at region 1 in Figure 3A_3_ showed a slightly decreased interplanar distance of 0.22 nm for (111) due to V doping, in agreement with previous reports.^[^ ^13^ ^]^ Furthermore, the low‐resolution TEM images (Figure 3B_1_,B_2_) and HAADF‐STEM images (Figure 3C_1_) of CoNiPO_x_@V_3%_‐Co_4_N/NF showed a clear core‐shell heterostructure consisting of numerous ultrathin CoNiPO_x_ nanosheets directly grown on the defect‐rich nanowire‐like structures of the V_3%_‐Co_4_N core, in agreement with the FE‐SEM findings (Figure 2B). The HR‐TEM image of the CoNiPO_x_ shell portion of CoNiPO_x_@V_3%_‐Co_4_N/NF shown in Figure 3B_3_ further demonstrates the ultrathin nature of the nanosheets with various edges (indicated by dotted lines) and the absence of clear lattice fringes, indicating its amorphous nature (inset Figure 3B_3_) in contrast to the distinct and clear lattice fringes of the V_3%_‐Co_4_N core material (Figure 3A_3_–A_5_). This result suggests a crystalline nature of the V_3%_‐Co_4_N core material (Figure 3A_3_–A_5_), which is in agreement with the P‐XRD results (Figure 1B). In addition, the selective area under the electron diffraction (SAED) pattern of the core V_3%_‐Co_4_N in Figure 3A_6_ shows clear bright dots, indicating its crystalline nature, whereas the SAED pattern recorded in region 2 of Figure 3B_3_ for the electrodeposited CoNiPO_x_ nanosheet shells (Figure 3B_4_) shows only circular white rings, indicating its amorphous nature. The amorphous nature of the electrodeposited shell and the crystalline nature of the core revealed by SAED analysis are also in agreement with the results of the P‐XRD analysis (Figure 1B), thereby confirming the formation of an amorphous‐shell@crystalline‐core heterostructure. The formation of amorphous shells over the crystalline core is expected to enhance the overall electrocatalytic activities of the resulting CoNiPO_x_@V_3%_‐Co_4_N heterostructures because of the existence of both surface‐ and volume‐confined electrocatalysis enabled by the amorphous shells, as observed in many amorphous materials reported previously.^[^ ^26^, ^27^, ^28^ ^]^ Furthermore, the elemental composition of the constituents and their spatial distributions in the CoNiPO_x_@V_3%_‐Co_4_N heterostructure and V_3%_‐Co_4_N were investigated using HAADF‐STEM elemental mapping, as shown in Figure 3C_2_–C_9_ and Figure S11A_2_, Supporting Information, respectively, confirming the presence of Co, Ni, P, O, V, and N as the constituent elements for CoNiPO_x_@V_3%_‐Co_4_N heterostructure (Figure 3B_2_) and V, Co, and N for the core V_3%_‐Co_4_N nanowires (Figure S11A_2_, Supporting Information). Notably, the intensity of O for the CoNiPO_x_@V_3%_‐Co_4_N heterostructure is much more intense than that of P (Figure 3C_4_ and C_5_), which indicates that the in situ electrodeposited CoNiPO_x_ nanosheets shell is composed of mostly metal‐phosphate and not metal‐phosphides.^[^ ^14^ ^]^ Thus, P‐XRD and TEM analyses confirmed the formation of the amorphous‐shell@crystalline‐core of CoNiPO_x_@V_3%_‐Co_4_N heterostructures and their constituent elements.

**Figure 4:**
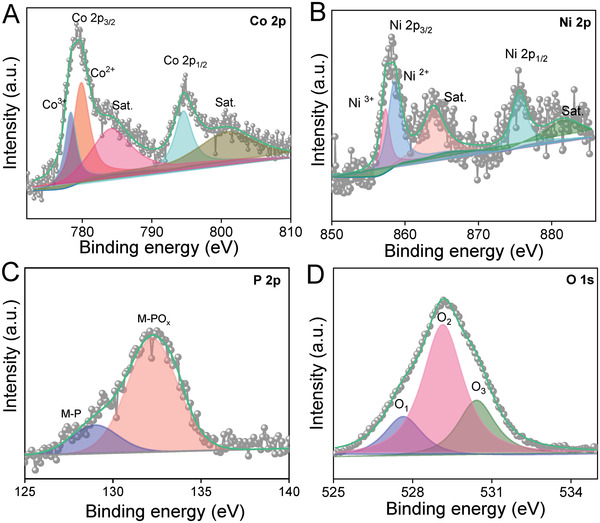
Deconvoluted high‐resolution XPS spectra of A) Co 2p, B) Ni 2p, C) P 2p, and D) O1s spectra of the electrodeposited CoNiPO_x_ shells of CoNiPO_x_@V_3%_‐Co_4_N/NF electrocatalysts.

The near‐surface chemical oxidation states of the prepared CoNiPO_x_@V_3%_‐Co_4_N/NF electrocatalysts were further examined using X‐ray photoelectron spectroscopy (XPS), as shown in **Figure** **4** and Figure S12, Supporting Information. The survey spectrum of CoNiPO_x_@V_3%_‐Co_4_N (Figure S12A, Supporting Information) showed the presence of Co, Ni, P, and O elements apart from the low‐intensity N and V peaks, indicating the presence of CoNiPO_x_ shells over the V_3%_‐Co_4_N core, which is consistent with the results of the HAADF‐STEM color mapping analysis (Figure 3B_2_). The high‐resolution Co 2p spectrum showed two dominant peaks at binding energies of ≈780 and ≈796 eV, corresponding to Co 2p_3/2_ and Co 2p_1/2_ peaks with satellite peaks at ≈ 784 and 801 eV, respectively^[^ ^13^, ^42^ ^]^ (Figure 4A). Furthermore, the Ni 2p XPS spectrum in Figure 4B showed peaks at a binding energy of ≈858 and ≈875 eV corresponding to Ni 2p_3/2_ and Ni 2p_1/2_, respectively, with their respective satellite peaks at ≈863 and ≈881 eV.^[^ ^43^ ^]^ In addition, the deconvoluted P 2p XPS spectrum also showed two peaks at a binding energy of ≈128.91 and 132.31 eV corresponding to the presence of metal‐phosphides (M‐P) and –phosphate (M‐PO_x_) species, respectively (Figure 4C). However, the peak intensity corresponding to M‐PO_x_ is much higher than that of M‐P, indicating that the electrodeposited material was mainly metal phosphates, which correlates with the HAADF‐STEM elemental color mapping and is similar to previous reports.^[^ ^30^, ^31^ ^]^ Deconvoluted O 1s spectrum also showed three peaks denoted as O_1_, O_2,_ and O_3_ at binding energies of 527.61, 529.14, and 530.42 eV corresponding to adsorbed water, surface oxygen, and P‐O species, respectively.^[^ ^31^, ^44^ ^]^

Furthermore, XPS analysis of the V_3%_‐Co_4_N core material was also performed by recording the survey spectrum and high‐resolution core XPS spectra of the constituent elements, as shown in Figure S12, Supporting Information. The Co 2p XPS spectrum of the V_3%_‐Co_4_N core material (Figure S12B, Supporting Information) showed deconvoluted peaks at 779.7, 781.2, and 782.9 eV corresponding to the Co—Co, Co—O, and Co—N moieties of Co_4_N.^[^ ^42^ ^]^ The V 2p XPS spectrum (Figure S12C, Supporting Information) showed a typical asymmetric spin‐orbit splitting, with a slightly broader peak corresponding to V 2p_1/2_ than that of V 2p_3/2_.^[^ ^45^ ^]^ Upon deconvolution, peaks corresponding to various oxidation states of V viz. V_(0)_, V_(II)_, V_(IV)_, and V_(V)_ were also observed, indicating the presence of both metallic and surface‐oxidized V species (Figure S12C, Supporting Information).^[^ ^45^ ^]^ Furthermore, the deconvoluted XPS spectra of N 1s showed two peaks at binding energies of ≈397.13 and ≈399.34 eV corresponding to the Co—N and N—H species, respectively (Figure S12D, Supporting Information).^[^ ^13^, ^42^ ^]^ Thus, XPS analysis confirmed the elemental compositions and surface chemical states of the developed CoNiPO_x_@V_3%_‐Co_4_N and V_3%_‐Co_4_N electrode materials.

For comparison, we also prepared the crystalline‐shell@crystalline‐core heterostructures denoted as (*C*)‐CoNiPO_x_@V_3%_‐Co_4_N/NF, (*C*)‐CoPO_x_@V_3%_‐Co_4_N/NF, and (*C*)‐NiPO_x_@V_3%_‐Co_4_N/NF by annealing the electrodeposited samples under Ar atmosphere at 400 °C for 2 h. The FE‐SEM images of (*C*)‐CoNiPO_x_@V_3%_‐Co_4_N/NF, (*C*)‐NiPO_x_@V_3%_‐Co_4_N/NF, and (*C*)‐CoPO_x_@V_3%_‐Co_4_N/NF are shown in Figure S13, Supporting Information. The FE‐SEM images showed that even after annealing, the morphology of the core‐shell heterostructures was all preserved (Figure S13A–C, Supporting Information), with a slight agglomeration of the nanosheet shells. Among them, the morphology of the (*C*)‐CoNiPO_x_@V_3%_‐Co_4_N/NF core‐shell heterostructures was found to be most uniform (Figure S13A, Supporting Information) compared to (*C*)‐CoPO_x_@V_3%_‐Co_4_N/NF and (*C*)‐NiPO_x_@V_3%_‐Co_4_N/NF (Figure S13B,C, Supporting Information). Further, the P‐XRD patterns of the annealed samples were also recorded to check their crystallinity, as shown in Figure S14, Supporting Information. All the P‐XRD patterns of the annealed samples showed three dominant peaks (Figure S14, Supporting Information) similar to those of amorphous‐shell@crystalline‐core materials (Figure 1B) which were attributed to the crystalline cores, however, additional new minor peaks from their corresponding shells could also be seen which indicated the crystalline nature of the shell materials as well (Figure S14, Supporting Information). The minor peaks in the P‐XRD patterns of (*C*)‐CoPO_x_@V_3%_‐Co_4_N/NF and (*C*)‐NiPO_x_@V_3%_‐Co_4_N/NF matched well with those of Co_3_(PO_4_)_2_ (PDF#01‐0373) and Ni_3_(PO_4_)_2_ (PDF#70‐1796), as shown in Figure S14, Supporting Information. While, those in the P‐XRD pattern of (*C*)‐CoNiPO_x_@V_3%_‐Co_4_N were observed to be composed of Ni_3_(PO_4_)_2_ (PDF#70‐1796), Co_3_(PO_4_)_2_ (PDF#01‐0373) and CoNiP_2_O_7_ (PDF#48‐0563) phases indicating the existence of the mixed metal‐phosphate phases. Further, we also checked the crystallinity and the internal structure of (*C*)‐CoNiPO_x_@V_3%_‐Co_4_N/NF core‐shell heterostructure using FE‐TEM (Figure S15, Supporting Information). The low‐resolution TEM images (Figure S15A_1_–A_3_, Supporting Information) showed that core‐shell heterostructure was maintained even after the heat treatment during annealing in agreement with the FE‐SEM result (Figure S13A, Supporting Information). While the HR‐TEM image (Figure S15A_4_, Supporting Information) of the shell region indicated by region 3 of Figure S15A_3_, Supporting Information, showed the existence of distinctly observable lattice fringes indicating the crystalline nature of the (*C*)‐CoNiPO_x_ shell. Further, the SAED pattern corresponding to the (*C*)‐CoNiPO_x_‐shell region showed bright dots with circular rings indicating the polycrystalline nature of the shell after annealing (Figure S15A_5_, Supporting Information). Further, the HAADF‐STEM image (Figure S15B_1_, Supporting Information) showed the core@shell heterostructures and the elemental color mapping showed the presence of Co, Ni, P, O, V, and N as its constituents and their spatial distributions (Figure S15B_2_–B_7_, Supporting Information).

### 3.1 Evaluation for OER

**Figure 5:**
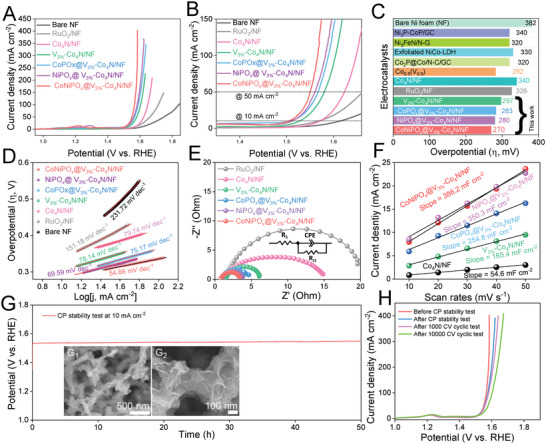
Electrochemical evaluations of the prepared electrocatalysts for OER: A) iR‐corrected LSV profiles, B) magnified LSV profiles of A for determining the overpotentials at current densities of 10 and 50 mA cm^−2^, C) overpotential comparison at 10 mA cm^−2^ with recently reported electrocatalysts such as Co_0.5_(V_0.5_),^[^ ^50^ ^]^ Co_2_P@Co/N‐C/GC,^[^ ^51^ ^]^ exfoliated NiCo‐LDH,^[^ ^52^ ^]^ Ni_3_FeN/N‐G,^[^ ^53^ ^]^ and Ni_2_P‐CoP/GC,^[^ ^54^ ^]^ D) Tafel plots, E) EIS spectra (inset shows the corresponding equivalent impedance circuit diagram),^[^ ^47^, ^48^, ^49^ ^]^ F) current density versus scan rate profiles for the evaluation of *C*_dl_, G) long‐term CP stability test of CoNiPO_x_@V_3%_‐Co_4_N/NF for 50 h duration at a current density of 10 mA cm^−2^, G_1_,G_2_) low‐ and high‐magnification FE‐SEM images of CoNiPO_x_@V_3%_‐Co_4_N/NF after CP stability test, and H) comparison of LSV profiles of CoNiPO_x_@V_3%_‐Co_4_N/NF before and after long‐term CP stability test, 1000 CV and 10 000 CV cycles.

Because OER is considered the bottleneck for the overall water splitting, the OER activity of the prepared electrocatalysts was initially evaluated using the conventional three‐electrode configuration to determine their efficacies. The iR‐corrected LSV profiles of the pristine Co_4_N/NF and V_x_‐doped Co_4_N/NF electrocatalysts, recorded at a scan rate of 2 mV sec^−1^, showed that the V_3%_‐Co_4_N/NF electrocatalyst has a much higher OER activity compared with pristine Co_4_N/NF and other concentrations of V‐doped Co_4_N/NF (V_1%_‐dCo_4_N/NF and V_5%_‐Co_4_N/NF), as shown in Figure S3A, Supporting Information, which can be attributed to its lower charge transfer resistance (*R*_ct_) (Figure S3C, Supporting Information) and higher ECSA (Figure S3D–H, Supporting Information). Therefore, V_3%_‐Co_4_N/NF sample electrocatalysts were further used as the optimized core materials for the further electrodeposition of the shell materials. The iR‐corrected LSV profiles of the electrodeposited CoNiPO_x_@V_3%_‐Co_4_N/NF electrocatalysts prepared under various electrodeposition conditions also showed the best OER performance for the CoNiPO_x_ nanosheets electrodeposited at a scan rate of 6 mV s^−1^ for two CV segments (Figure S8A, Supporting Information) owing to its ultrathin nanosheet structures, in agreement with the results of the FE‐SEM analysis (Figure S7, Supporting Information, and Figure 2C). Furthermore, the OER performance of CoNiPO_x_@V_3%_‐Co_4_N/NF was also compared with those of binary NiPO_x_@V_3%_‐Co_4_N/NF and CoPO_x_@V_3%_‐Co_4_N/NF heterostructures, along with the V_3%_‐Co_4_N/NF, Co_4_N/NF, RuO_2_/NF, and bare Ni foam, as shown in **Figure** **5**A. Among the developed electrocatalysts, the LSV profile of the CoNiPO_x_@V_3%_‐Co_4_N/NF heterostructures showed the lowest overpotential and highest current density (Figure 5A). The contribution from the redox peak current was eliminated by estimating the OER overpotentials after the redox peak potentials at current densities of 10 and 50 mA cm^−2^, as shown in the magnified LSV profiles in Figure 5B. At a current density of 10 mA cm^−2^, the overpotentials of the prepared electrocatalysts were in the order of CoNiPO_x_@V_3%_‐Co_4_N/NF (270 mV) < NiPO_x_@V_3%_‐Co_4_N/NF (280 mV) < CoPO_x_@V_3%_‐Co_4_N/NF (283 mV) < V_3%_‐Co_4_N/NF (297 mV) < RuO_2_/NF (326 mV) < Co_4_N/NF (340 mV), indicating the lowest OER overpotential for CoNiPO_x_@V_3%_‐Co_4_N/NF. Moreover, the overpotential of CoNiPO_x_@V_3%_‐Co_4_N/NF was also found to be much lower than that of many other recently reported electrocatalysts, as shown in Figure 5C and Table S1, Supporting Information, which further demonstrated its excellent OER activity. Even at the high current densities of 100 and 400 mA cm^−2^, CoNiPO_x_@V_3%_‐Co_4_N/NF showed low overpotentials of 335 mV and 353 mV, respectively, indicating its high efficacy for high‐current alkaline electrolyzers. For comparison, the LSV profiles of the crystalline‐shell@crystalline‐core heterostructured electrocatalysts denoted as (*C*)‐CoNiPO_x_@V_3%_‐Co_4_N/NF, (*C*)‐NiPO_x_@V_3%_‐Co_4_N/NF, and (*C*)‐CoPO_x_@V_3%_‐Co_4_N/NF were also recorded for OER under the same conditions, shown in Figure S16A_1_, Supporting Information. The (*C*)‐CoNiPO_x_@V_3%_‐Co_4_N/NF, (*C*)‐NiPO_x_@V_3%_‐Co_4_N/NF, and (*C*)‐CoPO_x_@V_3%_‐Co_4_N/NF electrocatalysts showed an overpotential of 287, 298, and 309 mV which are much higher than their amorphous‐shell@crystalline‐core counterparts (Figure 5C) at the same current density of 10 mA cm^−2^, thus indicating the superior electrocatalytic performance of the amorphous‐shell@crystallne‐core heterostructures (Figure S16A_1_, Supporting Information).

Further, the OER electrochemical kinetics were investigated by evaluating the Tafel slopes of the prepared electrocatalysts from the iR‐corrected LSV profiles, as shown in Figure 5D. Among the prepared electrocatalysts, CoNiPO_x_@V_3%_‐Co_4_N/NF showed the lowest Tafel slope of 54.66 mV dec^−1^ compared with those of NiPO_x_@V_3%_‐Co_4_N/NF (69.59 mV dec^−1^), CoPO_x_@V_3%_‐Co_4_N/NF (75.17 mV dec^−1^), V_3%_‐Co_4_N/NF (78.14 mV dec^−1^), Co_4_N/NF (79.74 mV dec^−1^), RuO_2_/NF (151.18 mV dec^−1^), and bare Ni foam (231.72 mV dec^−1^) (Figure 5D). The lowest Tafel slope of CoNiPO_x_@V_3%_‐Co_4_N/NF signifies its faster electrochemical kinetics for the OER compared with that of the other developed electrocatalysts. In alkaline media, the OER is generally considered to occur at an active site (*), starting with a PCET process by the aqua species that are absorbed at its surface and subsequent formation of the O—O bond, schematically represented by the following reaction steps:^[^ ^46^ ^]^

(i) $$*+OH^{-}\to *OH+e^{-}$$

(ii) $$*\mathrm{OH}+OH^{-}\to *O+H_{2}O+e^{-}$$

(iii) $$*O+OH^{-}\to *\mathrm{OOH}+e^{-}$$

(iv) $$*\mathrm{OOH}+OH^{-}\to *O_{2}+e^{-}+H_{2}O$$

(v) $$*O_{2}\to *+O_{2}$$

Furthermore, the EIS spectra of the electrocatalysts were also recorded, and their corresponding Nyquist plots were fitted with an equivalent circuit diagram similar to previous reports,^[^ ^47^, ^48^, ^49^ ^]^ as shown in Figure 5E. Compared with all the prepared samples, the amorphous‐shell@crystalline‐core CoNiPO_x_@V_3%_‐Co_4_N/NF showed the smallest semicircular region, which indicated its lowest charge‐transfer resistance (*R*_ct_) and faster electrokinetics (Figure 5E). Further, the comparison of the Nyquist plots of the crystalline‐shell@crystalline‐core heterostructures viz. (*C*)‐CoNiPO_x_@V_3%_‐Co_4_N/NF, (*C*)‐NiPO_x_@V_3%_‐Co_4_N/NF, and (*C*)‐CoPO_x_@V_3%_‐Co_4_N/NF (Figure S16A_3_, Supporting Information) showed much higher charge transfer resistances compared to those of their amorphous‐shell@crystalline‐core counterparts, which accounts for their low electrocatalytic activities. In addition to EIS, ECSA also plays a significant role in enhancing the electrocatalytic activities of electrocatalysts; therefore, the ECSA of the prepared electrocatalysts was evaluated using the conventional electric double layer method (*C*_dl_), as represented by Equation (4) in the electrochemical characterization section. The CV profiles of the electrocatalysts recorded in the non‐Faradaic potential region and their corresponding current density versus scan rate plots are shown in Figures S16A_4_ and S17, and Figure S16A_5_, Supporting Information, and Figure 5F. The obtained slope values were 386.2, 350.3, 254.8, 165.4, and 54.6 mF cm^−2^ for CoNiPO_x_@V_3%_‐Co_4_N/NF, NiPO_x_@V_3%_‐Co_4_N/NF, CoPO_x_@V_3%_‐Co_4_N/NF, V_3%_‐Co_4_N/NF, and Co_4_N/NF, respectively. While for (*C*)‐CoNiPO_x_@V_3%_‐Co_4_N/NF, (*C*)‐NiPO_x_@V_3%_‐Co_4_N/NF, and (*C*)‐CoPO_x_@V_3%_‐Co_4_N/NF, the slope values were estimated to be 234.06, 155.47, and 141.09 mF cm^−2^ respectively, (Figure S16A_5_, Supporting Information), much lower than those of CoNiPO_x_@V_3%_‐Co_4_N/NF, NiPO_x_@V_3%_‐Co_4_N/NF, and CoPO_x_@V_3%_‐Co_4_N/NF amorphous‐shell@crystalline‐core heterostructures. Because slope values are numerically equal to twice the *C*_dl_ value, the *C*_dl_ values will be half of their corresponding slope values and as the *C*_dl_ values are directly proportional to ECSA (Equation (4)), higher *C*_dl_ values indicate a higher ECSA. Accordingly, amorphous‐shell@crystalline‐core CoNiPO_x_@V_3%_‐Co_4_N/NF exhibited the highest ECSA compared with the other electrocatalysts, which also accounts for its superior OER activity. Further, to investigate the mass activity of amorphous‐shell@crystalline‐core materials, the OER LSV profiles of the electrocatalysts in Figure 5A were normalized by their corresponding mass loadings, as shown in Figure S18A_1_, Supporting Information. The mass activities of the electrocatalysts for OER were in the order of CoNiPO_x_@V_3%_‐Co_4_N/NF > NiPO_x_@V_3%_‐Co_4_N/NF > CoPO_x_@V_3%_‐Co_4_N/NF > V_3%_‐Co_4_N/NF > Co_4_N/NF respectively similar to their activity trend of geometric area normalized LSV profiles observed in Figure 5A.

To further investigate the intrinsic catalytic activities of the developed amorphous‐shell@crystalline‐core heterostructures for OER, the turnover frequency (TOF) of the electrocatalysts was also evaluated by determining the number of actives sites using CV tests as shown in Figure S19, Supporting Information, using Equations (S1) and (S2), Supporting Information, respectively. The TOF of the electrocatalysts for OER at an overpotential of 350 mV were in the order of CoNiPO_x_@V_3%_‐Co_4_N/NF (22 s^−1^) > NiPO_x_@V_3%_‐Co_4_N/NF (12.60 s^−1^) > CoPO_x_@V_3%_‐Co_4_N/NF (11.8 s^−1^) > V_3%_‐Co_4_N/NF (11.12 s^−1^) > Co_4_N/NF (10.11 s^−1^) respectively, thus showing the highest intrinsic OER catalytic activity for the CoNiPO_x_@V_3%_‐Co_4_N/NF electrocatalysts (Figure S20A_1_, Supporting Information). Further, to check the influence of O_2_ gas saturated 1 m KOH electrolyte on OER activities, the LSV profile of our best electrocatalyst CoNiPO_x_@V_3%_‐Co_4_N/NF was recorded using O_2_ saturated 1 m KOH electrolyte (Figure S20B_1_, Supporting Information). However, the result showed that there were no significant changes in the OER activity resulting from O_2_ gas saturation of the electrolyte. In fact, there is a slightly negative effect on the electrocatalyst's OER performance which could be attributed to the formation of small gas bubbles accumulating on the electrode surface in the case of the O_2_ saturated 1 m KOH electrolyte (Figure S20B_1_, Supporting Information), in agreement with the previous report.^[^ ^55^ ^]^

In addition to low overpotentials, efficient electrocatalysts should also demonstrate long‐term stability that is suitable for practical applications. Therefore, a long‐term operational stability test of the CoNiPO_x_@V_3%_‐Co_4_N/NF electrocatalyst for the OER was performed using the chronopotentiometry test (CP) test at a current density of 10 mA cm^−2^ for 50 h, as shown in Figure 5G. Under the long‐term CP stability test, CoNiPO_x_@V_3%_‐Co_4_N/NF showed almost negligible degradation in the OER performance, maintaining an almost constant potential throughout. Furthermore, the changes in the morphological features of CoNiPO_x_@V_3%_‐Co_4_N/NF after the long‐term CP stability test were also investigated using FE‐SEM, as shown in Figure 5G_1_,G_2_, which showed almost identical core‐shell structures as those of the pre‐stability test, demonstrating the morphological robustness of the CoNiPO_x_@V_3%_‐Co_4_N/NF electrocatalysts even after long‐term operation. However, in general, transition metal phosphates and nitrides are expected to undergo superficial in situ oxidation, resulting in the generation of their corresponding transition metal (oxy)hydroxides that are highly active for the OER.^[^ ^11^, ^43^, ^56^ ^]^ Therefore, after the OER stability test, the XPS technique was employed to investigate the changes in the chemical compositions and oxidation states of the CoNiPO_x_@V_3%_‐Co_4_N/NF electrocatalysts. The XPS survey spectrum of the CoNiPO_x_@V_3%_‐Co_4_N/NF after the long‐term CP stability test for OER (Figure S21A_1_, Supporting Information) showed an increase in the intensity of the O peak, whereas the peak corresponding to P was greatly reduced, thereby suggesting the in situ surface oxidation of metal phosphates to their corresponding metal(oxy)hydroxides, in agreement with various previous reports.^[^ ^11^, ^56^ ^]^ Further, the deconvoluted high‐resolution core XPS spectra of Co 2p (Figure S21A_2_, Supporting Information) and Ni 2p (Figure S21A_3_, Supporting Information) also showed much higher peak intensities corresponding to Co^3+^ and Ni^3+^ compared to those before the long‐term OER stability test (Figure 4A,B) signifying their in situ oxidation resulting to their corresponding metal(oxy)hydroxides phases, similar to previous reports.^[^ ^31^, ^43^ ^]^ In addition, the deconvoluted P 2p and O 1s XPS spectra (Figure S21A_4_,A_5_, Supporting Information) also showed a decrease in the M‐PO_x_ peak intensity after the stability test (Figure S21A_4_, Supporting Information) and much higher peak intensity indicated as O_1_ (Figure S21A_5_, Supporting Information) compared to those before the stability test (Figure 4C,D) thus indicating lowering of metal‐phosphate species and generation of their corresponding metal(oxy)hydroxides resulting from the superficial in situ oxidation of the electrocatalyst during long‐term OER evaluations, in agreement with previous reports.^[^ ^11^, ^56^ ^]^ Further, the P‐XRD pattern of CoNiPO_x_@V_3%_‐Co_4_N electrocatalyst was also recorded after the long‐term OER stability test as shown in Figure S22, Supporting Information, however, no observable peaks corresponding to their metal(oxy)hydroxides were detected in the P‐XRD pattern of the post‐OER analysis, which could be attributed to the fact that the in situ oxidations occurred superficially, in agreement with the previous reports.^[^ ^31^ ^]^ Further, to observe the change in the internal structure of the electrocatalysts, the HR‐TEM images of CoNiPO_x_@V_3%_‐Co_4_N after the OER stability test were also recorded as shown in Figure S23, Supporting Information. The low‐resolution TEM images (Figure S23A_1_–A_3_, Supporting Information) showed clear core‐shell heterostructures indicating the robustness of the heterostructure even after the long‐term CP stability test for OER. However, the HR‐TEM image (Figure S23A_4_, Supporting Information)) of region 1 of Figure S23A_3_, Supporting Information, showed partially observable lattice fringes and SAED pattern (Figure S23A_5_, Supporting Information) with circular rings and bright dots indicating a partial polycrystalline nature of the CoNiPO_x_‐shells after the long‐term OER evaluation indicating that the amorphous‐shells underwent a mild crystallization during the long‐term OER evaluations which accounts for the higher stability during the long‐term operation, in agreement to previous reports.^[^ ^9^, ^57^ ^]^ Further, HAADF‐STEM elemental color mapping showed the presence of Co, Ni, P, O, V, and N as constituent elements (Figure S23B,C, Supporting Information) and their spatial distribution. Interestingly, after the OER stability test, the intensity of the O species was much enhanced while that of the P species was much lowered (Figure S23C_3_,C_4_, Supporting Information) indicating the in situ oxidation consistent with the observation in the XPS analysis of the post‐OER stability test (Figure S21A_1_, A_4_ & A_5_, Supporting Information).^[^ ^31^ ^]^ Thus, post‐OER characterizations showed the in situ superficial oxidation of CoNiPO_x_@V_3%_‐Co_4_N/NF electrocatalysts after the long‐term OER stability test, in agreement with previous reports.^[^ ^9^, ^31^, ^43^ ^]^

The LSV profiles of the CoNiPO_x_@V_3%_‐Co_4_N/NF electrocatalysts measured after the CP stability test and after 1000 and 10 000 CV cycles were also compared with those of the pre‐stability test, as shown in Figure 5H, and no significant changes in the overpotentials were observed, demonstrating its excellent OER performance for long‐term operation. The excellent OER performance of CoNiPO_x_@V_3%_‐Co_4_N/NF electrocatalysts can be attributed to the abundant active sites resulting from the multi‐component core‐shell heterostructures, higher ECSA, lower charge‐transfer resistance, and in situ generation of metal(oxy)hydroxides due to the superficial surface oxidation during long‐term OER evaluations.

### 3.2 Evaluation for Hydrogen Evolution Reaction (HER)

**Figure 6:**
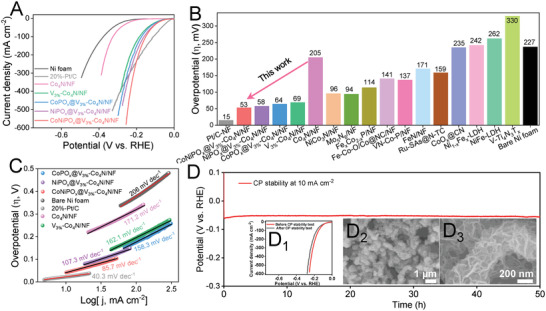
Electrochemical evaluation of the prepared electrocatalysts for HER: A) iR‐corrected LSV profiles of CoNiPO_x_@V_3%_‐Co_4_N/NF, NiPO_x_@V_3%_‐Co_4_N/NF, CoPO_x_@V_3%_‐Co_4_N/NF, V_3%_‐Co_4_N/NF, Co_4_N/NF, 20%‐Pt/C/NF, and bare Ni foam, B) comparison of overpotentials at a current density of 10 mA cm^−2^ with recently reported HER electrocatalysts such as NiCo_3_N/NF,^[^ ^58^ ^]^ Mo_5_N_6_,^[^ ^59^ ^]^ Fe_x_Co_2‐x_P/NF,^[^ ^31^ ^]^ Fe—Co—O/Co@NC/NF,^[^ ^9^ ^]^ VN‐Co‐P/NF,^[^ ^60^ ^]^ FeN/NF,^[^ ^61^ ^]^ Ru‐SAs@N‐TC,^[^ ^62^ ^]^ CoOx@CN,^[^ ^63^ ^]^ Ni_1‐x_Fe‐LDH,^[^ ^64^ ^]^ NiFe‐LDH,^[^ ^65^ ^]^ and V‐Ti_4_N_3_T_x_,^[^ ^66^ ^]^ C) Tafel plots, D) Long‐term CP stability test for 50 h at a current density of 10 mA cm^−2^, D_1_) comparison of LSV profiles before and after CP stability test, and D_2_,D_3_) low‐ and high‐magnification FE‐SEM images of CoNiPO_x_@V_3%_‐Co_4_N/NF after the long‐term CP stability test for HER.

The HER electrocatalytic activities of CoNiPO_x_@V_3%_‐Co_4_N/NF, NiPO_x_@V_3%_‐Co_4_N/NF, CoPO_x_@V_3%_‐Co_4_N/NF, V_3%_‐Co_4_N/NF, and Co_4_N/NF were also investigated using a 1 cm × 1 cm area of the as‐prepared electrocatalysts as binder‐free working electrodes directly without any polymer binders. The LSV profiles of the electrocatalysts were recorded at a slow scan rate of 2 mV s^−1^, and the iR‐corrected LSV profiles are shown in Figure 5(A) and Figures S3B and S8B, Supporting Information. The LSV profiles of V‐doped Co_4_N/NF with different concentrations of V (Figure S3B, Supporting Information) showed the optimum HER activity of V_3%_‐Co_4_N/NF (Figure S3B, Supporting Information). Accordingly, V_3%_‐Co_4_N/NF was considered the optimized core material for the electrodeposition of the CoNiPO_x_ shells. Notably, the overpotentials of the V‐doped Co_4_N/NF electrocatalysts were much lower than those of pristine Co_4_N/NF. This decrease in the overpotentials of the V‐doped Co_4_N materials can be attributed to the enhancement of HER activity due to the shifting of the d‐band center of Co_4_N resulting from V‐doping, and facilitating the hydrogen desorption process, in agreement with previous reports.^[^ ^13^ ^]^ This shows that V‐doping is an effective strategy to enhance the HER activity of the core Co_4_N electrocatalyst. Furthermore, the LSV profiles of the electrodeposited CoNiPO_x_@V_3%_‐Co_4_N/NF prepared under different electrodeposition conditions (Figure S8B, Supporting Information) also showed the best HER activity for CoNiPO_x_@V_3%_‐Co_4_N/NF prepared by electrodeposition at a scan rate of 6 mV s^−1^ for two segments, which could be ascribed to its more uniform and ultrathin nanosheet nature compared to those prepared under other conditions (Figure S7, Supporting Information, and Figure 2C). The LSV profiles of the prepared electrocatalysts, shown in **Figure** **6**A, further showed the best HER activity of CoNiPO_x_@V_3%_‐Co_4_N/NF compared to the others. At a current density of 10 mA cm^−2^, CoNiPO_x_@V_3%_‐Co_4_N/NF showed the lowest overpotential next to Pt/C, requiring only 53 mV compared to those of NiPO_x_@V_3%_‐Co_4_N/NF (58 mV), CoPO_x_@V_3%_‐Co_4_N/NF (64 mV), V_3%_‐Co_4_N/NF (69 mV), and Co_4_N/NF (205 mV) (Figure 6B). Moreover, at a high current density of 300 mA cm^−2^ and above, the CoNiPO_x_@V_3%_‐Co_4_N/NF electrocatalyst demonstrated superior HER activity, demanding much lower overpotentials than 20%‐Pt/C/NF (Figure 6A). Furthermore, the CoNiPO_x_@V_3%_‐Co_4_N/NF electrocatalyst showed better HER activity than the recently reported HER electrocatalysts (Figure 6B and Table S2, Supporting Information), indicating its excellent potential for HER in alkaline media as well. The mass activities of the electrocatalysts for HER were also determined by normalizing the geometric area‐specific LSV profiles (Figure 6A) by their corresponding mass loadings as shown in Figure S18A_2_, Supporting Information. The mass activity trend was found to be similar to that of the geometric area normalized LSV profiles, suggesting the highest mass activity for the CoNiPO_x_@V_3%_‐Co_4_N/NF. Furthermore, the intrinsic catalytic activities of the developed electrocatalysts for HER were also investigated by evaluating their corresponding TOF using Equations (S1) and (S2), Supporting Information, respectively, as shown in Figure S20A_2_, Supporting Information. The TOF of the electrocatalysts for HER at an overpotential of 350 mV was found to be in the order of CoNiPO_x_@V_3%_‐Co_4_N/NF (12.50 s^−1^) > NiPO_x_@V_3%_‐Co_4_N/NF (9.21 s^−1^) > CoPO_x_@V_3%_‐Co_4_N/NF (8.23 s^−1^) > V_3%_‐Co_4_N/NF (6.29 s^−1^) > Co_4_N/NF (1.48 s^−1^), thus signifying the highest intrinsic HER activity of the CoNiPO_x_@V_3%_‐Co_4_N/NF electrocatalyst.

For comparison, LSV profiles of the crystalline‐shell@crystalline‐core electrocatalysts denoted as (*C*)‐CoNiPO_x_@V_3%_‐Co_4_N/NF, (*C*)‐NiPO_x_@V_3%_‐Co_4_N/NF, and (*C*)‐CoPO_x_@V_3%_‐Co_4_N/NF were also recorded for HER under the same conditions, shown in Figure S16A_2_, Supporting Information. At the current density of 10 mA cm^−2^, the overpotentials of (*C*)‐CoNiPO_x_@V_3%_‐Co_4_N/NF, *C*)‐NiPO_x_@V_3%_‐Co_4_N/NF, and (*C*)‐CoPO_x_@V_3%_‐Co_4_N/NF were found to be 69.7, 86.2, and 93.1 mV respectively, much higher than those of the amorphous‐shell@crystalline‐core counterparts at the same current density. At a higher current density of 100 mA cm^−2^, (*C*)‐CoNiPO_x_@V_3%_‐Co_4_N/NF, (*C*)‐NiPO_x_@V_3%_‐Co_4_N/NF, and(*C*)‐CoPO_x_@V_3%_‐Co_4_N/N demands a much larger overpotential of 213.6, 223.2, and 232.3 mV respectively compared to their amorphous‐shell@crystalline‐core counterparts. This showed that the amorphous‐shell@crystalline‐core heterostructures have better electrocatalytic activities than their crystalline‐shell@crystalline‐core counterparts for HER similar to the observation in their OER LSV profiles (Figure S16A_1‐2_, Supporting Information).

In addition to exhibiting low overpotentials, it is equally important for electrocatalysts to exhibit fast electrokinetics for efficient HER. Tafel plots were also obtained from the corresponding iR‐corrected LSV profiles of the electrocatalysts, as shown in Figure 6C. The obtained Tafel slopes were in the order 40 mV dec^−1^ (20%‐Pt/C) < 85.7 mV dec^−1^ (CoNiPO_x_@V_3%_‐Co_4_N/NF) < 107.3 mV dec^−1^ (NiPO_x_@V_3%_‐Co_4_N/NF) < 158.3 mV dec^−1^ (CoPO_x_@V_3%_‐Co_4_N/NF) < 162.1 mV dec^−1^ (V_3%_‐Co_4_N/NF) < 171.2 mV dec^−1^ (Co_4_N/NF) < 206 mV dec^−1^ (Bare Ni foam). As seen in Figure 6C, the lowest Tafel slope of the CoNiPO_x_@V_3%_‐Co_4_N/NF electrocatalyst compared to the other prepared electrocatalysts suggests the fastest HER kinetics. Basically, the HER in alkaline media is conventionally considered to be executed in one of the two mechanisms, as represented in Equation (A) via the Volmer–Heyrovsky mechanism or Equation (B) via the Volmer–Tafel mechanism:^[^ ^9^ ^]^

(A) $$\begin{array}{ccc} & & H_{2}O+e^{-}+*\to H*+OH^{-}\left(\mathrm{Volmer}\right)\\ & & H_{2}O+e^{-}+H*\to H_{2}+OH^{-}\left(\mathrm{Heyrovsky}\right) \end{array}$$

Or,

(B) $$\begin{array}{ccc} & & H_{2}O+e^{-}+*\to H*+OH^{-}\left(\mathrm{Volmer}\right)\\ & & H*+H*\to H_{2}\left(\mathrm{Tafel}\right) \end{array}$$

where * represents the active site. In both mechanisms, initially, the adsorption of H_2_O at the active site should occur for the alkaline media, followed by the dissociation of the adsorbed H_2_O molecule into adsorbed H atoms (H*) and OH^−^, followed by the desorption of OH^−^ again to refresh the initially occupied active sites and then convert the adsorbed H atoms into gaseous H_2_ molecules, which then dissipate from the electrocatalyst surface during the reaction. Therefore, an ideal HER electrocatalyst should neither absorb H atoms (H*) too strongly nor too loosely for optimal HER. Despite there being some doubt on the exact mechanism of HER in alkaline media, the Tafel slope of 85.7 mV dec^−1^ obtained for CoNiPO_x_@V_3%_‐Co_4_N/NF, 107.3 mV dec^−1^ for NiPO_x_@V_3%_‐Co_4_N/NF, and 158.3 mV dec^−1^ for CoPO_x_@V_3%_‐Co_4_N/NF indicated that these core‐shell electrocatalysts followed a typical Volmer–Heyrovsky mechanism, as shown in Equation (A), which is in agreement with many previous reports.^[^ ^9^, ^67^ ^]^ The lower Tafel slope of V_3%_‐Co_4_N/NF compared to pristine Co_4_N/NF also demonstrated higher HER kinetics for V_3%_‐Co_4_N/NF due to V‐doping.^[^ ^13^ ^]^ Previous studies also showed that V‐doping in Co_4_N can enhance the water adsorption process, thereby facilitating the Volmer step and modulating the free energy of adsorbed H atoms (H*) (ΔG_H*_) closer to the optimum thermoneutral values compared to the pristine Co_4_N^[^ ^13^ ^]^ thus accounting for the much higher HER activities of V_3%_‐Co_4_N/NF in terms of lower overpotential and lower Tafel slope compared to those of pristine Co_4_N/NF (Figure 6A–C). Moreover, the HER performance was observed to be significantly enhanced for the electrodeposited NiPO_x_@V_3%_‐Co_4_N/NF, CoPO_x_@V_3%_‐Co_4_N/NF, and CoNiPO_x_@V_3%_‐Co_4_N/NF heterostructures, which could be attributed to the electrodeposition of a thin layer of metal‐phosphate nanosheets, boosting the HER activities as the phosphate species not only improved the water absorption but also facilitated water dissociation, thereby improving the Volmer–step.^[^ ^68^, ^69^ ^]^ The highest HER activity of CoNiPO_x_@V_3%_‐Co_4_N/NF core‐shell heterostructures compared to those of NiPO_x_@V_3%_‐Co_4_N/NF and CoPO_x_@V_3%_‐Co_4_N/NF is a result of its lower charge transfer resistance, higher ECSA, mesoporous, and uniform ultrathin nature of the nanosheets, which provide maximum electrode‐electrolyte interactions and abundant active sites for HER. Further, the HER LSV profile of our best electrocatalyst CoNiPO_x_@V_3%_‐Co_4_N/NF was also recorded using H_2_ saturated 1 m KOH electrolyte to check the influence of the H_2_ gas saturation on HER activity (Figure S20B_2_, Supporting Information). However, CoNiPO_x_@V_3%_‐Co_4_N/NF showed almost the same HER activity in the H_2_ saturated 1 m KOH electrolyte as that of the normal 1 m KOH electrolyte suggesting that there is no significant influence of the H_2_ gas saturation on the HER activity of the electrocatalyst. Furthermore, the long‐term HER performance of CoNiPO_x_@V_3%_‐Co_4_N/NF heterostructures was investigated by subjecting it to continuous operation for a duration of 50 h to generate a current density of 10 mA cm^−2^ using the CP technique. A negligible change in the potential was observed even after 50 h, indicating its robust stability (Figure 6D). The LSV profiles of CoNiPO_x_@V_3%_‐Co_4_N/NF recorded before and after the CP stability shown in Figure 6D_1_ also indicate only a slight increase in overpotential after the long‐term CA stability test at a current density of 10 mA cm^−2^. Furthermore, FE‐SEM images of the CoNiPO_x_@V_3%_‐Co_4_N/NF electrocatalyst (Figure 6D_2_,D_3_) recorded after the CP stability test also showed similar morphological features as those before the CA stability test, indicating its robust morphological stability even after long‐term evaluations for HER. Further, P‐XRD pattern and the HR‐TEM images of the CoNiPO_x_@V_3%_‐Co_4_N/NF after the long‐term stability test for HER were also recorded to investigate the phase and the internal structure (Figures S22 and S24, Supporting Information). The P‐XRD result showed no observable change in the P‐XRD patterns after the HER stability test, similar to the post‐OER observation. Moreover, the TEM images (Figure S24A_1_–A_3_, Supporting Information), HAADF‐STEM images (Figure S24A_4_, Supporting Information) along with elemental color mapping (Figure S24B_1_–B_6_, Supporting Information) showed robust core‐shell heterostructures and spatial distribution of Co, Ni, P, O, V, and N as its constituent elements even after the long‐term HER stability test thus indicating the robustness of the developed core‐shell heterostructures. Thus, the developed electrodes exhibited low overpotentials and robust stability, indicating their suitability for efficient HER in alkaline media and as a replacement for the costly and scarce Pt‐based compounds.

### 3.3 Evaluation of Overall Water Splitting

**Figure 7:**
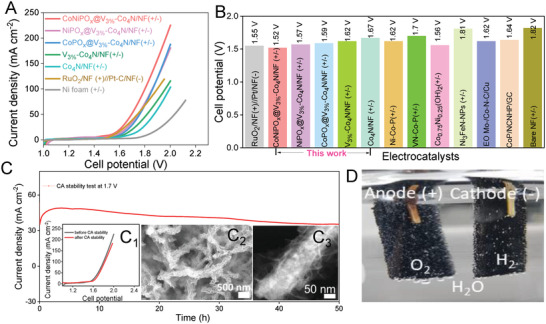
Electrochemical evaluation for overall water splitting: A) LSV profiles of CoNiPO_x_@V_3%_‐Co_4_N/NF(+/−), NiPO_x_@V_3%_‐Co_4_N/NF(+/−), CoPO_x_@V_3%_‐Co_4_N/NF(+/−), V_3%_‐Co_4_N/NF(+/−), Co_4_N/NF(+/−), RuO_2_/NF(+)/Pt‐C/NF(−) and Ni foam (+/−) electrolyzers, B) comparison of the overpotentials at the current density of 10 mA cm^−2^ of the prepared electrocatalysts with recently reported alkaline electrolyzers such as Ni‐Co‐P/NF (+/−),^[^ ^70^ ^]^ VN‐Co‐P (+/−),^[^ ^60^ ^]^ Co_0.75_Ni_0.25_(OH)_2_,^[^ ^71^ ^]^ Ni_3_FeN‐NPs,^[^ ^72^ ^]^ EO Mo‐/Co—N—C/Cu,^[^ ^73^ ^]^ and CoP/NCNHP/GC,^[^ ^74^ ^]^ respectively, C) long‐term stability of CoNiPO_x_@V_3%_‐Co_4_N/NF(+/−) at an applied potential of 1.7 V, C_1_) LSV profiles before and after CA stability test, C_2_) FE‐SEM image, and C_3_) HAADF‐STEM image of CoNiPO_x_@V_3%_‐Co_4_N/NF after CA stability test, and D) digital photographic image of the fabricated CoNiPO_x_@V_3%_‐Co_4_N/NF (+/−) alkaline electrolyzer showing the evolution of O_2_ and H_2_ gas bubbles at the anode and cathode, respectively.

Considering the excellent OER and HER bifunctional properties of the developed amorphous‐shell@crystalline‐core heterostructured electrocatalysts, various electrolyzers were assembled using them as anodes and cathodes: CoNiPO_x_@V_3%_‐Co_4_N/NF (+/−), NiPO_x_@V_3%_‐Co_4_N/NF (+/−), CoPO_x_@V_3%_‐Co_4_N/NF (+/−), V_3%_‐Co_4_N/NF (+/−), and Co_4_N/NF (+/−). In addition, an alkaline electrolyzer was assembled using a 20% wt. Pt/C/NF as the cathode and RuO_2_/NF as the anode (denoted as RuO_2_/NF(+)/20%‐Pt/C/NF(−)) as the state‐of‐the‐art electrolyzers for comparison. The LSV profiles of the developed electrolyzers at a scan rate of 2 mV s^−1^ in the potential window of 1–2 V are shown in **Figure** **7**A, which demonstrate the maximum current density and lowest cell potentials at all current densities for the CoNiPO_x_@V_3%_‐Co_4_N/NF (+/−) electrolyzer compared to those of other developed electrolyzers. The CoNiPO_x_@V_3%_‐Co_4_N/NF (+/−) electrolyzer demands only a very low cell potential of 1.52 V to generate a current density of 10 mA cm^−2^, which is lowest compared to those of NiPO_x_@V_3%_‐Co_4_N/NF (+/−) (1.57 V), CoPO_x_@V_3%_‐Co_4_N/NF (+/−) (1.59 V), V_3%_‐Co_4_N/NF (+/−)(1.62 V), and Co_4_N/NF (+/−) (1.67 V) electrolyzers (Figure 7B). The overall cell potential of the electrocatalytic water splitting exhibited by the electrolyzers at a current density of 10 mA cm^−2^ was in the order CoNiPO_x_@V_3%_‐Co_4_N/NF (+/−) < RuO_2_/NF(+)/Pt‐C/NF(−) < NiPO_x_@V_3%_‐Co_4_N/NF (+/−) < CoPO_x_@V_3%_‐Co_4_N/NF (+/−) < V_3%_‐Co_4_N/NF (+/−) < Co_4_N/NF (+/−) < Ni foam (+/−). In addition, the cell potentials of the CoNiPO_x_@V_3%_‐Co_4_N/NF (+/−) electrolyzer are superior to the state‐of‐the‐art RuO_2_/NF(+)/20%‐Pt/C/NF(−) electrolyzer at all current densities (Figure 7A,B). Even at a high current density of 100 mA cm^−2^, the CoNiPO_x_@V_3%_‐Co_4_N/NF (+/−) electrolyzer demanded only 1.79 V which is 110 mV lower than that of the RuO_2_/NF(+)/Pt‐C/NF(−) electrolyzer (1.90 V), indicating its suitability for the development of high‐current alkaline electrolyzers. On the other hand, the electrolyzer assembled from bare Ni foam substrate (Ni foam (+/−)) required an extremely large cell potential of 1.82 V compared to the 1.52 V required by CoNiPO_x_@V_3%_‐Co_4_N/NF (+/−) for generating a current density of 10 mA cm^−2^, which suggests that the overall electrocatalytic activities of CoNiPO_x_@V_3%_‐Co_4_N/NF (+/−) were solely from the assembled heterostructured CoNiPO_x_@V_3%_‐Co_4_N active materials and not from the Ni foam substrate. Furthermore, a comparison of the electrocatalytic overall water‐splitting performances of the developed electrolyzers with various other recently reported alkaline electrolyzers, as shown in Figure 7(B) and Table S3, Supporting Information, also demonstrated the lowest cell potential for the CoNiPO_x_@V_3%_‐Co_4_N/NF (+/−) electrolyzer, suggesting its superior bifunctional electrocatalytic activities from the recently reported alkaline electrolyzers. Additionally, the long‐term durability of the developed CoNiPO_x_@V_3%_‐Co_4_N/NF (+/−) alkaline electrolyzer was also investigated under continuous operation, supplying an applied potential of 1.7 V using the chronoamperometric (CA) technique for 50 h (Figure 7C). As can be seen from the figure, the current density of the CoNiPO_x_@V_3%_‐Co_4_N/NF (+/−) electrolyzer gradually increased during the initial hours of operation, stabilized over time, and deteriorated minimally even up to 50 h. The increase in the initial current density can be attributed to the in situ superficial oxidation of the CoNiPO_x_ shell, resulting in the generation of highly active metal (oxy)hydroxides during the long‐term stability test, which then stabilized gradually afterward, in agreement with previous reports.^[^ ^14^ ^]^ The LSV profile of the CoNiPO_x_@V_3%_‐Co_4_N/NF(+/−) electrolyzer recorded after the long‐term CA stability test showed negligible changes in the cell potential (Figure 7C_1_), which in addition to its lower cell potential requirement, indicated its superior stability for long‐term practical application.

In addition to the low overpotentials and long‐term stability, it is also critical to investigate the selectivity of the developed electrocatalysts for OER and HER so that the input energy used for the electrolysis is not wasted on other side reactions such as corrosion reactions or hydrogen peroxide (H_2_O_2_) generation. Therefore, Faradic efficiency (FE) of the best performing CoNiPO_x_@V_3%_‐Co_4_N/NF (+/−) alkaline electrolyzer was also evaluated using a lab‐assembled setup at an applied current density of 100 mA cm^−2^ for 1 h, as shown in Figure S25A_1_, Supporting Information. The volume‐time graphs for the measured O_2_ and H_2_ gas were compared to their corresponding theoretically computed values obtained using Equations (S_3_) and (S_4_), Supporting Information, respectively, as shown in Figure S25A_2_, Supporting Information, which demonstrated that the evolved gas volumes were very close to the theoretically computed values. This agreement of the experimental and the theoretical O_2_ and H_2_ gas volumes suggests that the FE is almost 100% for both OER and HER along with the ratio of H_2_ to O_2_ gas volume being almost 2:1 (Figure S25A_2_, Supporting Information). Further, the electrocatalytic activities of such developed electrocatalysts could also be improved by enhancing the triple‐phase boundary via increasing reaction sites, conductivities of catalyst, and choosing appropriate substrates in a more compact electrolyzer architecture similar to those of proton exchange membrane electrolyzer cells (PEMECs).^[^ ^75^, ^76^, ^77^, ^78^ ^]^

In addition, to investigate any morphological changes after the long‐term operation of alkaline electrolyzer, the FE‐SEM images of the CoNiPO_x_@V_3%_‐Co_4_N/NF electrocatalyst after the CA stability were examined which further showed almost identical morphology as that of the pre‐stability test (Figure 7C_2_ & Figure 2C). The HAADF‐STEM image also demonstrated an identical core‐shell structure without any significant changes, indicating its structural robustness (Figure 7C_3_). The evolution of O_2_ and H_2_ gas bubbles at the anode and cathode, which can be seen clearly in the photographic image in Figure 7D, indicates that the dissipation of the gas bubbles was also facilitated by the highly porous Ni foam substrates. Thus, electrochemical evaluations of the OER and HER, and the overall water‐splitting activities of the developed electrocatalysts indicated that the developed core‐shell heterostructures were highly efficient and that the employed method is highly effective for developing non‐precious earth‐abundant metal‐based bifunctional electrocatalysts for water splitting in alkaline media.

## 4 Mechanistic Study Using Density Functional Theory (DFT)

**Figure 8:**
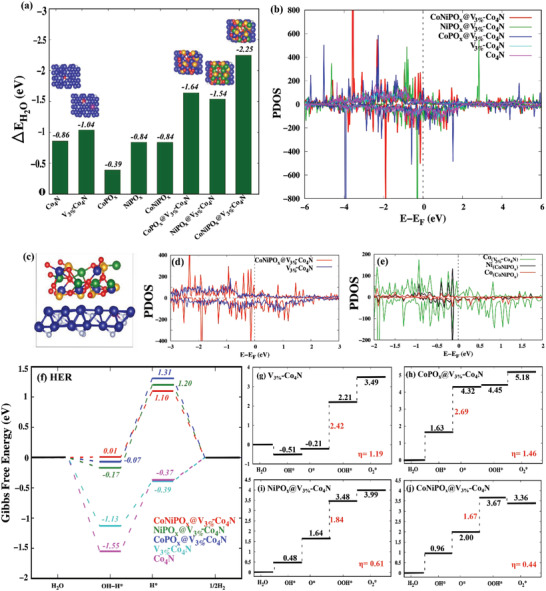
a) Adsorption energies of H_2_O on core Co_4_N, V_3%_‐Co_4_N; amorphous‐shell of CoPO_x_, NiPO_x_, CoNiPO_x,_ and core‐shell heterostructures of CoPO_x_@V_3%_‐Co_4_N, NiPO_x_@V_3%_‐Co_4_N and CoNiPO_x_@V_3%_‐Co_4_N, insets showing corresponding adsorption models of core and core‐shell heterostructures, b) electronic density of states of core Co_4_N, V_3%_‐Co_4_N, and core‐shell heterostructures CoPO_x_@V_3%_‐Co_4_N, NiPO_x_@V_3%_‐Co_4_N, CoNiPO_x_@V_3%_‐Co_4_N, c) atomic model of CoNiPO_x_@V_3%_‐Co_4_N, d) PDOSs of core‐shell heterostructure CoNiPO_x_@V_3%_‐Co_4_N with its corresponding core V_3%_‐Co_4_N, e) PDOS plots showing the contribution of Co and Ni in CoNiPO_x_@V_3%_‐Co_4_N core‐shell heterostructure, f) the calculated Gibbs free energy diagram of HER in alkaline condition, g–j) Free energy diagram of OER in alkaline condition on V_3%_‐Co_4_N, CoPO_x_@V_3%_‐Co_4_N, NiPO_x_@V_3%_‐Co_4_N, and CoNiPO_x_@V_3%_‐Co_4_N respectively, with inset showing the corresponding overpotential for OER.

DFT calculations were employed to investigate the modulation in the electronic structures resulting from the V‐doping and synergistic effect of the core and the shell materials in the amorphous‐shell@crystalline‐core heterostructures and their effect on the electrocatalytic activities for OER and HER in comparison to their crystalline‐core and amorphous shell counterparts (**Figure** **8**, Figures S26–S33, Supporting Information), similar to previous report.^[^ ^79^ ^]^ The models representing crystalline Co_4_N and V‐doped Co_4_N core are shown in Figure S26, Supporting Information, which reveals the significance of vanadium dopants in water adsorption. The adsorption energy of water on different doping concentrations of vanadium (V_x%_‐Co_4_N, x = 1,3 and 5 at %) is found to be more favorable at the vanadium sites, as can be seen in Figure S27, Supporting Information. However, compared to other concentrations of V‐doping, it can be seen that V_3%_‐Co_4_N, in particular, shows the most exothermic water adsorption indicated by its more positive d‐band center as compared to Co_4_N (−0.96 vs −1.01) (Figure S27, Supporting Information). The higher water adsorption efficacy on V_3%_‐Co_4_N explains our rationale behind considering 3% vanadium doping on Co_4_N as the optimized core material, which corroborates our experimental results as well (Figure S3B, Supporting Information). Further, the Gibbs free energy of V_3%_‐Co_4_N for OER and HER shown in Figure S28 & Table S4, Supporting Information, shows that HER is preferred at Co‐sites while OER is preferred at V‐sites, which also accounts for its superior bifunctional activities compared to those of bare Co_4_N as observed experimentally (Figure 6A,B). Likewise, the atomic models of our amorphous CoPO_x_, NiPO_x_, and CoNiPO_x_ shell shown in Figures S29–S31, Supporting Information, display defective structures with unsaturated Co, Ni, and Co—Ni centers with highly diffused electronic states near the Fermi level. These diffused electronic states enhance the carrier density leading to higher electrocatalytic performance when compared to the corresponding crystalline structures. Furthermore, the atomic models of core‐shell heterostructures are designed, as shown in Figure 8(c) & Figure S32, Supporting Information, and the synergistic effect of crystalline V_3%_‐Co_4_N core and amorphous CoPO_x_, NiPO_x,_ and CoNiPO_x_ shells are then studied. It can be observed from Figure 8(a) that the water adsorption energies on the amorphous‐shell@crystalline‐core heterostructures are higher (i.e., more negative) than the individual crystalline‐core and amorphous‐shell materials. This corroborates to a higher density of electronic states near the Fermi level in the amorphous‐shell@crystalline‐core heterostructures which can be seen in Figure 8(b). The projected density of states (PDOS) plot in Figure 8(d) reveals the enhancement of electronic and carrier density near the Fermi level in CoNiPO_x_@V_3%_‐Co_4_N as compared to V_3%_‐Co_4_N, thereby indicating the synergistic improvement in conductivity and electrocatalytic performance after the amorphous‐shell@crystalline‐core nano‐assembly. Upon further analysis, a higher density of Ni‐d orbitals has been observed near the Fermi level in Figure 8(e) which further attributes to higher carrier density and water adsorption in the CoNiPO_x_@V_3%_‐Co_4_N heterostructure, which accounts for its higher activity than CoPO_x_@V_3%_‐Co_4_N and NiPO_x_@V_3%_‐Co_4_N. Since HER in alkaline conditions involves H_2_O adsorption prior to O—H bond cleavage (Volmer step) to release H_2_ (Heyrovsky or Tafel step), it is crucial to investigate the H_2_O adsorption, H_2_O dissociation (G_OH‐H*_) and H adsorption (G_H*_) free‐energies to understand the underlying mechanism for the superior HER electrocatalytic activity of CoNiPO_x_@V_3%_‐Co_4_N heterostructure.

As can be seen in Figure 8(a), CoNiPO_x_@V‐_3%_Co_4_N heterostructure possesses the highest efficacy for water adsorption (−2.25 eV) owing to enhanced electron density from Ni and unsaturated Co atoms on the CoNiPO_x_ surface. Further, we have also evaluated G_OH‐H*_ and G_H*_ as they are considered important descriptors for HER activity in alkaline media (Figure 8(f)). The calculated ΔG_OH‐H*_ values of CoNiPO_x_@V_3%_‐Co_4_N, NiPO_x_@V_3%_‐Co_4_N, and CoPO_x_@V_3%_‐Co_4_N heterostructures are 0.01, −0.17, and −0.07 eV respectively which are close to zero, and that of V_3%_‐Co_4_N and Co_4_N are −1.13 and −1.55 eV respectively. While the corresponding ΔG_H*_ values of CoNiPO_x_@V_3%_‐Co_4_N, NiPO_x_@V_3%_‐Co_4_N, CoPO_x_@V_3%_‐Co_4_N, V_3%_‐Co_4_N, and Co_4_N are 1.10, 1.20, 1.31, −0.39, and −0.37 eV respectively. Since electrocatalysts with ΔG_OH‐H*_ and ΔG_H*_ close to zero are considered excellent catalysts for HER in alkaline conditions, the CoNiPO_x_@V_3%_‐Co_4_N, NiPO_x_@V_3%_‐Co_4_N, CoPO_x_@V_3%_‐Co_4_N amorphous‐shell@crystalline‐core heterostructures with ΔG_OH‐H*_ and ΔG_H*_ close to zero can be served as excellent catalysts for HER in alkaline condition, in agreement with the experimental results (Figure 6A,B). The lower ΔG_OH‐H*_ values in the core‐shell heterostructures, particularly, CoNiPO_x_@V_3%_Co_4_N imply that the cleavage of O—H bond can lead to a significant increase of H^+^ concentration which would accentuate H_2_ formation and accelerate HER. Therefore, in our study, the H_2_O dissociation and G_OH‐H*_ (Volmer step) has been observed to be more crucial in influencing the HER activity, thus the heterostructures show a higher electrocatalytic performance as compared to V_3%_‐Co_4_N and Co_4_N.

Furthermore, the four‐electron OER has been analyzed through the formation of OH*, O*, and OOH* intermediates and the Gibbs free energies of these intermediates. Excellent performance in OER can be achieved when the interaction between the intermediates and the substrates is not too strong or too weak. In short, the Gibbs free energy of each step should be closer to 1.23, 2.46, 3.69, and 4.92 eV, respectively to achieve optimal OER.^[^ ^80^ ^]^ The catalytic activities of the amorphous‐shell@crystalline‐core heterostructures and V_3%_‐Co_4_N for OER have been investigated as shown in Figure 8 (g–j). While V_3%_‐Co_4_N shows weak adsorption of OH* intermediate, all heterostructures show minimal adsorption which is preferred for OER as a strong OH* adsorption can deactivate the catalyst. The consecutive reaction steps become uphill on all systems with the highest energy barrier in the third OOH* intermediate step for V_3%_‐Co_4_N (2.42 eV), NiPO_x_@V_3%_‐Co_4_N (1.84 eV), and CoNiPO_x_@V_3%_‐Co_4_N (1.67 eV) while the second O* intermediate step is the limiting barrier for CoPO_x_@V_3%_‐Co_4_N (2.69 eV). Hence, the calculated ΔG_max_ for OER on CoNiPO_x_@V_3%_‐Co_4_N, NiPO_x_@V_3%_‐Co_4_N, CoPO_x_@V_3%_‐Co_4_N, and V_3%_‐Co_4_N are 1.67, 1.84, 2.69, and 2.42 eV respectively, while the corresponding overpotentials are 0.44, 0.61, 1.46, and 1.19 eV thus showing the highest activity for CoNiPO_x_@V_3%_‐Co_4_N in agreement with the experimental results for OER (Figure 5A–C). The improvement in the OER performance of the amorphous‐shell@crystalline‐core heterostructure can be attributed to unsaturated Co and Ni in the amorphous shell. It is also important to note that the OER performance of the amorphous‐shell@crytalline‐core CoNiPO_x_@V_3%_‐Co_4_N heterostructure is higher than its corresponding CoNiPO_x_ amorphous‐shell (Figure S33, Supporting Information) and the V_3%_‐Co_4_N crystalline‐core, (Figure S28, Supporting Information). Thus, DFT calculations reveal cooperative synergism between crystalline V_3%_‐Co_4_N core and amorphous CoNiPO_x_ shell thereby promoting water adsorption, dissociation, and optimal adsorption of the reaction intermediates leading to improved HER and OER performance in alkaline media.

## 5 Conclusion

In summary, amorphous and ultrathin 2D CoNiPO_x_ nanosheet arrays directly anchored on crystalline 1D V‐doped Co_4_N nanowires we were judiciously developed to obtain amorphous‐shell@crystalline‐core (CoNiPO_x_@V_3%_‐Co_4_N) 3D heterostructures on a conductive Ni foam substrate with a high SSA and mesoporous nature for maximizing the synergistic effect of the efficient OER activity of bimetallic metal phosphate with high HER activity of V‐doped cobalt nitride in tandem for efficient overall water splitting in alkaline media. Because of the abundant active sites, enhanced ECSA, lowest charge‐transfer kinetics, and the synergistic effect between the amorphous‐shell and the crystalline‐core, the developed CoNiPO_x_@V_3%_‐Co_4_N heterostructures demonstrated excellent OER and HER bifunctional activities, requiring a low overpotential for OER and HER and providing long‐term stability. Furthermore, the configuration of amorphous shells over the crystalline core also accounted for high electrocatalytic activities and long‐term stability owing to surface‐ and volume‐confined electrocatalysis resulting from the amorphous‐shell@crystalline‐core configuration. Moreover, the alkaline electrolyzer composed of CoNiPO_x_@V_3%_‐Co_4_N/NF as both anode and cathode (CoNiPO_x_@V_3%_‐Co_4_N/NF (+/−)) showed excellent overall water‐splitting activity by requiring only a low cell‐potential of 1.52 V, which is much lower than that of the 20%Pt/C (−)//RuO_2_ (+) electrolyzer with robust stability. The mechanism for the superior OER and HER bifunctional activities of the developed electrocatalysts was also investigated using DFT‐based theoretical calculations, which corroborated well with our experimental results. Thus, this study reveals that the formation of an amorphous shell over the crystalline core can be an effective strategy for the designed synthesis of next‐generation high‐performance amorphous‐shell@crystalline‐core heterostructures of various metal nitrides and metal phosphates as excellent bifunctional electrocatalysts for H_2_ production in alkaline water splitting.

## Conflict of Interest

The authors declare no conflict of interest.

## Supporting information

Supporting Information

## Data Availability

Research Data are not shared.
