# Supplementary material for: High‐Alkaline Water‐Splitting Activity of Mesoporous 3D Heterostructures: An Amorphous‐Shell@Crystalline‐Core Nano‐Assembly of Co‐Ni‐Phosphate Ultrathin‐Nanosheets and V‐ Doped Cobalt‐Nitride Nanowires
Source: Adv Sci (Weinh). 2022 Jun 6;9(23):2201311. doi: 10.1002/advs.202201311 (PMC9376825; doi:10.1002/advs.202201311)
Supplement: Supplementary file 1 — Supporting Information [file ADVS-9-2201311-s001.pdf]

ADVANCED SCIENCE
Open Access

Supporting Information

for *Adv. Sci.*, DOI 10.1002/advs.202201311

High-Alkaline Water-Splitting Activity of Mesoporous 3D Heterostructures: An Amorphous-Shell@Crystalline-Core Nano-Assembly of Co-Ni-Phosphate Ultrathin-Nanosheets and V- Doped Cobalt-Nitride Nanowires

*Thangjam Ibomcha Singh, Ashakiran Maibam, Dun Chan Cha, Sunghoon Yoo, Ravichandar Babarao, Sang Uck Lee and Seunghyun Lee**

Supporting Information

High-alkaline Water-splitting Activity of Mesoporous 3D Heterostructures: An amorphous-shell@crystalline-core Nano-assembly of Co-Ni-phosphate Ultrathin-nanosheets and V-doped Cobalt-nitride Nanowires

Thangjam Ibomcha Singh, Ashakiran Maibam, Dun Chan Cha, Sunghoon Yoo, Ravichandar Babarao, Sang Uck Lee, Seunghyun Lee.*

T. I. Singh, S. U. Lee, S. Lee
Department of Chemical and Molecular Engineering, Hanyang University ERICA, Ansan, 15588, Republic of Korea
E-mail: leeshyun@hanyang.ac.kr

A. Maibam, R. Babarao
School of Science, RMIT University, Melbourne 3001, Victoria, Australia

A. Maibam
Physical and Materials Division, CSIR-National Chemical Laboratory, Pune 411 008, India

A. Maibam
Academy of Scientific and Innovative Research, CSIR-Human Resource Development Centre (CSIR-HRDC) Campus, Postal Staff College Area, Ghaziabad, 201002, Uttar Pradesh, India

R. Babarao
Manufacturing, CSIRO, Normanby Road, Clayton 3168, Victoria, Australia

T. I. Singh, D. C. Cha, S. Yoo, S. U. Lee, S. Lee
Center for Bionano Intelligence Education and Research, Hanyang University ERICA, Ansan, 15588, Republic of Korea

D. C. Cha, S. Yoo, S. U. Lee, S. Lee
Department of Applied Chemistry, Hanyang University ERICA, Ansan, 15588, Republic of Korea

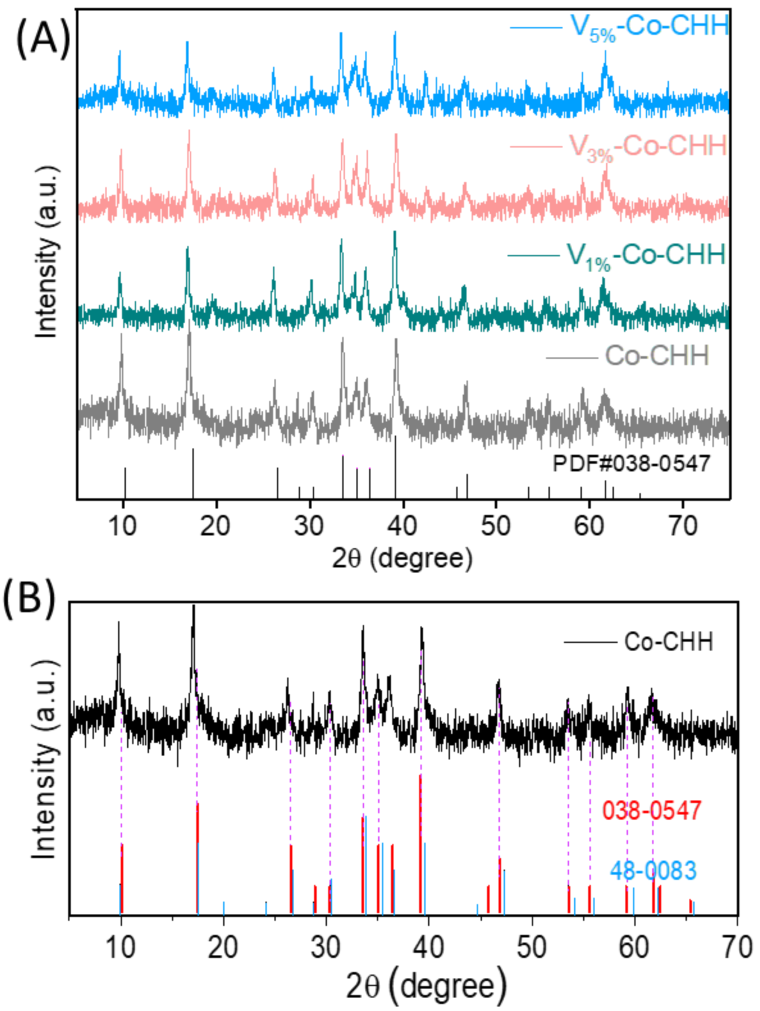

Figure S1. (A) P-XRD patterns of hydrothermally prepared pristine Co-CHH and V_x-Co-CHH nanowires (x= 1,3, 5 at. %) and (B) comparison of P-XRD patterns of hydrothermally prepared pristine Co-CHH with the stick patterns of $Co(CO_3)_{0.5}OH\cdot0.11H_2O$ (JCPDS card no. 48-0083)[1] and $Co_6(CO_3)_2(OH)_8.H_2O$ which is isostructural to $Co(Co_3)_{0.35}Cl_{0.20}(OH)_{1.10}.1.74H_2O$ (PDF card no. 038-0547),[2] confirming that the correct phase is those of (PDF card no. 038-0547).

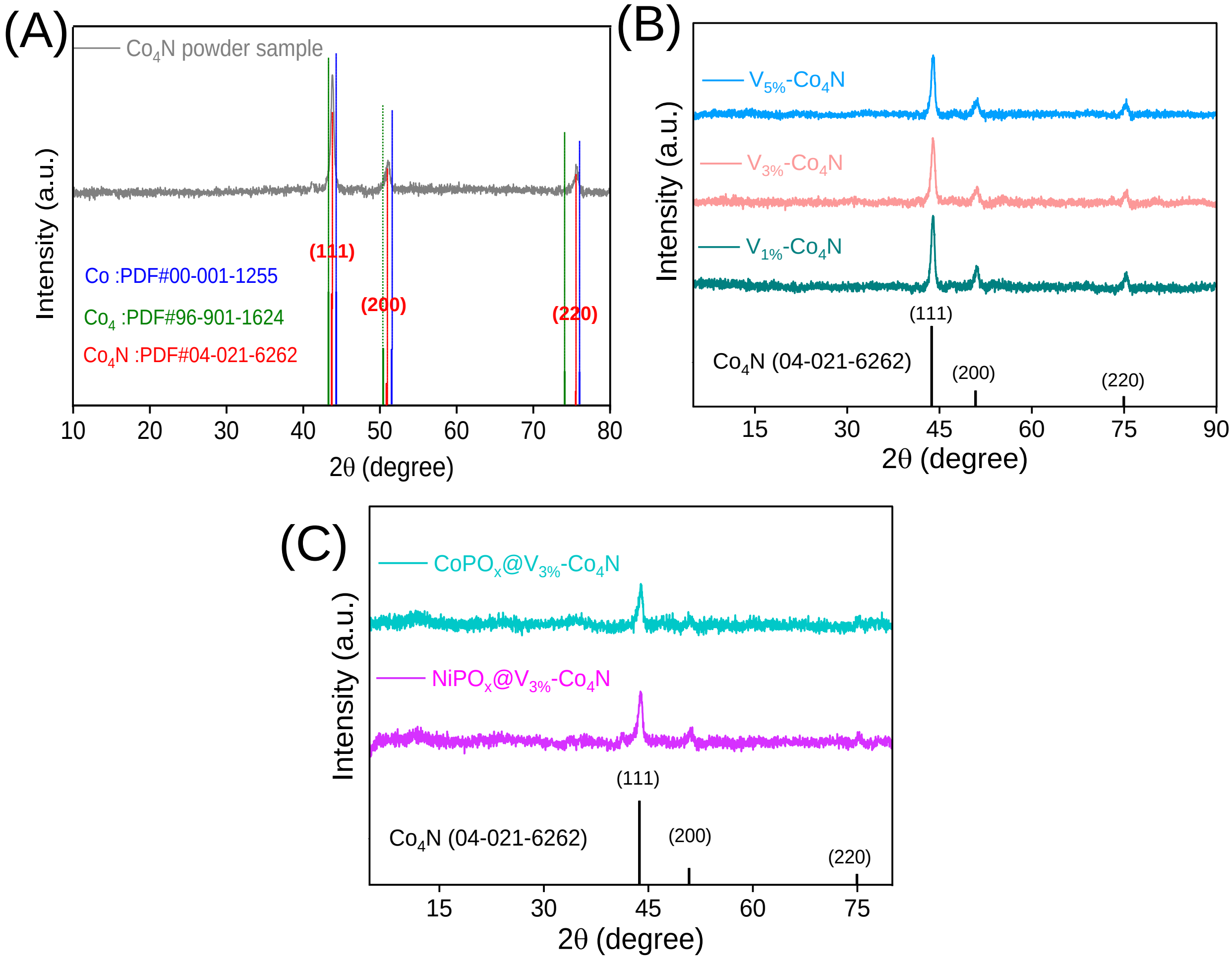

Figure S2. P-XRD patterns of (A) Co_4N nanowires compared with the standard XRD stick patterns of metallic Co (PDF#00-001-1255), Co_4 (PDF#96-901-1624) and Co_4N (PDF#04-021-6262) phases respectively, which shows that the obtained P-XRD pattern was more closely matched to those of Co_4N (PDF#04-021-6262), confirming the formation of Co_4N phase and not metallic Co, (B)V_x-doped Co_4N nanowires (x =1, 3, and 5 at. %) and (C) electrodeposited $CoPO_x$@$V_{3\%}$-Co_4N and $NiPO_x$@$V_{3\%}$-Co_4N heterostructures showing the amorphous nature of electrodeposited $CoPO_x$ and $NiPO_x$ nanosheet shells.

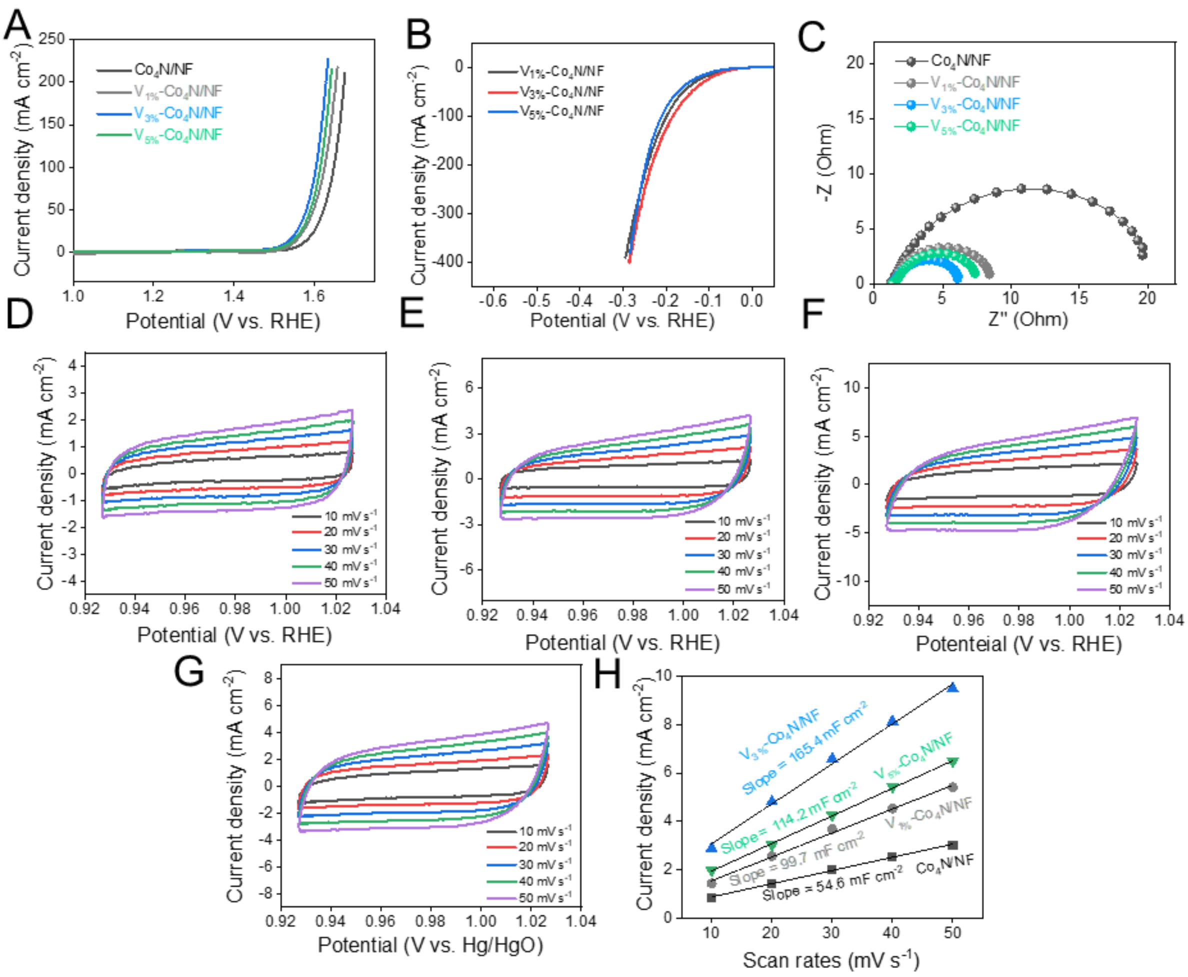

Figure S3. Electrochemical evaluations of V_x-Co_4N/NF (x =0, 1, 3, and 5 at. %) electrocatalysts for evaluating the optimum V doping concentration: LSV profiles of V_x-Co_4N/NF electrocatalysts for OER (A), and HER (B), Nyquist plots (C), CV profiles at various scan rates ranging from 10-50 mV s^{-1} in the non-faradaic region for pristine Co_4N (D), $V_{1\%}$- Co_4N (E), $V_{3\%}$- Co_4N (F), $V_{5\%}$-Co_4N (G) and their corresponding current vs. scan rates curves indicating the best electrocatalytic activity for $V_{3\%}$-Co_4N/NF (H).

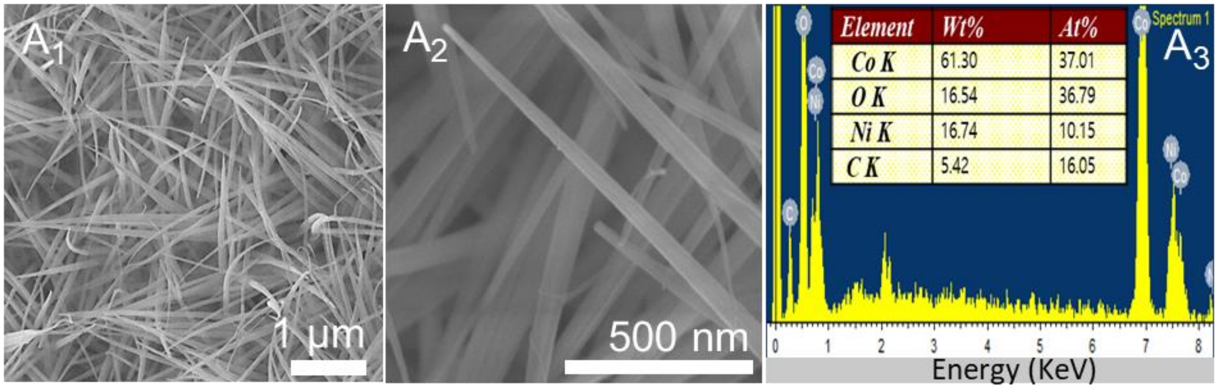

Figure S4. (A_1-A_2) Low- and high-magnification FE-SEM images of Co-CHH/NF and (A_3) EDS spectra showing the constituent elements. The presence of Ni in EDS spectra results from the Ni foam substrate and not from the Co-CHH nanowires.

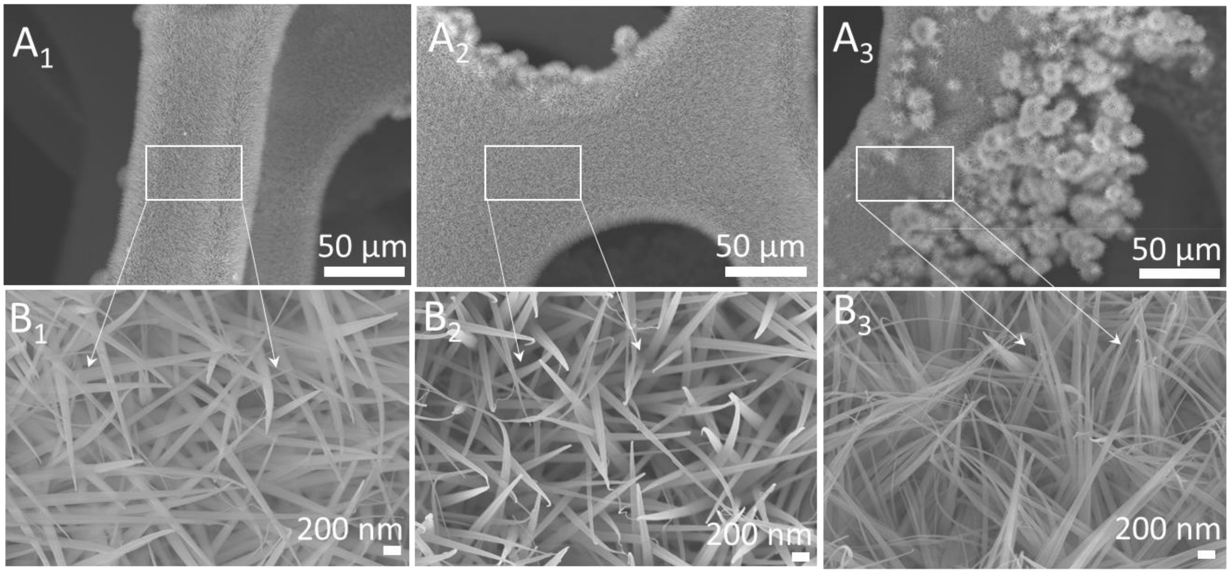

Figure S5. Low- and high-magnification FE-SEM images of V_x-Co-CHH (x = 1, 3, and 5 at. %) nanowires on Ni foam: (A_1-B_1) 1%, (A_2-B_2) 3%, and (A_3-B_3) 5%, respectively.

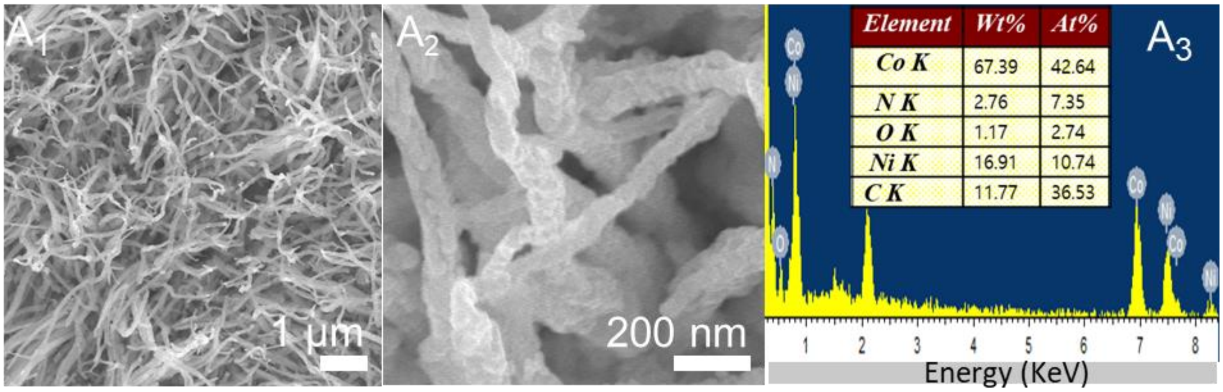

Figure S6. (A_1-A_2) Low-and high-magnification FE-SEM image of pristine Co-CHH/NF derived Co_4N/NF nanowires and corresponding EDS spectra showing the atomic concentration of its constituent elements.

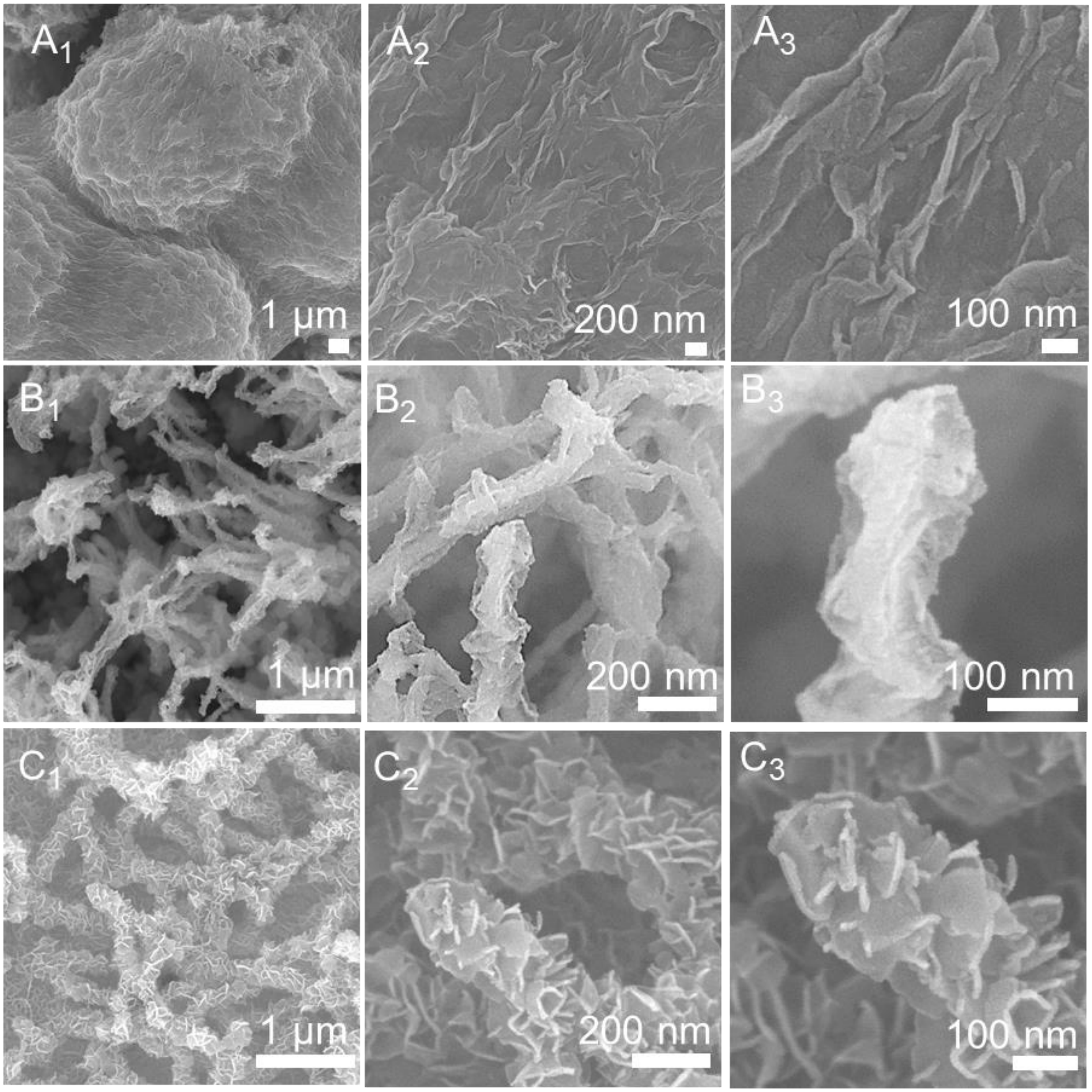

Figure S7. Low- and high-magnification FE-SEM images of electrodeposited $CoNiPO_x$ shell through CV technique at scan rates of (A) 3 mV S^{-1}, (B) 5 mV s^{-1}, and (C) 7 mV s^{-1} for two segments respectively.

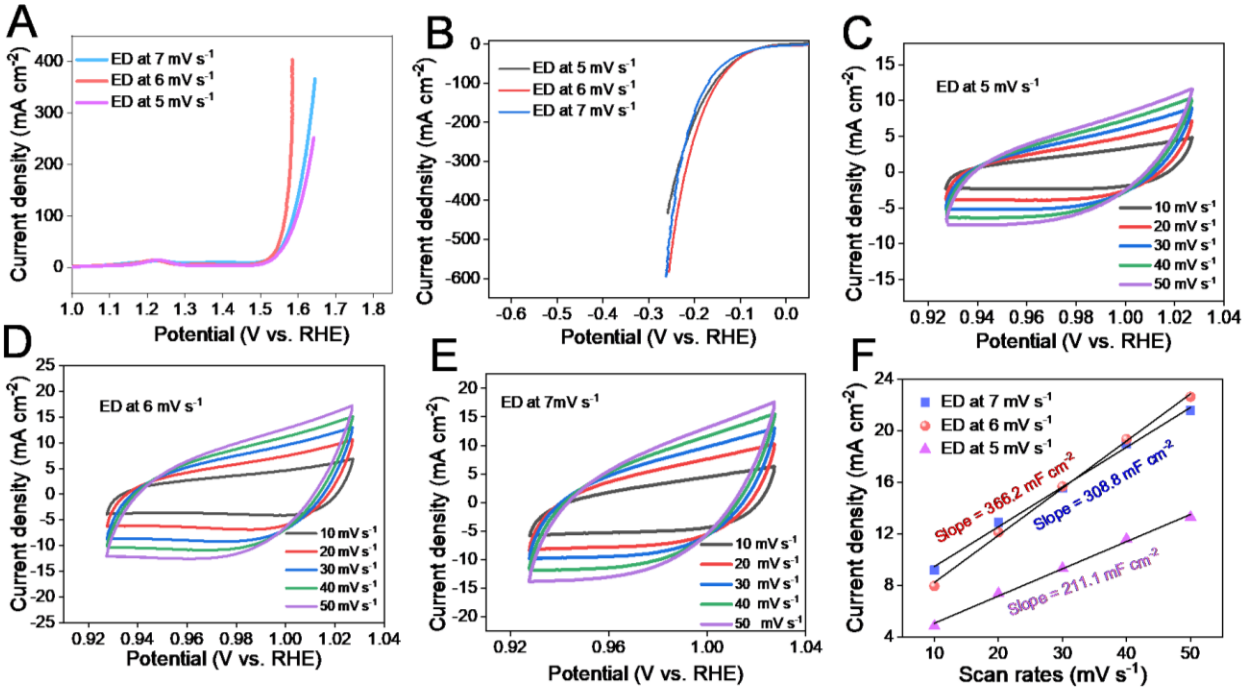

Figure S8. Electrochemical evaluations of the electrodeposited CoNiPO$_x$@V$_{3\%}$-Co$_4$N/NF electrocatalysts prepared at different electrodeposition conditions: (A) LSV for OER, (B) LSV for HER, (C-E) CV profiles evaluated in the non-faradaic region at scan rates from 10-50 mV s^{-1} and (F) scan rate vs. current density plot of the electrodeposited CoNiPO$_x$@V$_{3\%}$-Co$_4$N/NF core-shell heterostructures prepared at different electrodeposition (ED) conditions at scan rates of 5 mV s^{-1} (C), 6 mV s^{-1} (D) and 7 mV s^{-1} (E) respectively for 2 segments.

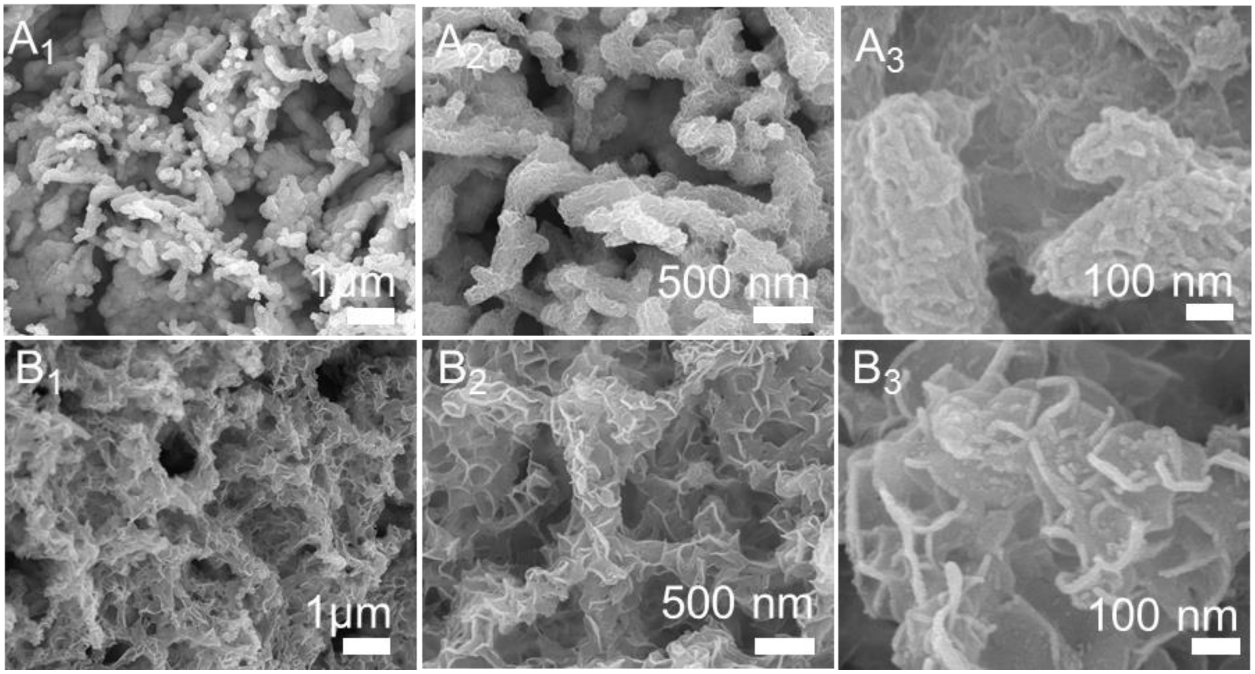

Figure S9. (A_1-A_2, B_1-B_2) Low- and (A_3, B_3) high-magnification FE-SEM images of $NiPO_x$ and $CoPO_x$ nanosheets electrodeposited at the scan rate of 6 mV s^{-1} for 2 segments.

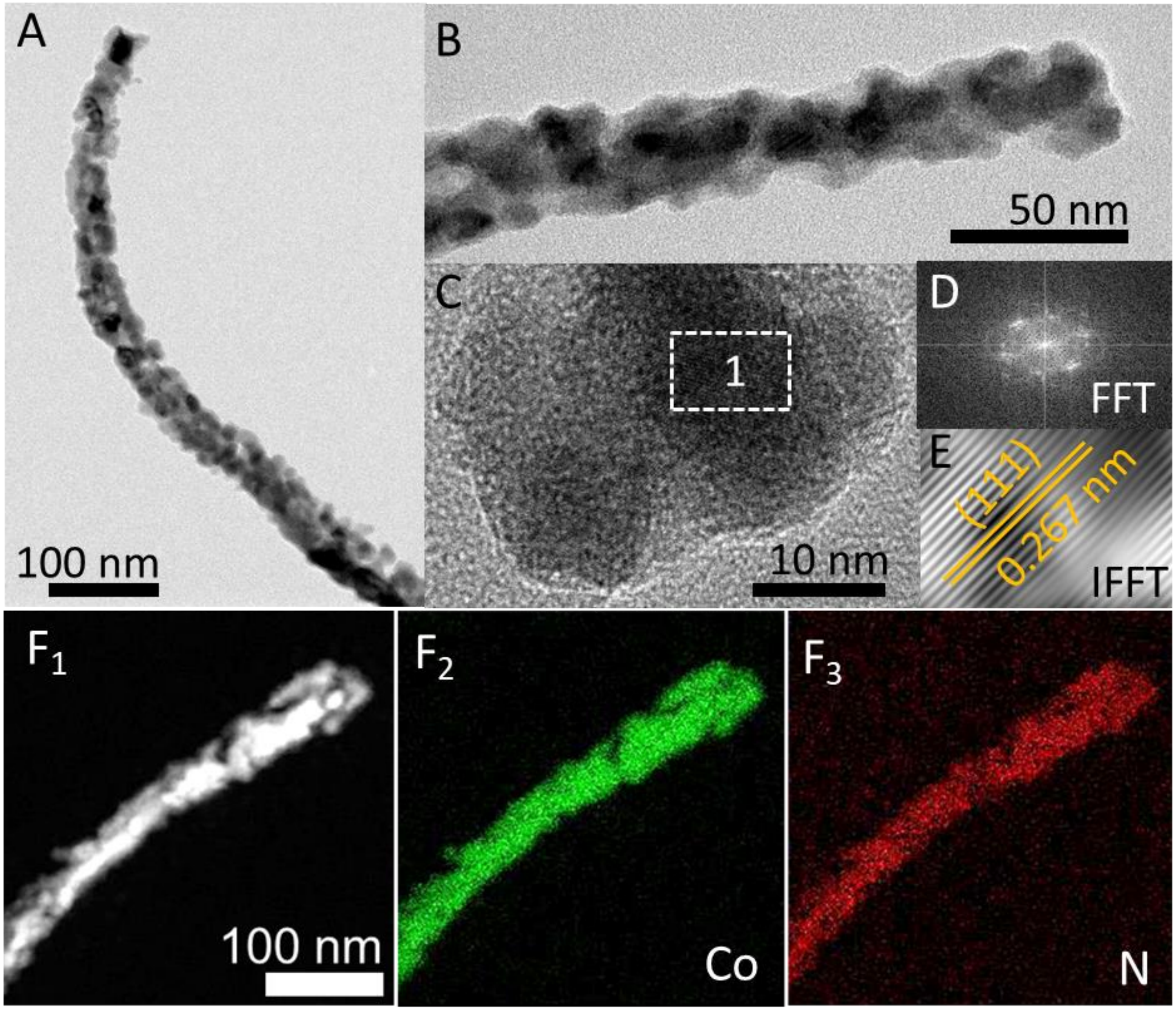

Figure S10. (A-B) Low- and (C) high-resolution TEM images, (D) FFT and (E) IFFT images corresponding to marked region 1 of (C), (F_1) HAADF-STEM image, and elemental color mapping showing the spatial distribution of (F_2) Co, and (F_3) N respectively for Co_4N nanowire. The d spacing of 0.267 nm matched well with the (111) plane of Co_4N.

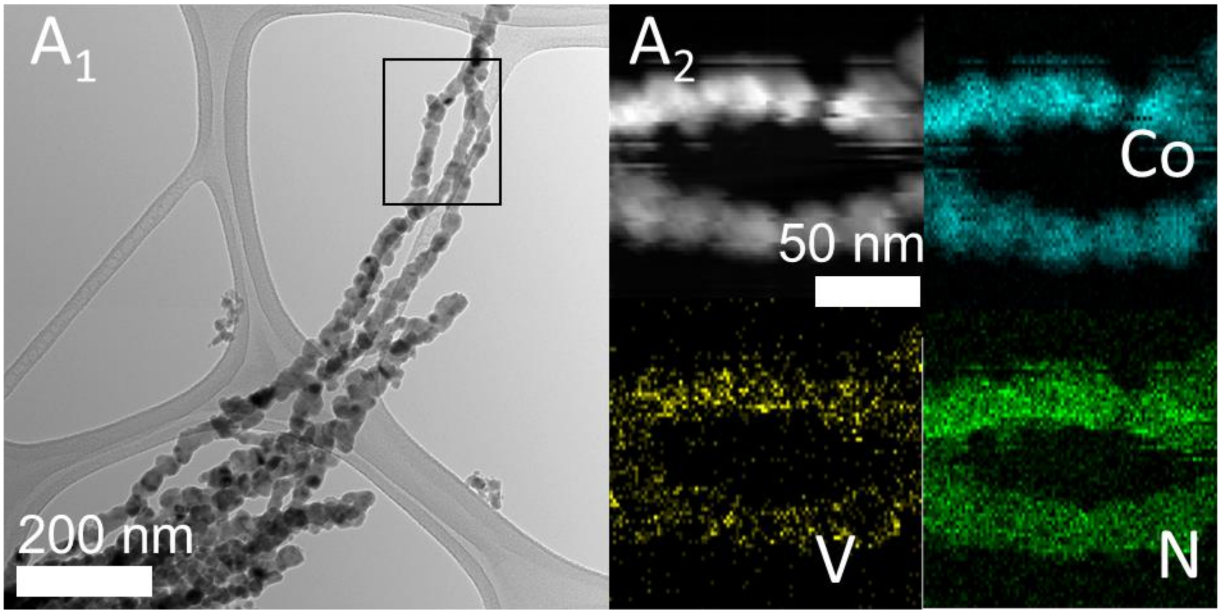

Figure S11. (A_1) Low-resolution TEM image, and (A_2) HAADF-STEM image and elemental color mapping showing the spatial distribution of Co, V, and N respectively for $V_{3\%}$-Co_4N nanowires.

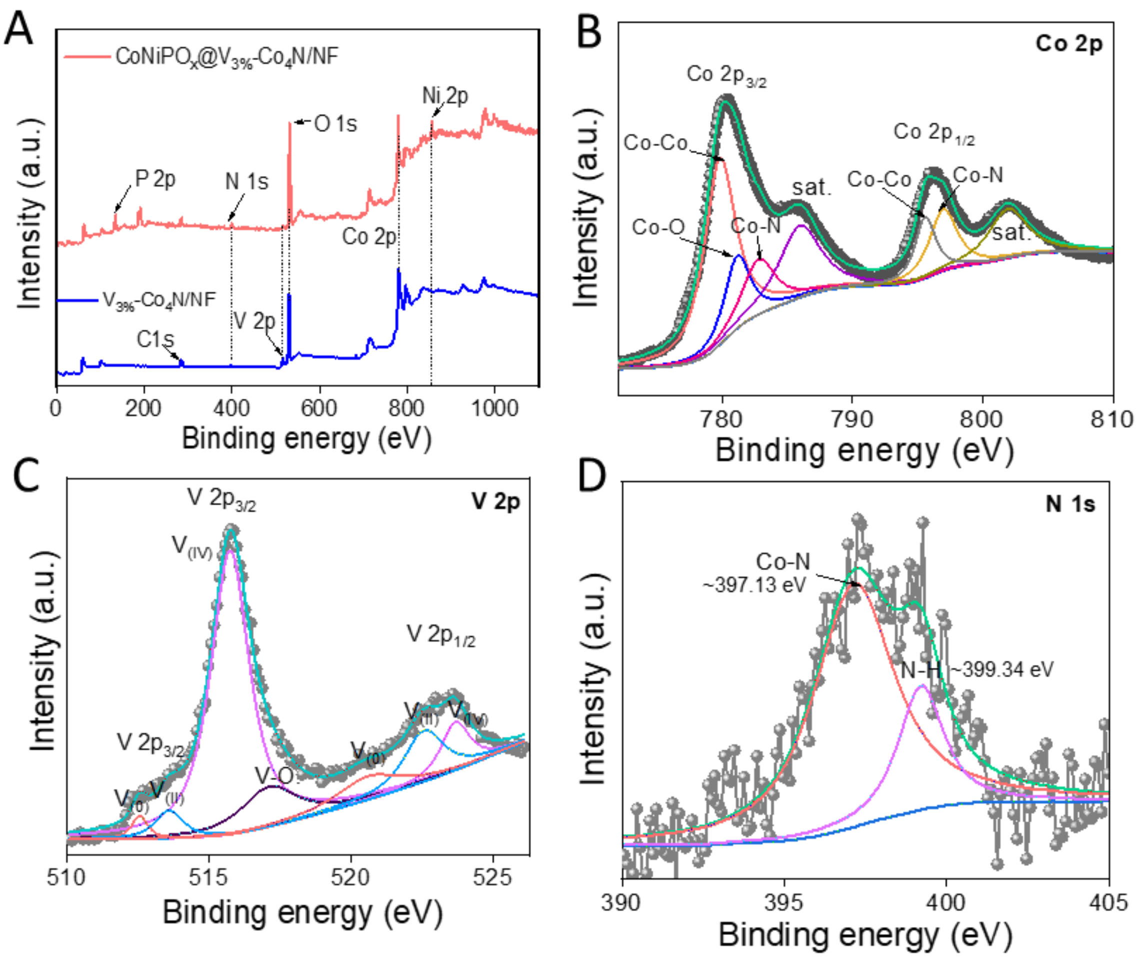

Figure S12. XPS analysis: (A) survey spectra of CoNiPO$_x$@V$_{3\%}$-Co$_4$N and V$_{3\%}$-Co$_4$N and high-resolution core XPS spectra of (B) Co 2p, (C) V 2p, and (D) N 1s of V$_{3\%}$-Co$_4$N core material respectively.

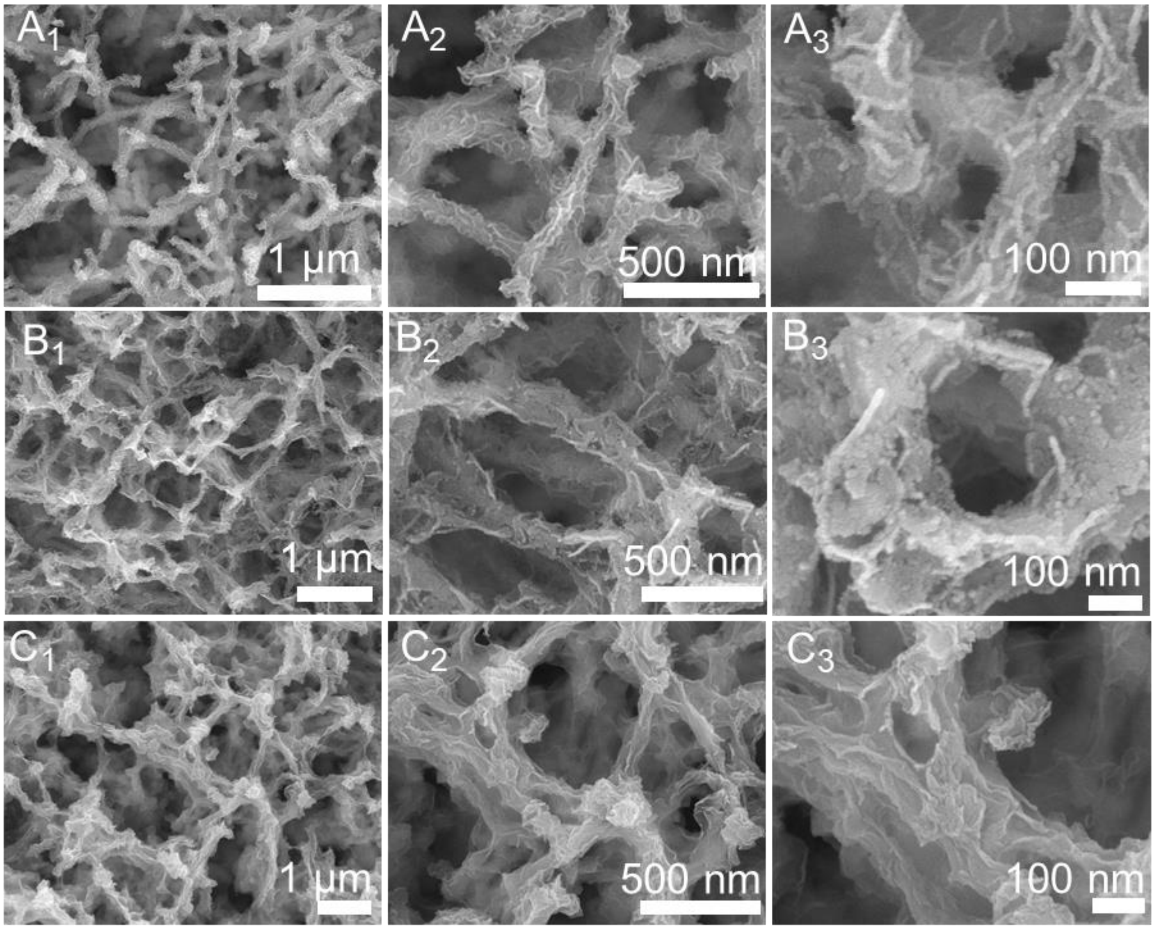

Figure S13. (A_1-A_2, B_1-B_2, & C_1-C_2) Low- and (A_3, B_3, & C_3) high-magnification FE-SEM images of (*C*)-$CoNiPO_x$@$V_{3\%}$-Co_4N/NF, (*C*)-$NiPO_x$@$V_{3\%}$-Co_4N/NF, and (*C*)-$CoPO_x$@$V_{3\%}$-Co_4N/NF crystalline-shell@crystalline-core heterostructures.

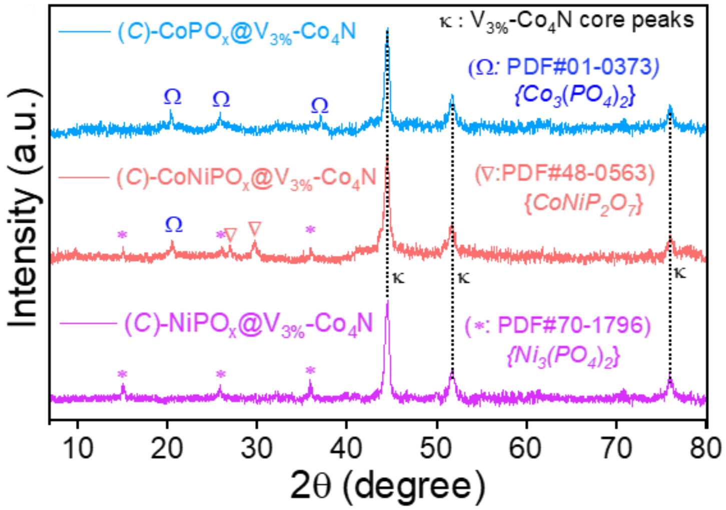

Figure S14. P-XRD patterns of (*C*)-CoNiPO$_x$@V$_{3\%}$-Co$_4$N/NF, (*C*)-NiPO$_x$@V$_{3\%}$-Co$_4$N/NF, and (*C*)-CoPO$_x$@V$_{3\%}$-Co$_4$N/NF crystalline-shell@crystalline-core heterostructures.

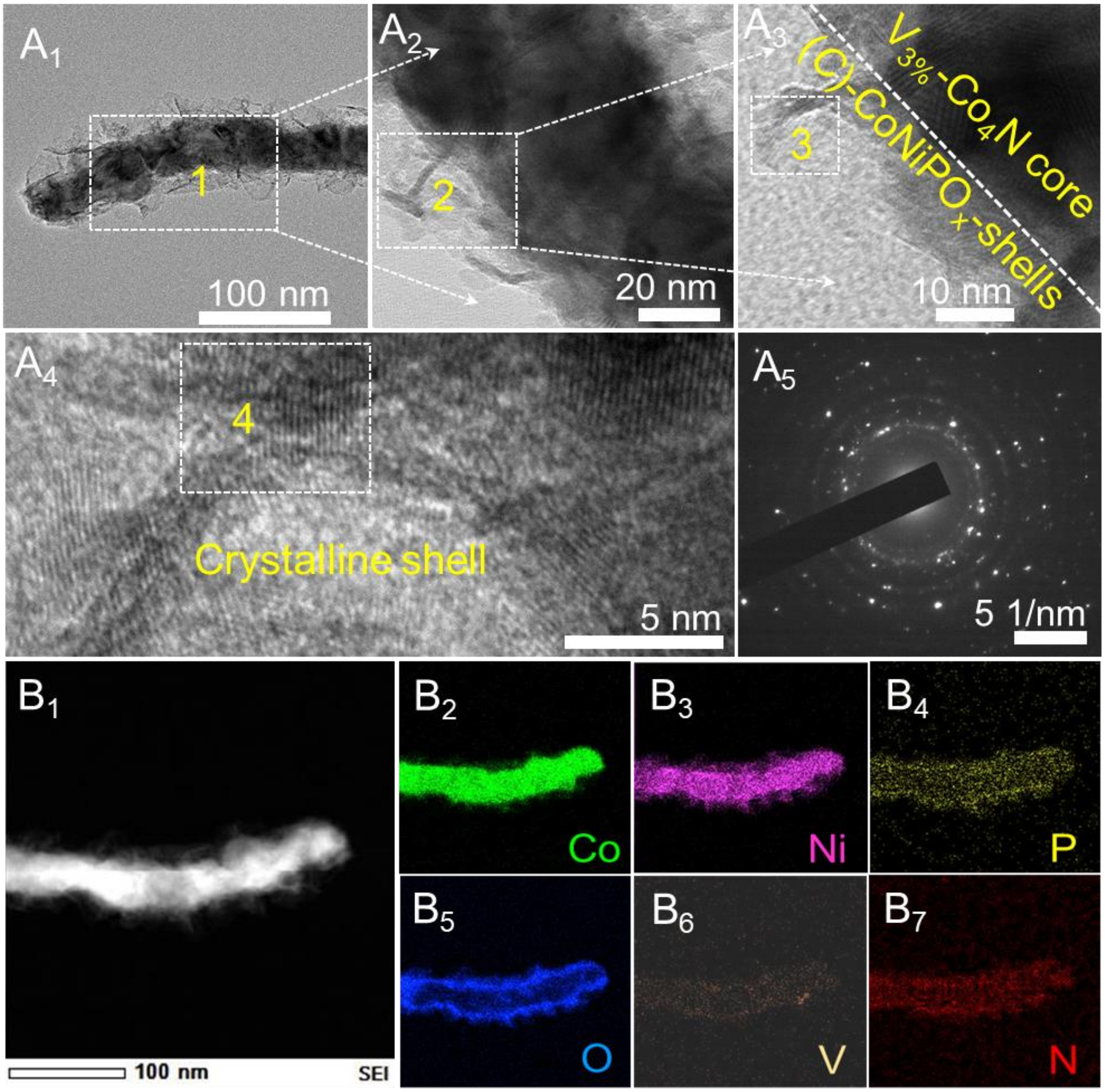

Figure S15. (A_1-A_3) Low-resolution, (A_4) high-resolution TEM image (A_5) SAED image corresponding to shell region 3 of A_3, (B_1) HAADF-STEM image, elemental color mapping showing the presence of (B_2) Co, (B_3) Ni, (B_4) P, (B_5) O, (B_6) V, and (B_7) N respectively for (*C*)-$CoNiPO_x$@$V_{3\%}$-Co_4N/NF.

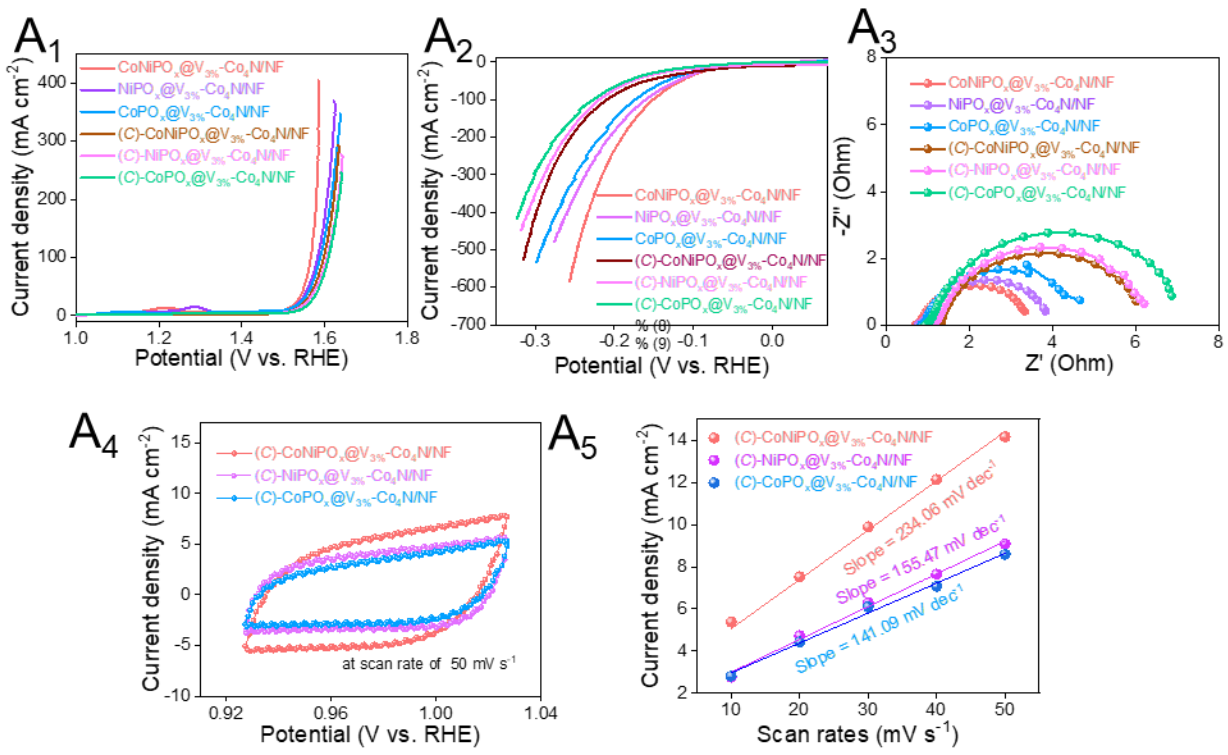

Figure S16. (A_1-A_2 & A_3) Comparison of the OER and HER LSV profiles at scan rate of 2 mV s^{-1} and EIS spectra of amorphous-shell@crystalline-core and crystalline-shell@crystalline-core heterostructures of $CoNiPO_x$@$V_{3\%}$-Co_4N/NF, $NiPO_x$@$V_{3\%}$-Co_4N/NF, and $CoPO_x$@$V_{3\%}$-Co_4N/NF, (A_4) CV profiles at scan rate of 50 mV s^{-1} in the non-faradaic region. Crystalline-shell@crsytalline-core heterostructures are denoted as (*C*)-$CoNiPO_x$@$V_{3\%}$-Co_4N/NF, (*C*)-$NiPO_x$@$V_{3\%}$-Co_4N/NF, and (*C*)-$CoPO_x$@$V_{3\%}$-Co_4N/NF respectively.

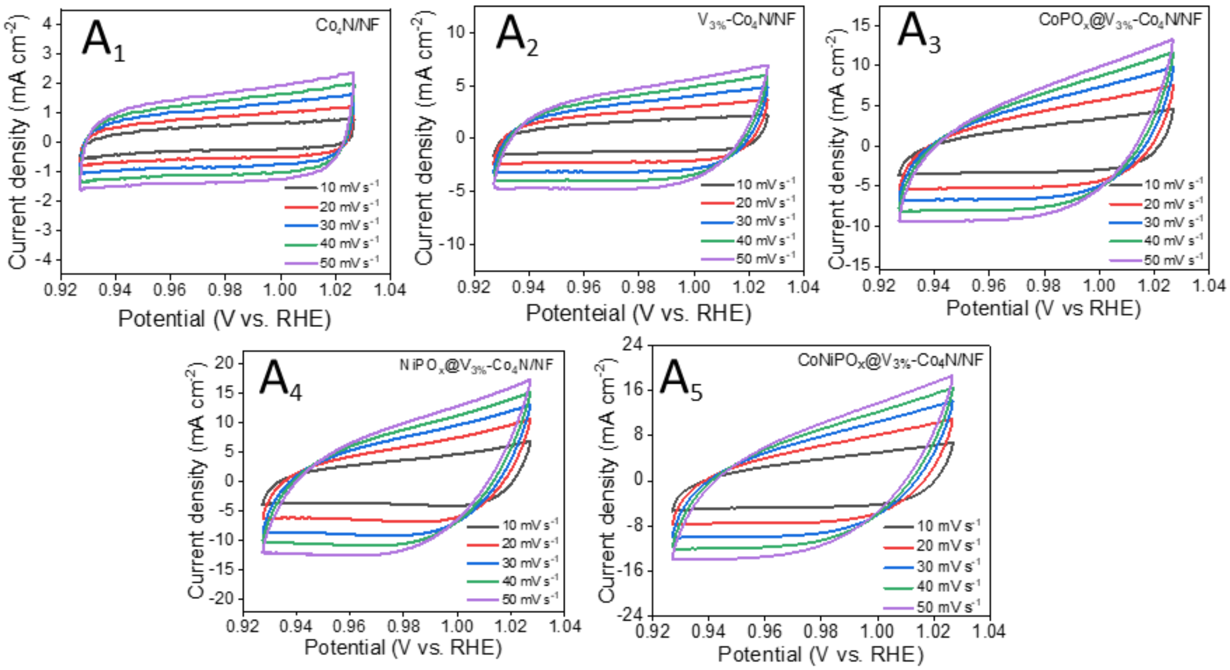

Figure S17. CV profiles at the scan rates of 10-50 mV s^{-1} in the non-faradaic region for (A_1) Co_4N/NF, (A_2) $V_{3\%}$-Co_4N/NF, (A_3) $CoPO_x$@$V_{3\%}$-Co_4N/NF, (A_4) $NiPO_x$@$V_{3\%}$-Co_4N/NF and (A_5) $CoNiPO_x$@$V_{3\%}$-Co_4N/NF electrocatalysts respectively.

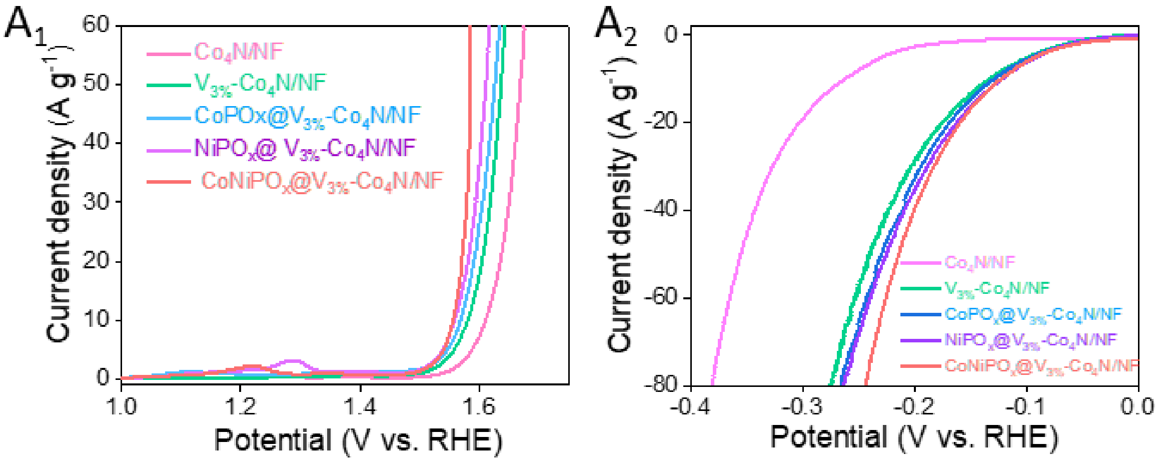

Figure S18. Mass normalized (A_1) OER LSV profiles and (A_2) HER LSV profiles corresponding to the geometric surface area normalized OER and HER LSV profiles of Co_4N/NF, $V_{3\%}$-Co_4N/NF, $CoPO_x$@$V_{3\%}$-Co_4N/NF, $NiPO_x$@$V_{3\%}$-Co_4N/NF, and $CoNiPO_x$@$V_{3\%}$-Co_4N/NF electrocatalysts respectively.

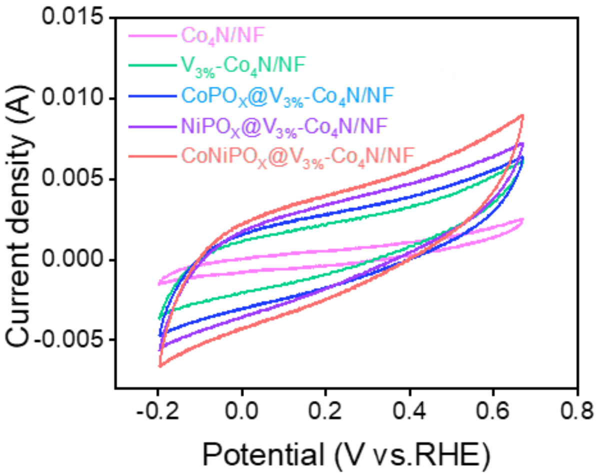

Figure S19. CV profiles of $CoNiPO_x$@$V_{3\%}$-Co_4N/NF, $NiPO_x$@$V_{3\%}$-Co_4N/NF, $CoPO_x$@$V_{3\%}$-Co_4N/NF, $V_{3\%}$-Co_4N/NF, and Co_4N/NF in PBS solution (pH = 7.4) at a scan rate of 50 mV s^{-1} for evaluating the number of active sites using equation (**S_1**).

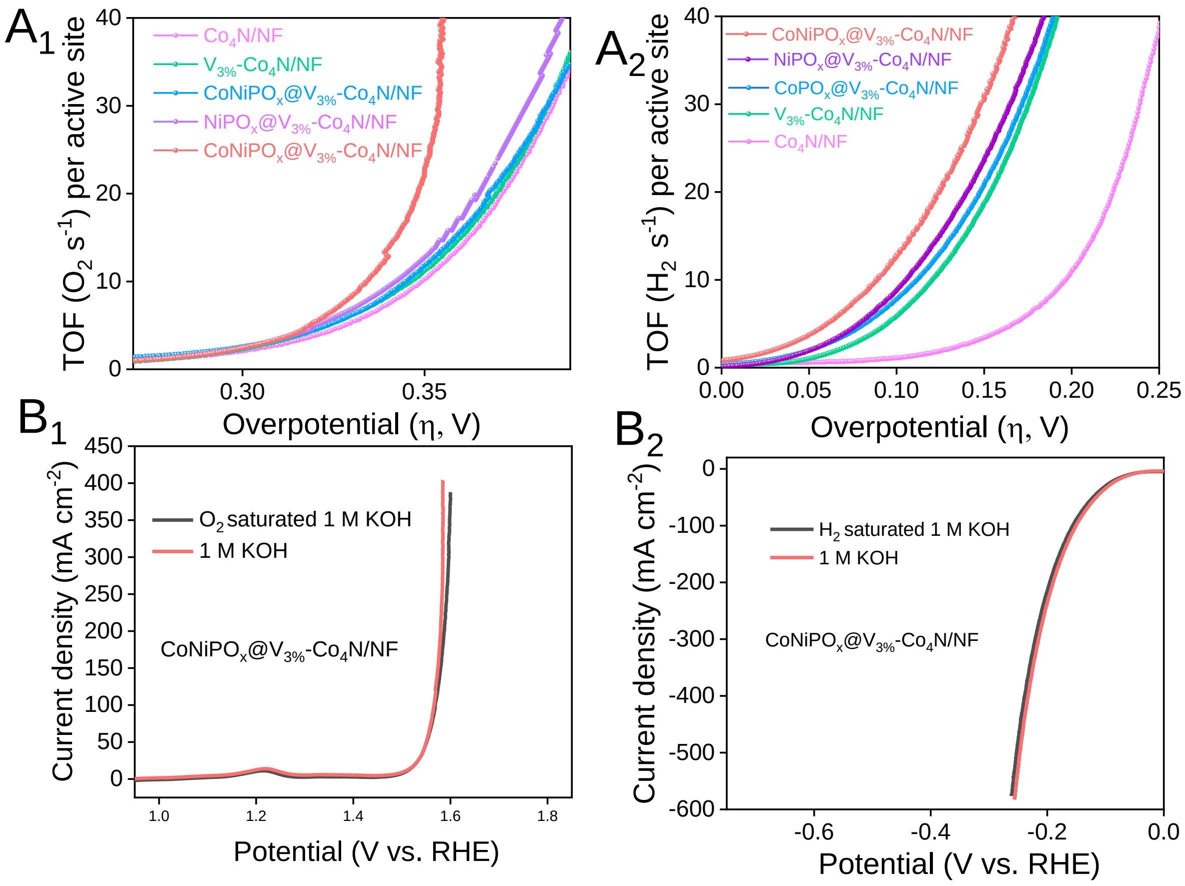

Figure S20. (A_1) Calculated O_2 TOF and (A_2) H_2 TOF of CoNiPO$_x$@V$_{3\%}$-Co$_4$N/NF, NiPO$_x$@V$_{3\%}$-Co$_4$N/NF, CoPO $_x$@V$_{3\%}$-Co$_4$N/NF, V$_{3\%}$-Co$_4$N/NF, and Co$_4$N/NF electrocatalysts, (B_1) OER LSV profile of CoNiPO$_x$@V$_{3\%}$-Co$_4$N/NF recorded at the scan rate of 2 mV s^{-1} using O_2 saturated 1 M KOH electrolyte, and (B_2) HER LSV profile of CoNiPO$_x$@V$_{3\%}$-Co$_4$N/NF recorded at the scan rate of 2 mV s^{-1} using H_2 saturated 1 M KOH electrolyte.

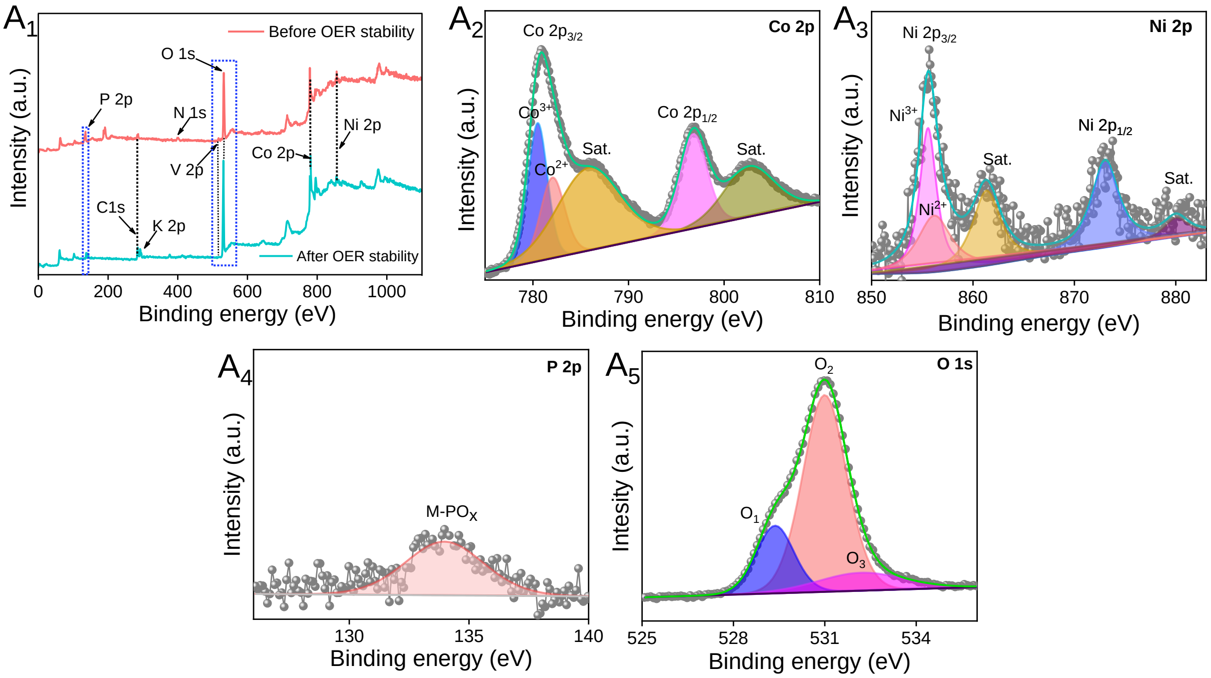

Figure S21. (A_1) XPS survey spectrum of $CoNiPO_x$@$V_{3\%}$-Co_4N electrocatalysts after the long-term CP stability test for OER compared to pre-stability test, and its high-resolution XPS core-spectra of (A_2) Co 2p, (A_3) Ni 2p, (A_4) P 2p, and (A_5) O 1s respectively after the long-term OER stability test.

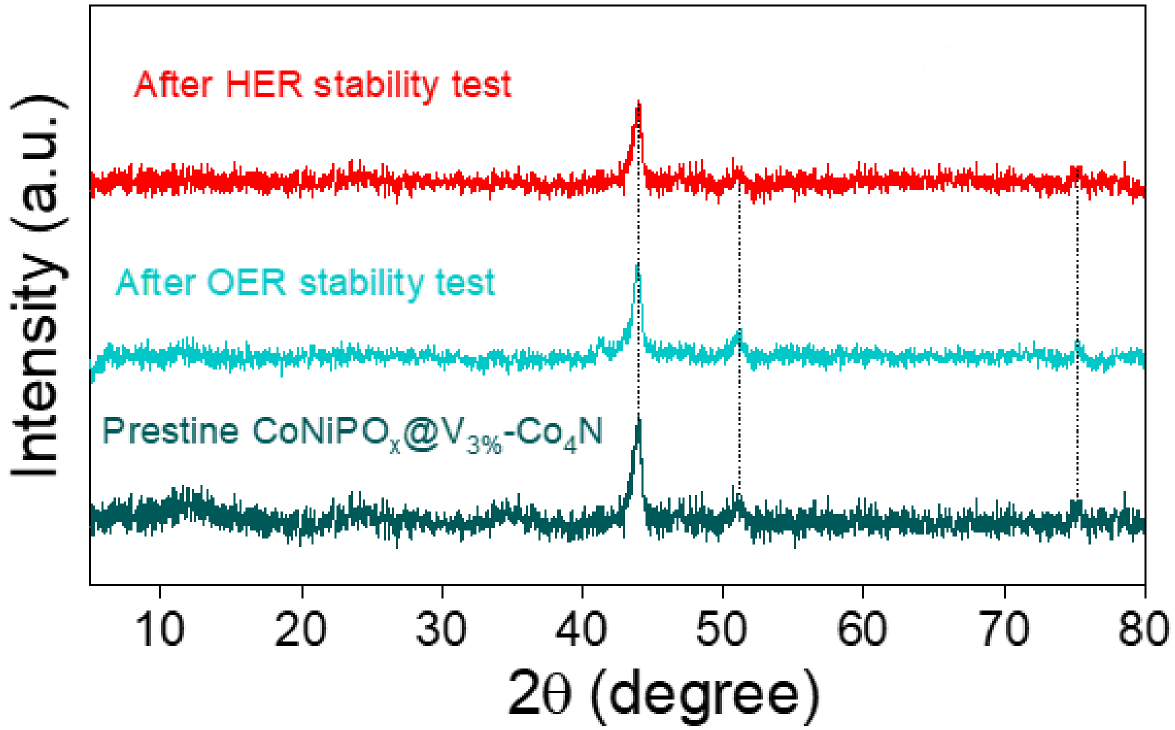

Figure S22. P-XRD patterns of $CoNiPO_x@V_{3\%}-Co_4N$ electrocatalysts after the long-term CP stability test for OER and HER compared to the pre-stability test.

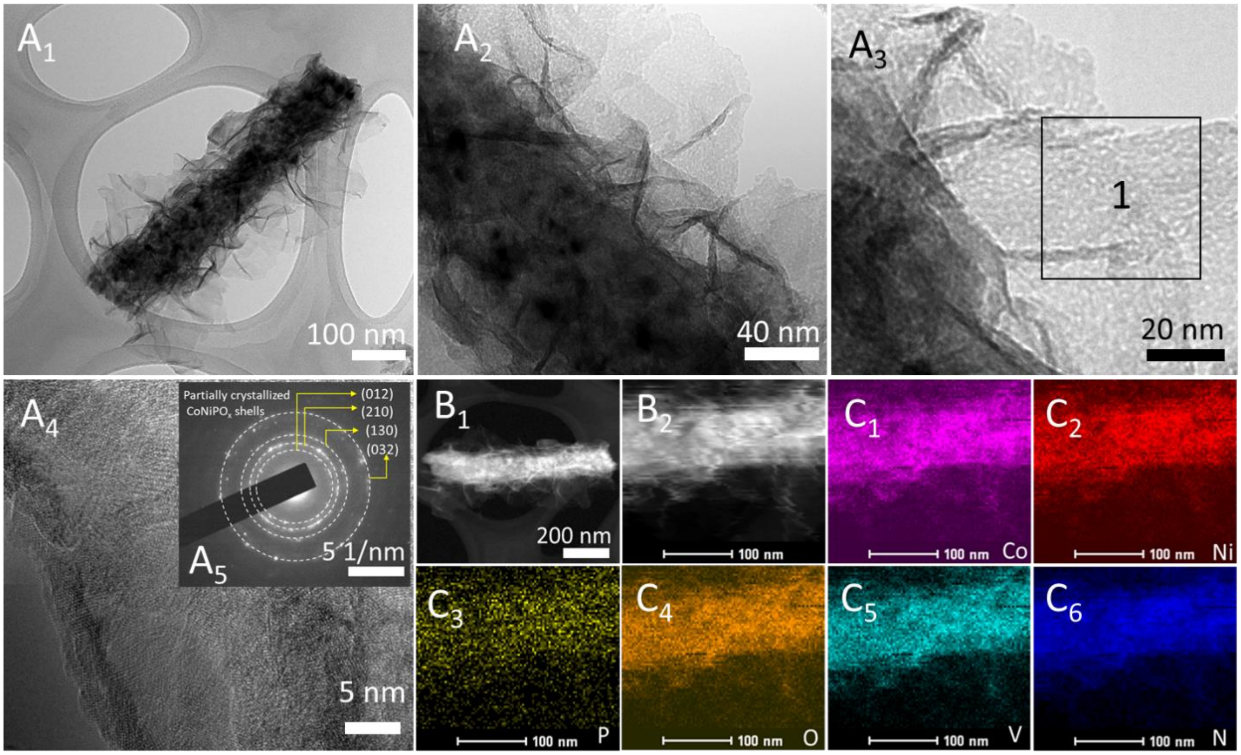

Figure S23. (A_1-A_2) Low-resolution, (A_3-A_4) high-resolution TEM images, (A_5) SAED pattern of region 1 of A_3 showing that the CoNiPOx shell undergoes mild crystallization resulting in diffraction patterns that matched with those of (012), (210), (130) and (032) of $CoNiP_2O_7$ (PDF#48-0563) thus revealing that the diffraction circles belong to $CoNiPO_x$ and not CoNi-hydroxides, (B_1-B_2) low-and high-resolution HAADF-STEM images, and elemental color mapping indicating the spatial distribution of (C_1) Co, (C_2) Ni, (C_3) P, (C_4) O, (C_5) V and (C_6) N respectively for $CoNiPO_x@V_{3\%}$-Co_4N/NF after the long-term CP stability test for OER.

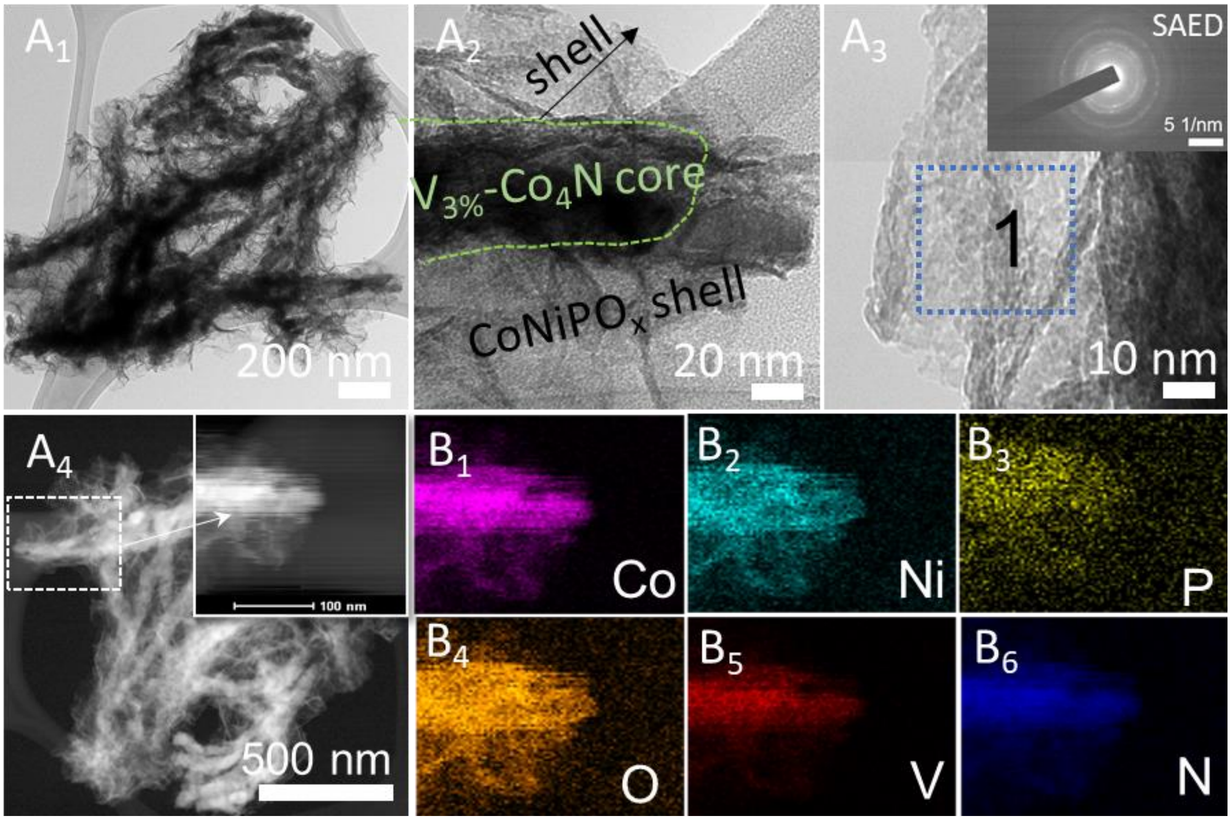

Figure S24. (A_1-A_2) Low-resolution, (A_3 & inset) high-resolution TEM images and SAED pattern of region 1 of A_3, HAADF-STEM image (A_4 & inset), and elemental color mapping showing the presence of (B_1) Co, (B_2) Ni, (B_3) P, (B_4) O, (B_5) V and (B_6) N respectively for $CoNiPO_x@V_{3\%}$-Co_4N heterostructure after long-term stability test for HER.

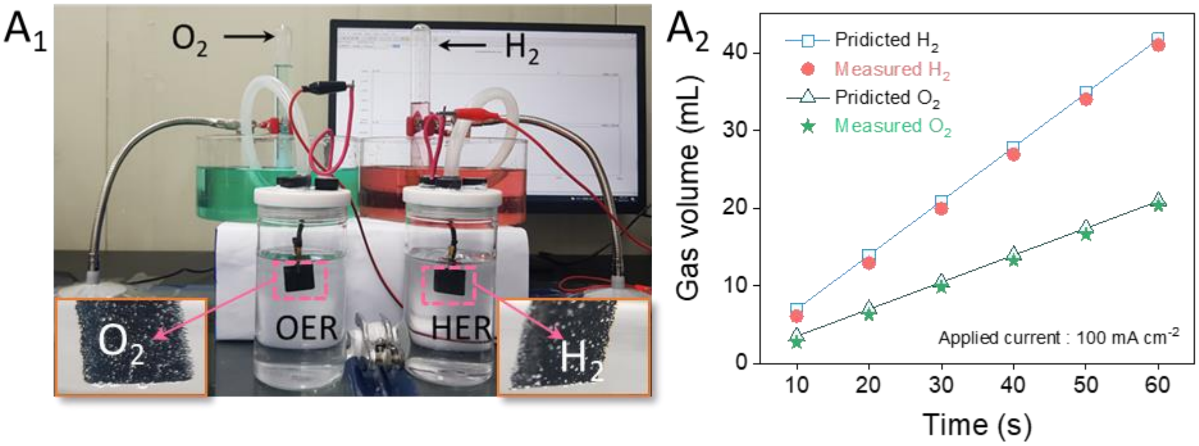

Figure S25. (A_1) Digital photograph of the lab customized experimental setup for the collection of the evolved O_2 and H_2 gas using the inverted graded measuring cylinders, (A_2) comparison of theoretically predicted and experimentally obtained quantity of H_2 and O_2 gas at an applied current density of 100 mA cm^{-2} for 60 min for amorphous-shell@crystaliine-core based $CoNiPO_x$@$V_{3\%}$-Co_4N/NF (+/-) alkaline electrolyzer.

Table S1. Comparison of electrocatalytic oxygen evolution reaction (OER) performance of our prepared electrocatalysts with recent reports.

SL no.	Electrocatalysts	Electrolyte	Overpotential@ Current density	Tafel slope (mV dec^{-1})	Ref.
**1**	**$CoNiPO_x$@$V_{3\%}$-Co_4N/NF**<br>**$NiPO_x$@$V_{3\%}$-Co_4N/NF**<br>**$CoPO_x$@$V_{3\%}$-Co_4N/NF**<br>**$V_{3\%}$-Co_4N/NF**<br>**Co_4N/NF**<br>**RuO_2/NF**	1.0 M KOH	**270 mV@ 10 mA cm^{-2}**<br>**280 mV@ 10 mA cm^{-2}**<br>**283 mV@ 10 mA cm^{-2}**<br>**297 mV@ 10 mA cm^{-2}**<br>**340 mV@ 10 mA cm^{-2}**<br>**326 mV@ 10 mA cm^{-2}**	**54.66**<br>**69.59**<br>**75.17**<br>**78.14**<br>**79.74**<br>**151.18**	**This work**
**2**	Ti/TiN@$Co_{5.47}N$	1.0 M KOH	211 mV @ 10 mA cm^{-2}	42.5	[3]
**3**	EO-Mo-/Co-N-C/Cu	1.0 M KOH	394 mV @ 10 mA cm^{-2}	58	[4]
**4**	(N, S)-RGO@CoN-3	1.0 M Na_2SO_4	220 mV @ 10 mA cm^{-2}	33	[5]
**5**	CoVN/CoFeN	1.0 M KOH	244 mV @ 50 mA cm^{-2}	34.8	[6]
**6**	$Co_{0.5}(V_{0.5})$	1.0 M KOH	282 mV @ 10 mA cm^{-2}	56	[7]
**7**	Co_2P@Co/N-C/GC	1.0 M KOH	320 mV @ 10 mA cm^{-2}	48.8	[8]
**8**	CoN- 1min	1.0 M KOH	290 mV @ 10 mA cm^{-2}	70	[9]
**9**	exfoliated NiCo-LDH	1.0 M KOH	330 mV @ 10 mA cm^{-2}	41	[10]
**10**	Ni_3FeN/N-G	1.0 M KOH	320 mV @ 10 mA cm^{-2}	38	[11]
**11**	Ni-Co-P HNBs/NF	1.0 M KOH	270 mV @ 10 mA cm^{-2}	76	[12]
**12**	CoVFeN@NF	1.0 M KOH	212 mV @ 10 mA cm^{-2}	34.8	[6]
**13**	$Ni_{1.5}Sn$@triMPO_4-R	1.0 M KOH	240 mV @ 10 mA cm^{-2}	45.2	[13]
**14**	Ni_2P–CoP/GC	1.0 M KOH	320 mV @ 10 mA cm^{-2}	69	[14]
**15**	DR-Ni_3FeN/N-G	1.0 M KOH	250 mV @ 10 mA cm^{-2}	38	[11]
**16**	Co_4N-CeO_2/GP	1.0 M KOH	239 mV @ 10 mA cm^{-2}	37.1	[15]
**17**	Ni-doped Co_4N	1.0 M KOH	233 mV @ 10 mA cm^{-2}	61	[16]
**18**	CoN-F	1.0 M KOH	276.2 mV @ 10 mA cm^{-2}	52.3	[17]
**19**	Co_4N@NC	1.0 M KOH	257 mV @ 10 mA cm^{-2}	58	[18]
**20**	$Co_{5.47}N$ NP@N-PC	1.0 M KOH	248 mV @ 10 mA cm^{-2}	72	[19]

Table S2. Comparison of electrocatalytic hydrogen evolution reaction (HER) performance of our prepared electrocatalysts with recent reports.

SL no.	Electrocatalysts	Electrolyte	Overpotential @10 mA cm^{-2}	Tafel slope (mV dec^{-1})	Ref.
**1**	**$V_{3\%}$-Co_4N@CoNiPO$_x$/NF**<br>**NiPO$_x$@$V_{3\%}$-Co_4N/NF**<br>**CoPO$_x$@$V_{3\%}$-Co_4N/NF**<br>**$V_{3\%}$-Co_4N/NF**<br>**Co_4N/NF**<br>**20% Pt/C/NF**	1.0 M KOH	**53 mV**<br>**58 mV**<br>**64 mV**<br>**69 mV**<br>**205 mV**<br>**15 mV**	**85.7**<br>**107.3**<br>**158.3**<br>**162.1**<br>**171.2**<br>**40.3**	**This work**
**2**	Ni_3FeN	1.0 M KOH	238 mV	46	[20]
**3**	V-Ni_3S_2/Ni_xP_y/NF	1.0 M KOH	90 mV	94.9	[21]
**4**	$NiCo_3N$	1.0 M KOH	96 mV	-NA-	[22]
**5**	FeN-500	1.0 M KOH	171 mV	96	[23]
**6**	Ru-SAs@N-TC	1.0 M KOH	159 mV	37.5	[24]
**7**	$Co_{1-x}Fe_x$-LDH/NF	1.0 M KOH	205 mV		[25]
**8**	Fe-Ni@NC-CNTs /GC	1.0 M KOH	202 mV	113	[26]
**9**	Co/WN NWs	1.0 M KOH	151 mV	82	[27]
**10**	CoP@PS/NCNT	1.0 M KOH	80 mV	53	[28]
**11**	S-M-1Pt	1.0 M KOH	104 mV	98	[29]
**12**	Mo_5N_6	1.0 M KOH	94 mV	-NA-	[30]
**13**	$Cu_{0.3}Co_{2.7}P$/NC	1.0 M KOH	220 mV	129	[31]
**14**	V-$Ti_4N_3T_x$	0.5 M H_2SO_4	330 mV	107	[32]
**15**	VN-Co-P	1.0 M KOH	137 mV	81	[33]
**16**	V-doped Co_4N nanosheets	1.0 M KOH	37 mV	44	[34]
**17**	Co_4N-CeO_2/GP	1.0 M KOH	24 mV	61	[15]
**18**	Co_4N–CeO_2/NF	1.0 M KOH	52 mV	56.8	[35]
**19**	CoN-F	1.0 M KOH	191.7 mV	129.9	[17]
**20**	CoN NF/NF	1.0 M KOH	129 mV	108	[36]

Table S3. Comparison of electrocatalytic overall alkaline water splitting performance of our prepared electrolyzers with recent reports.

SL no.	Anode materials	Cathode materials	Cell potential@ 10 mA cm^{-2}	Electrolyte	Ref.
**1**	**$CoNiPO_{x@}V_{3\%}$-Co_4N/NF**<br>**$NiPO_{x@}V_{3\%}$-Co_4N/NF**<br>**$CoPO_{x@}V_{3\%}$-Co_4N/NF**<br>**$V_{3\%}$-Co_4N/NF**<br>**Co_4N/NF**<br>**RuO_2/NF**	**$CoNiPO_{x@}V_{3\%}$-Co_4N/NF**<br>**$NiPO_{x@}V_{3\%}$-Co_4N/NF**<br>**$CoPO_{x@}V_{3\%}$-Co_4N/NF**<br>**$V_{3\%}$-Co_4N/NF**<br>**Co_4N/NF**<br>**20%-Pt/C/NF**	**1.52 V**<br>**1.57 V**<br>**1.59 V**<br>**1.62 V**<br>**1.67 V**<br>**1.55 V**	1.0 M KOH	**This work**
**2**	Ni-Co-P HNBs/NF	Ni-Co-P HNBs/NF	1.62 V	1.0 M KOH	[12]
**3**	VN-Co-P	VN-Co-P	1.70 V	1.0 M KOH	[33]
**4**	$Co_{0.75}Ni_{0.25}(OH)_2$	$Co_{0.75}Ni_{0.25}(OH)_2$	1.56 V	1.0 M KOH	[37]
**5**	Ni_3FeN-NPs	Ni_3FeN-NPs	1.81 V	1.0 M KOH	[20]
**6**	EO Mo-/Co-N-C/Cu	EO Mo-/Co-N-C/Cu	1.62 V	1.0 M KOH	[4]
**7**	$Fe_xCo_{2-x}P$/NF	$Fe_xCo_{2-x}P$/NF	1.61 V	1.0 M KOH	[38]
**8**	Fe-Ni_2P/MoS_x/NF	Fe-Ni_2P/MoS_x/NF	1.61 V	1.0 M KOH	[39]
**9**	$Ni_{1.85}Fe_{0.15}P$ NSAs/NF	$Ni_{1.85}Fe_{0.15}P$ NSAs/NF	1.61 V	1.0 M KOH	[40]
**10**	P-NM-C HNRs	P-NM-C HNRs	1.63 V	1.0 M KOH	[41]
**11**	CoP/NCNHP/GC	CoP/NCNHP/GC	1.64 V	1.0 M KOH	[42]
**12**	Co_6W_6C@NC/CC	Co_6W_6C@NC/CC	1.58 V	1.0 M KOH	[43]
**13**	NC-CNT/CoOOH/CC	NC-CNT/CoOOH/CC	1.63 V	1.0 M KOH	[44]
**14**	$(Co_{1-x}Ni_x)(S_{1-y}P_y)_2$/graphene/GC	$(Co_{1-x}Ni_x)(S_{1-y}P_y)_2$/graphene/GC	1.65 V	1.0 M KOH	[45]
**15**	$NiCo_2S_4$/NF	$NiCo_2S_4$/NF	1.61 V	1.0 M KOH	[46]
**16**	Nb–Co_4N/NF	Nb–Co_4N/NF	1.61 V	1.0 M KOH	[47]
**17**	CoN-F	CoN-F	1.613 V	1.0 M KOH	[17]
**18**	Co_4N-CeO_2/GP	Co_4N-CeO_2/GP	1.507 V	1.0 M KOH	[15]
**19**	Co_4N@NC-700	Co_4N@NC-700	1.561 V	1.0 M KOH	[18]
**20**	$Co_{5.47}N$ NP@N-PC	$Co_{5.47}N$ NP@N-PC	1.62 V	1.0 M KOH	[19]

Calculation of the number of active sites:

For the determination of the number of active sites in the prepared electrocatalysts, the method reported by Tian et al. was referred.[48,49] Typically, CV profiles of the electrocatalysts were recorded at a scan rate of 50 mV s^{-1} in PBS solution (pH =7.4) as shown in **Figure S19, SI**. Then, the absolute value of the voltammetric charge (both anodic and cathodic) was evaluated by integrating the area under the curve. Then, the number of active sites (*n*) is then calculated using the equation (**S_1**)[50,51]

$$n = \frac{Q}{2F} = \frac{It}{2F} = \frac{IV}{2F\nu} \qquad (\mathbf{S_1})$$

where Q is the total voltammetric charge and F is the faraday constant (C mol^{-1}), I is the current (A), t is the time (s) and V is the potential (volt, V) and ν is the scan rate (V s^{-1}).

Calculation of turnover frequency (TOF):

After determining the number of active sites of the electrocatalysts, the turnover frequency for OER and HER was determined using equation (**S_2**), [49][51]

TOF = *jA/nmF* (**S_2**)

Where, *j* = current in ampere (A) obtained during LSV measurement,

A = area of the electrode (1 cm^{-2}),

F = Faraday constant (96485 C mol^{-1}),

m = Number of electrons transferred to generate one molecule of the product (i.e., *m* = 4 for OER and *m* = 2 for HER) and

n = Number of active sites catalyzing the reactions respectively (mol^{-1}).

Measurement of Faradic efficiency:

For evaluating Faradaic efficiency, the gas evolved was measured based on the water displacement method using inverted graded measuring cylinders in 1 M KOH at an applied current density of 100 mA cm^{-2} for 1 h using lab assembled set-up as shown in **Figure S25A$_1$, SI**. For comparison, the theoretically predicted gas volume of H_2 and O_2 gas were also estimated using the following equations, similar to previous reports:[51–53]

$$V_{H2} (mL) = \{Q (C) \times 22.4 (L mol^{-1}) \times 1000\} / \{2F(C mol^{-1})\} \quad (S3)$$

$$V_{O2} (mL) = \{Q (C) \times 22.4 (L mol^{-1}) \times 1000\} / \{4F(C mol^{-1})\} \quad (S4)$$

where Q is the cumulative charge (C) and F is the Faraday constant (C mol^{-1}).

Finally, Faradaic efficiency is calculated as, $FE (\%) = \frac{\text{Measured gas volume}}{\text{Pridicted gas volume}} \times 100$ (S5)

Computational details:

Density functional theory (DFT) calculations have been performed using the Perdew-Burke-Ernzerhof (PBE) functional with a generalized gradient approximation (GGA) as implemented in VASP.5.4.4.[54,55] A plane-wave basis with a kinetic energy cut-off of 300 eV has been chosen to represent the electrons of all systems. The Co_4N (111) core and its vanadium doped analogues, along with $Co_3(PO_4)_2$, $Ni_3(PO_4)_2$, $CoNiPO_x$ shells have been sampled at a Monkhorst-pack scheme of 3×3×1; whereas the $CoPO_x$@$V_{3\%}$-Co_4N, $NiPO_x$@$V_{3\%}$-Co_4N and $CoNiPO_x$@$V_{3\%}$-Co_4N core-shell heterostructures have been sampled with 2×1×1 k-points scheme. The contribution from long-range van der Waals interlayer interactions has also been accounted by incorporating the DFT-D3 method.[56] The geometry relaxation of all systems has been carried out till the electronic energies difference and atomic forces converge to 10-6 eV and 0.05 eV/Å, respectively.

The optimized systems are then exposed to the water molecule and their water adsorption efficacy (ΔE_{H2O}) has been calculated according to the formula: $\Delta E_{H_2O} = E_{system+H_2O} - E_{system} - E_{H_2O}$, wherein $E_{system+H_2O}$ and E_{system}, respectively, is the total electronic energy of the system with and without water adsorption. The Gibbs free energy change (ΔG) for HER and OER

have been calculated with the equation: ΔG = ΔE + ΔZPE - TΔS, wherein, ΔE is the electronic energy difference between two consecutive intermediates, ΔZPE and TΔS are the change in zero-point energy and change in entropy at T=298.15 K obtained from vibrational frequency calculation.

The key reaction steps of HER in an alkaline medium and the Gibbs free energies to be calculated are:

(1) system (*) + H_2O + e^- + → H* + OH^- (Volmer)

(2) H* + H_2O + e^- → H_2 + OH^- (Heyrovsky)

Or,

(3) system (*) + H_2O + e^- → H* + OH^- (Volmer)

(4) H* + H* → H_2 (Tafel)

$$\Delta G_{H-0} = G* + G_{H_2O}$$

$$\Delta G_{H-1} = G_{OH-H*}$$

$$\Delta G_{H-2} = G_{H*} + G_{OH-}$$

$$\Delta G_{H-3} = \Delta G_0 = G* + G_{OH-} + G_{H_2O}$$

Similarly, the key reaction steps of 4-electron OER in alkaline medium and the Gibbs free energy equations are:

(1) system (*) + OH^- → *OH + e^-

(2) *OH + OH^- → *O + H_2O + e^-

(3) *O + OH^- → *OOH + e^-

(4) *OOH + OH^- → *O_2 + e^- + H_2O

(5) *O_2 → * + O_2

$$\Delta G_{O-1} = G_{OH*}$$

$$\Delta G_{O-2} = G_{O*} - G_{OH*}$$

$$\Delta G_{O-3} = G_{OOH*} - G_{O*}$$

$$\Delta G_{O-4} = G_{O_2*} - G_{OOH*}$$

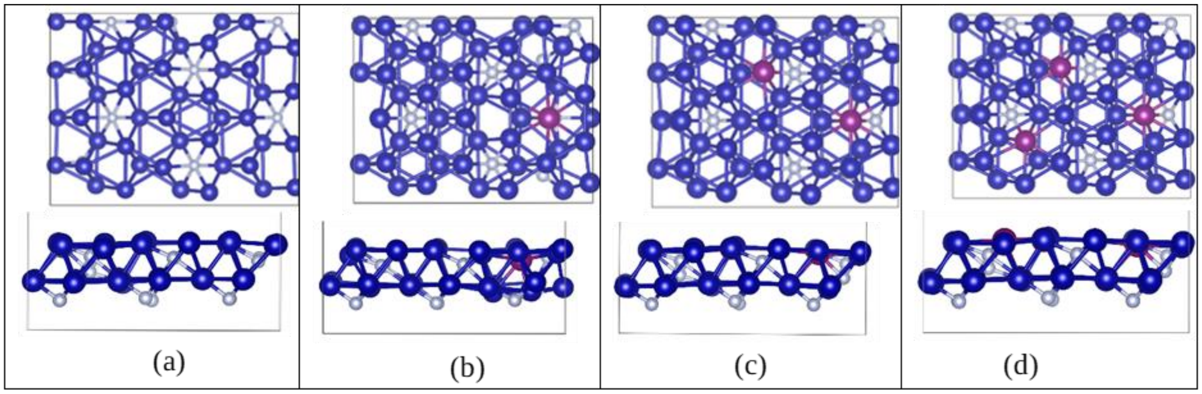

Figure S26: Top and side view models of core (a) Co_4N (b) $V_{1\%}$-Co_4N (c) $V_{3\%}$-Co_4N and (d) $V_{5\%}$-Co_4N respectively.

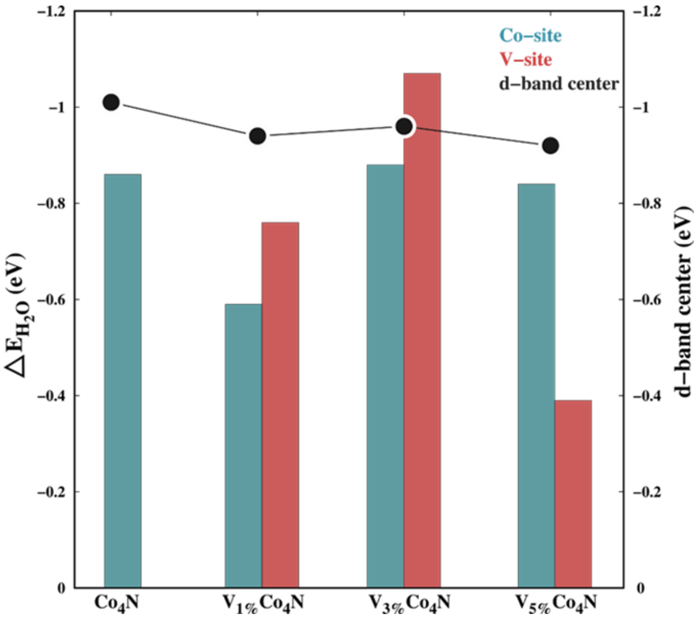

Figure S27: Adsorption energy of water, ΔE_{H2O} on Co and V-sites of Co_4N, $V_{1\%}$-Co_4N, $V_{3\%}$-Co_4N, and $V_{5\%}$-Co_4N along with information of their d-band centers.

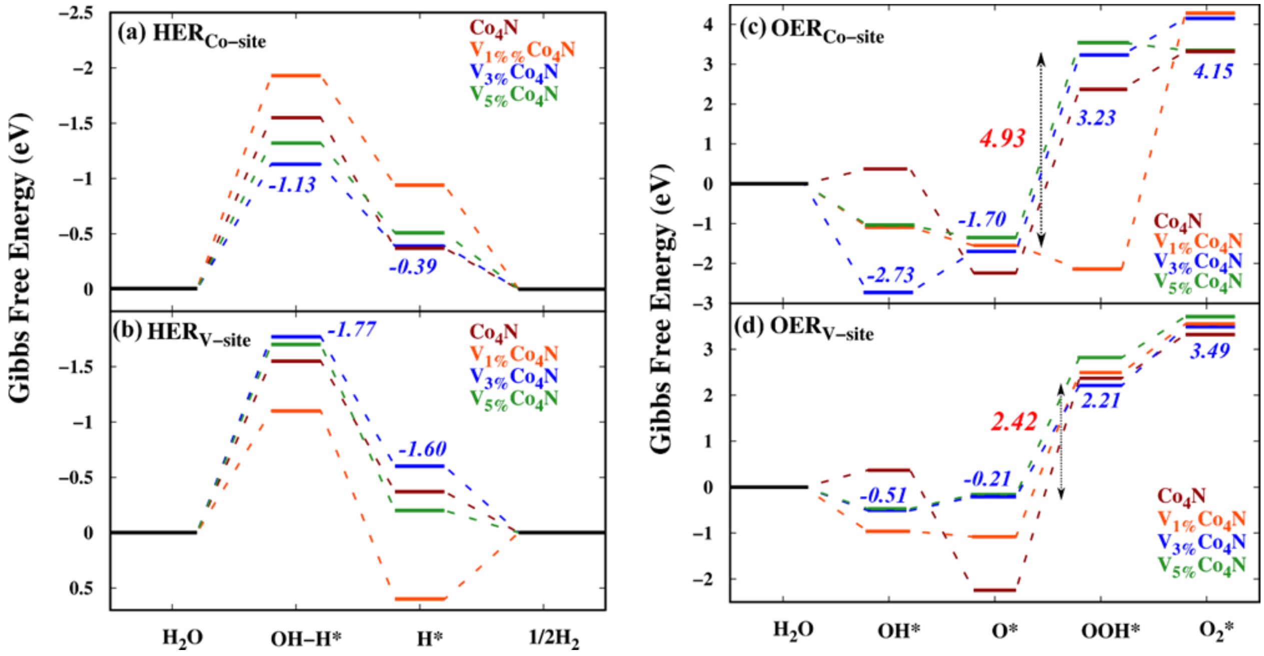

Figure S28: Free energy diagram of HER and OER on Co and V-sites in alkaline condition. The Gibbs free energy of $V_{3\%}$-Co_4N has been highlighted to show HER is favored on Co-site while OER is preferred on V-site in alkaline condition.

Table S4: Gibbs free energy of intermediate steps on Co and V-sites in alkaline HER and OER in Co_4N and $V_{x\%}$-Co_4N core.

Gibbs fee energy	Co-site				V-site			
	Co_4N	$V_{1\%}$-Co_4N	$V_{3\%}$-Co_4N	$V_{5\%}$-Co_4N	Co_4N	$V_{1\%}$-Co_4N	$V_{3\%}$-Co_4N	$V_{5\%}$-Co_4N
ΔG_{max} HER	1.55	1.93	**1.13**	1.32	-	1.10	1.77	1.70
ΔG_{max} OER	4.61	6.42	4.93	4.90	-	3.57	**2.42**	2.97
OH-H*	-1.55	-1.93	-1.32	-1.32	-	-1.10	-1.77	-1.70
H*	-0.37	-0.94	-0.39	-0.51	-	0.60	-0.60	-0.20
OH*	0.37	-1.09	-2.73	-1.05	-	-0.96	-0.51	-0.47
O*	-2.24	-1.55	-1.70	-1.35	-	-1.08	-0.21	-0.16
OOH*	2.37	-2.14	3.23	3.54	-	2.49	2.21	2.82
O_2*	3.32	4.28	4.15	3.35	-	3.55	3.49	3.71

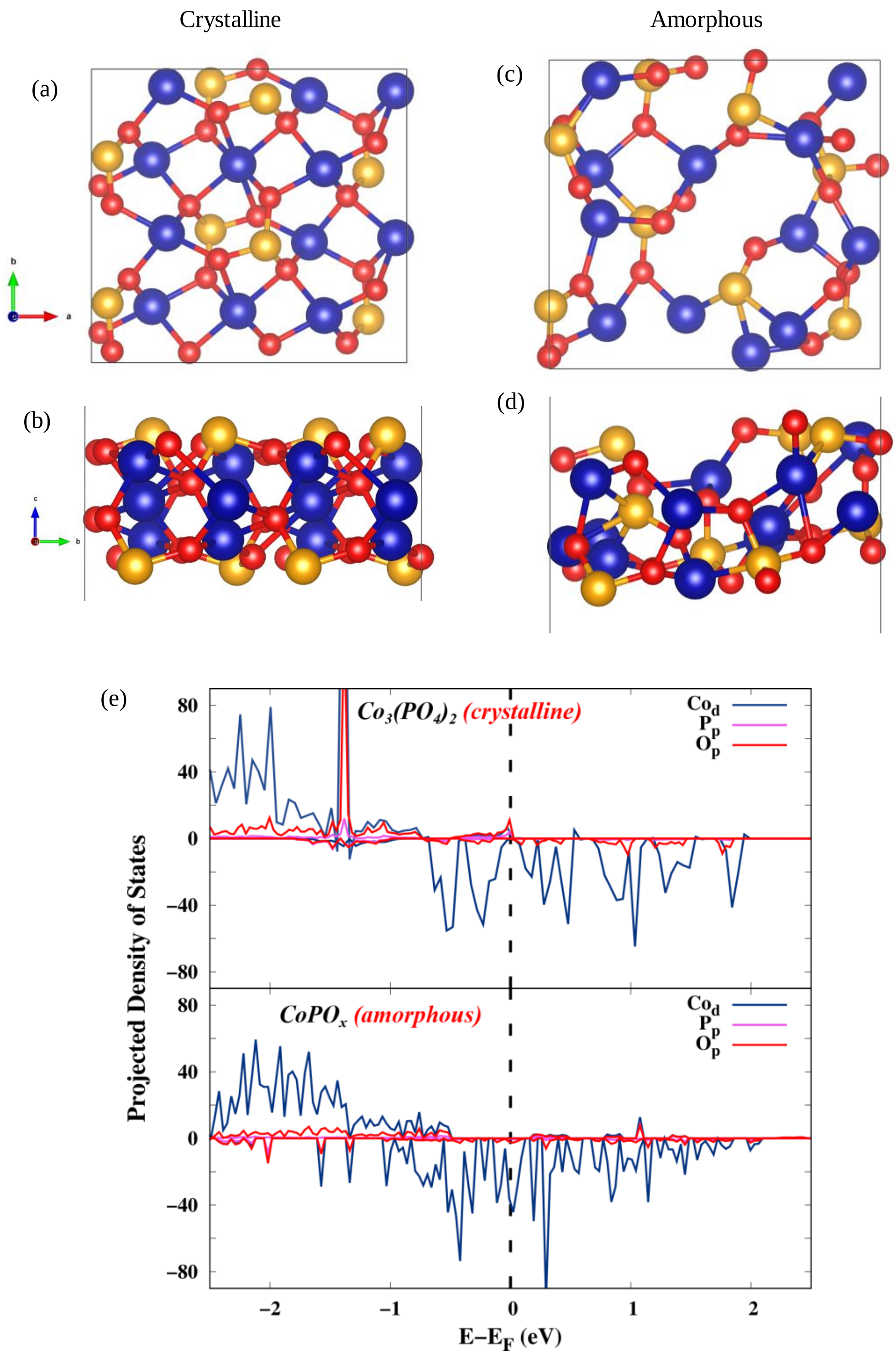

Figure S29: Models of (a, b) crystalline $Co_3(PO_4)_2$ and (c,d) amorphous $CoPO_x$, and (e) their corresponding projected density of states.

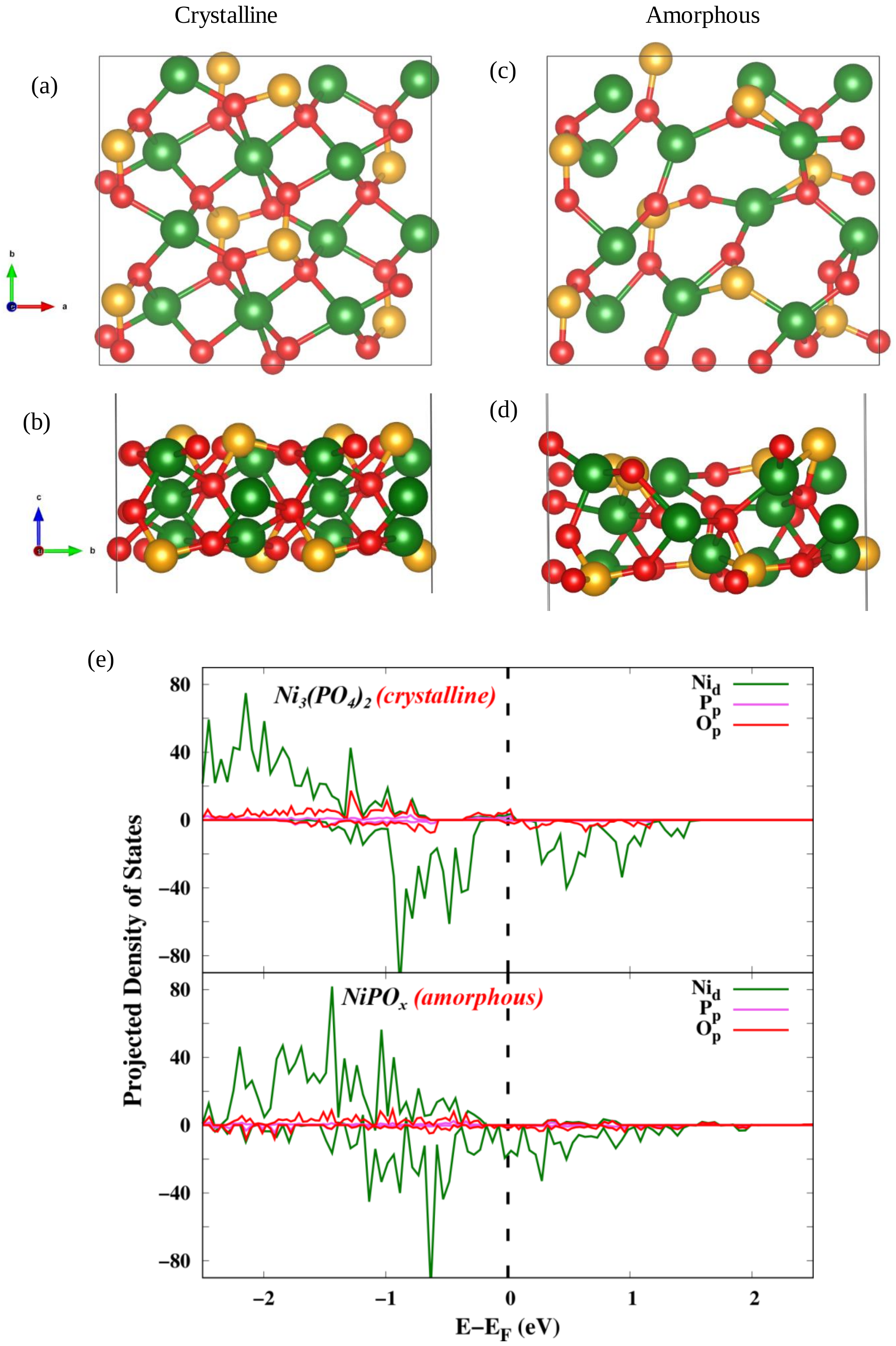

Figure S30: Models of (a, b) crystalline $Ni_3(PO_4)_2$ and (c,d) amorphous $NiPO_x$ and (e) their corresponding projected density of states.

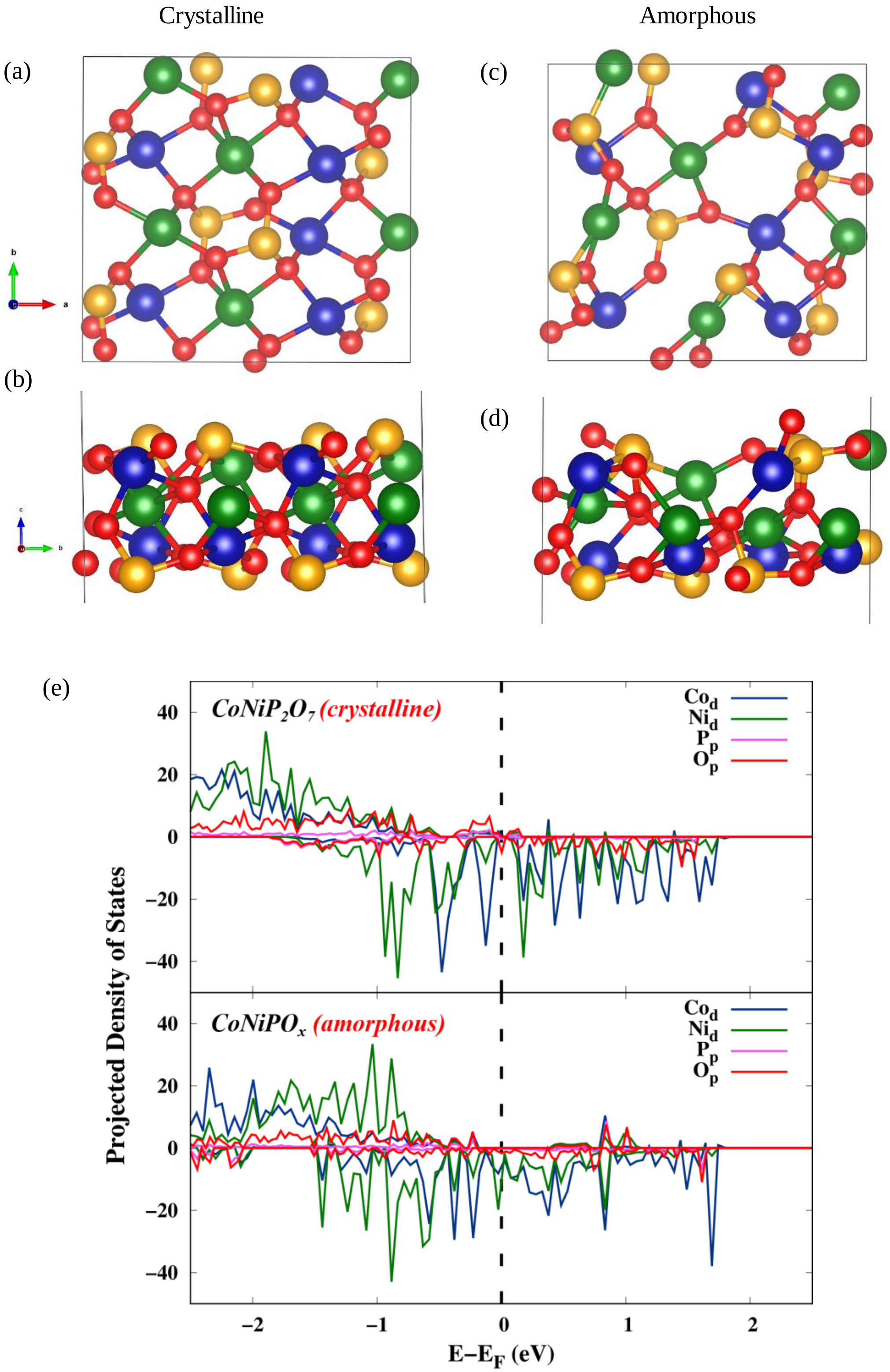

Figure S31: Models of (a, b) crystalline $CoNiP_2O_7$ and (c,d) amorphous $CoNiPO_x$ and (e) their corresponding projected density of states.

(a) (b)

(c) (d)

(e) (f)

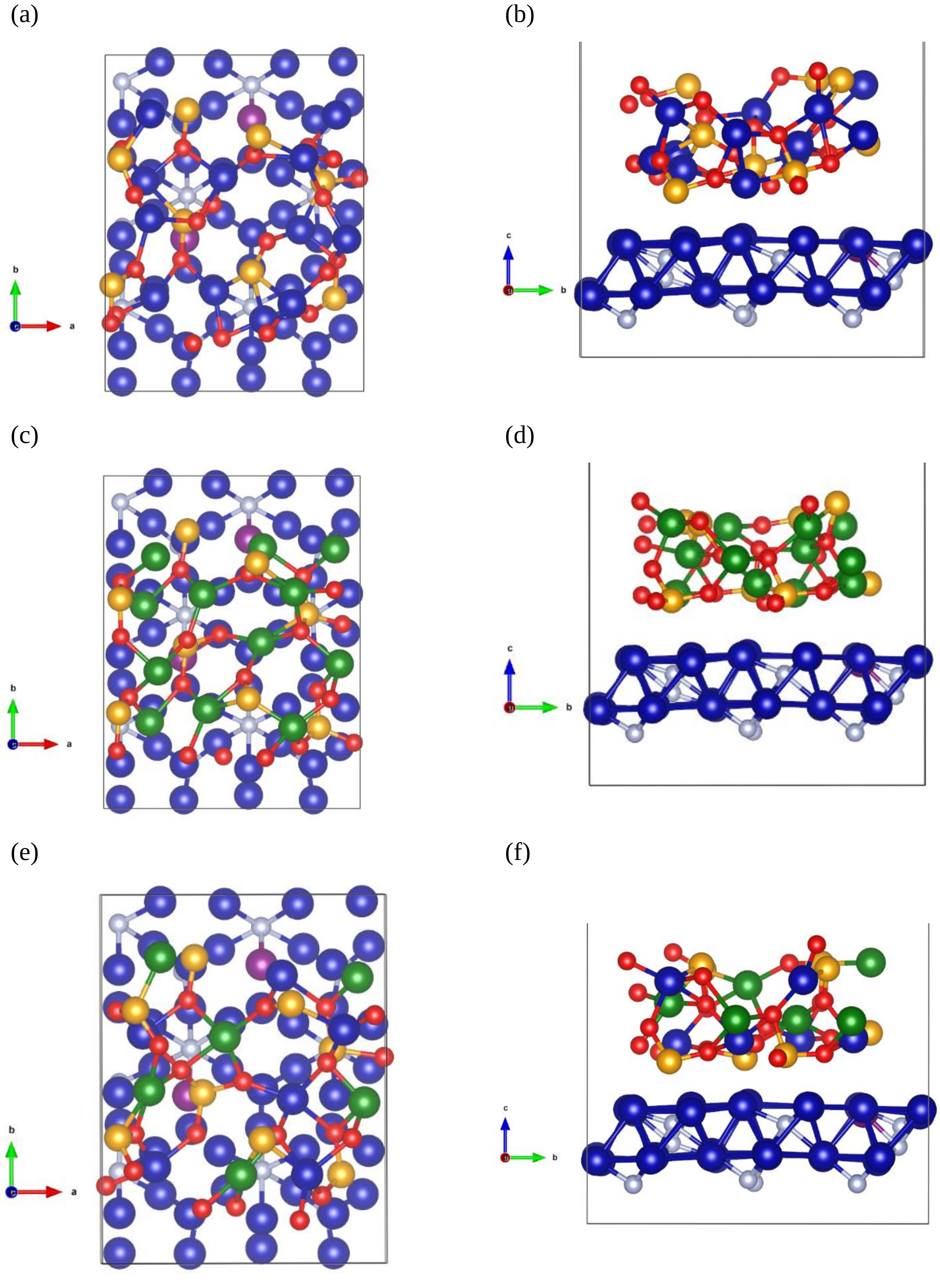

Figure S32: Amorphous-shell@crystalline-core models of (a, b) $CoPO_x@V_{3\%}Co_4N$, (c, d) $NiPO_x@V_{3\%}Co_4N$ and (e, f) $CoNiPO_x@V_{3\%}Co_4N$.

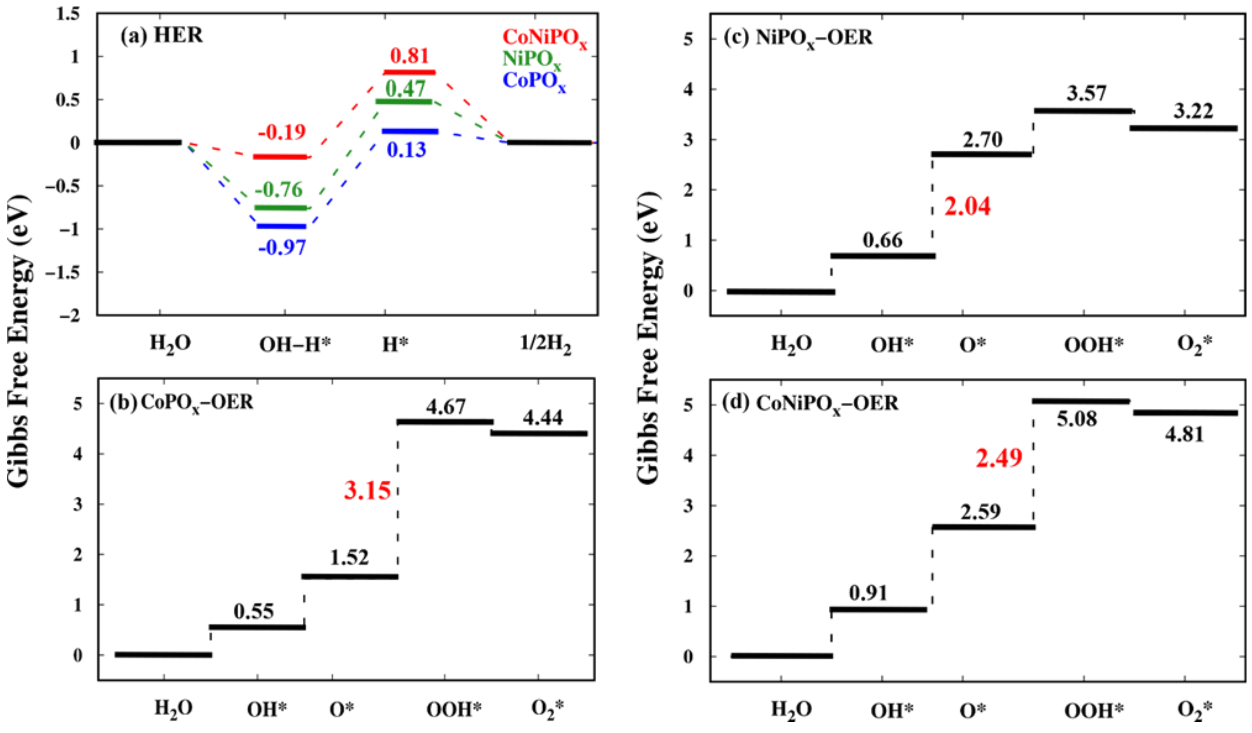

Figure S33: Free energy diagram of HER and OER in alkaline condition on amorphous Cobalt phosphate ($CoPO_x$), Nickel phosphate ($NiPO_x$) and Cobalt-Nickel phosphate ($CoNiPO_x$) shells.

REFERENCE

[1] W. Wang, M. Ma, M. Kong, Y. Yao, N. Wei, *Micro Nano Lett.* **2017**, *12*, 264.

[2] P. Bhojane, A. Le Bail, P. M. Shirage, *Acta Crystallogr. Sect. C Struct. Chem.* **2019**, *75*, 61.

[3] D. Guo, Z. Wan, Y. Li, B. Xi, C. Wang, *Adv. Funct. Mater.* **2021**, *31*, 2008511.

[4] H. Shi, T. Dai, W. Wan, Z. Wen, X. Lang, Q. Jiang, *Adv. Funct. Mater.* **2021**, *31*, 2102285.

[5] D. Guo, Z. Zeng, Z. Wan, Y. Li, B. Xi, C. Wang, *Adv. Funct. Mater.* **2021**, *31*, 2101324.

[6] D. Liu, H. Ai, J. Li, M. Fang, M. Chen, D. Liu, X. Du, P. Zhou, F. Li, K. H. Lo, Y. Tang, S. Chen, L. Wang, G. Xing, H. Pan, *Adv. Energy Mater.* **2020**,

10, 2002464.

[7] K. Fan, H. Zou, L. Duan, L. Sun, *Adv. Energy Mater.* **2020**, *10*, 1903571.

[8] C. Zhu, S. Fu, B. Z. Xu, J. Song, Q. Shi, M. H. Engelhard, X. Li, S. P. Beckman, J. Sun, D. Du, Y. Lin, *Small* **2017**, *13*, 1700796.

[9] Y. Zhang, B. Ouyang, J. Xu, G. Jia, S. Chen, R. S. Rawat, H. J. Fan, *Angew. Chemie - Int. Ed.* **2016**, *55*, 8670.

[10] F. Song, X. Hu, *Nat. Commun.* **2014**, *5*, 1.

[11] S. Zhao, M. Li, M. Han, D. Xu, J. Yang, Y. Lin, N.-E. Shi, Y. Lu, R. Yang, B. Liu, Z. Dai, J. Bao, *Adv. Funct. Mater.* **2018**, *28*, 1706018.

[12] E. Hu, Y. Feng, J. Nai, D. Zhao, Y. Hu, X. W. Lou, *Energy Environ. Sci.* **2018**, *11*, 872.

[13] S. Li, Z. Li, R. Ma, C. Gao, L. Liu, L. Hu, J. Zhu, T. Sun, Y. Tang, D. Liu, J. Wang, *Angew. Chemie* **2021**, *133*, 3817.

[14] X. Liang, B. Zheng, L. Chen, J. Zhang, Z. Zhuang, B. Chen, *ACS Appl. Mater. Interfaces* **2017**, *9*, 23222.

[15] H. Sun, C. Tian, G. Fan, J. Qi, Z. Liu, Z. Yan, F. Cheng, J. Chen, C. P. Li, M. Du, *Adv. Funct. Mater.* **2020**, *30*, 1910596.

[16] D. Li, W. Zhang, J. Zeng, B. Gao, Y. Tang, Q. Gao, *Sci. China Mater.* **2021**, *64*, 1889.

[17] T. Liu, S. Cai, Z. Mei, G. Zhao, L. Xu, Q. An, Y. Fu, H. Wang, M. Li, H. Guo, *Sustain. Energy Fuels* **2021**, *5*, 3632.

[18] W. Yuan, S. Wang, Y. Ma, Y. Qiu, Y. An, L. Cheng, *ACS Energy Lett.* **2020**, *5*, 692.

[19] Z. Chen, Y. Ha, Y. Liu, H. Wang, H. Yang, H. Xu, Y. Li, R. Wu, *ACS Appl. Mater. Interfaces* **2018**, *10*, 7134.

[20] Q. Chen, R. Wang, M. Yu, Y. Zeng, F. Lu, X. Kuang, X. Lu, *Electrochim. Acta* **2017**, *247*, 666.

[21] W. Wang, H. Zhao, Y. Du, Y. Yang, S. Li, B. Yang, Y. Liu, L. Wang, *Chem. – A Eur. J.* **2021**, *27*, 2463.

[22] L. Yu, S. Song, B. McElhenny, F. Ding, D. Luo, Y. Yu, S. Chen, Z. Ren, *J. Mater. Chem. A* **2019**, *7*, 19728.

[23] R. Qiang, H. Wang, K. Xu, Q. Yuan, Y. Yu, L. Li, J. Wang, L. Zheng, P. C. Sherrell, J. Chen, X. Bi, *Adv. Mater. Interfaces* **2021**, *8*, 2100070.

[24] B. Yan, D. Liu, X. Feng, M. Shao, Y. Zhang, *Adv. Funct. Mater.* **2020**, *30*, 1.

[25] G. Rajeshkhanna, T. I. Singh, N. H. Kim, J. H. Lee, *ACS Appl. Mater. Interfaces* **2018**, *10*, 42453.

[26] X. Zhao, P. Pachfule, S. Li, J. R. J. Simke, J. Schmidt, A. Thomas, *Angew. Chemie - Int. Ed.* **2018**, *57*, 8921.

[27] Z. Liu, X. Zhang, H. Song, Y. Yang, Y. Zheng, B. Gao, J. Fu, P. K. Chu, K. Huo, *ChemCatChem* **2020**, *12*, 2962.

[28] D. J. Li, J. Kang, H. J. Lee, D. S. Choi, S. H. Koo, B. Han, S. O. Kim, *Adv. Energy Mater.* **2018**, *8*, 1702806.

[29] C. Cui, R. Cheng, H. Zhang, C. Zhang, Y. Ma, C. Shi, B. Fan, H. Wang, X. Wang, *Adv. Funct. Mater.* **2020**, *30*, 1.

[30] H. Jin, X. Liu, A. Vasileff, Y. Jiao, Y. Zhao, Y. Zheng, S.-Z. Qiao, *ACS Nano* **2018**, *12*, 12761.

[31] J. Song, C. Zhu, B. Z. Xu, S. Fu, M. H. Engelhard, R. Ye, D. Du, S. P. Beckman, Y. Lin, *Adv. Energy Mater.* **2017**, *7*, 1601555.

[32] A. Djire, X. Wang, C. Xiao, O. C. Nwamba, M. V. Mirkin, N. R. Neale, *Adv. Funct. Mater.* **2020**, *30*, 2001136.

[33] H. Yang, Y. Hu, D. Huang, T. Xiong, M. Li, M.-S. Balogun, Y. Tong, *Mater. Today Chem.* **2019**, *11*, 1.

[34] Z. Chen, Y. Song, J. Cai, X. Zheng, D. Han, Y. Wu, Y. Zang, S. Niu, Y. Liu, J. Zhu, X. Liu, G. Wang, *Angew. Chemie* **2018**, *130*, 5170.

[35] M. Lu, D. Chen, B. Wang, R. Li, D. Cai, H. Tu, H. Yang, Y. Zhang, W. Han, *J. Mater. Chem. A* **2021**, *9*, 1655.

[36] Y. Chen, P. Sun, W. Xing, *J. Chem. Sci.* **2019**, *131*, 1.

[37] X. Wang, Z. Li, D.-Y. Wu, G.-R. Shen, C. Zou, Y. Feng, H. Liu, C.-K. Dong, X.-W. Du, *Small* **2019**, *15*, 1804832.

[38] T. I. Singh, G. Rajeshkhanna, S. B. Singh, T. Kshetri, N. H. Kim, J. H. Lee, *ChemSusChem* **2019**, *12*, 4810.

[39] X. Zhang, C. Liang, X. Qu, Y. Ren, J. Yin, W. Wang, M. Yang, W. Huang,

X. Dong, *Adv. Mater. Interfaces* **2020**, *7*, 1.

[40] P. Wang, Z. Pu, Y. Li, L. Wu, Z. Tu, M. Jiang, Z. Kou, I. S. Amiinu, S. Mu, *ACS Appl. Mater. Interfaces* **2017**, *9*, 26001.

[41] R. Li, J. Zang, W. Li, J. Li, Q. Zou, S. Zhou, J. Su, Y. Wang, *ChemSusChem* **2020**, *13*, 3718.

[42] Y. Pan, K. Sun, S. Liu, X. Cao, K. Wu, W. C. Cheong, Z. Chen, Y. Wang, Y. Li, Y. Liu, D. Wang, Q. Peng, C. Chen, Y. Li, *J. Am. Chem. Soc.* **2018**, *140*, 2610.

[43] J. Chen, B. Ren, H. Cui, C. Wang, *Small* **2020**, *16*, 1.

[44] C. Guan, H. Wu, W. Ren, C. Yang, X. Liu, X. Ouyang, Z. Song, Y. Zhang, S. J. Pennycook, C. Cheng, J. Wang, *J. Mater. Chem. A* **2018**, *6*, 9009.

[45] H. J. Song, H. Yoon, B. Ju, G. H. Lee, D. W. Kim, *Adv. Energy Mater.* **2018**, *8*, 1802319.

[46] J. Yu, C. Lv, L. Zhao, L. Zhang, Z. Wang, Q. Liu, *Adv. Mater. Interfaces* **2018**, *5*, 1.

[47] X. H. Chen, Q. Zhang, L. L. Wu, L. Shen, H. C. Fu, J. Luo, X. L. Li, J. L. Lei, H. Q. Luo, N. B. Li, *Mater. Today Phys.* **2020**, *15*, 100268.

[48] J. Tian, Q. Liu, A. M. Asiri, X. Sun, *J. Am. Chem. Soc.* **2014**, *136*, 7587.

[49] U. N. Pan, D. R. Paudel, A. Kumar Das, T. I. Singh, N. H. Kim, J. H. Lee, *Appl. Catal. B Environ.* **2022**, *301*, 120780.

[50] K. Chang, D. T. Tran, J. Wang, N. H. Kim, J. H. Lee, *J. Mater. Chem. A* **2022**, *10*, 3102.

[51] K. Hu, M. Wu, S. Hinokuma, T. Ohto, M. Wakisaka, J. Fujita, Y. Ito, *J. Mater. Chem. A* **2019**, *7*, 2156.

[52] G. Chen, T. Y. Ma, Z. Liu, N. Li, Y. Su, K. Davey, S. Qiao, *Adv. Funct. Mater.* **2016**, *26*, 3314.

[53] C. Tang, N. Cheng, Z. Pu, W. Xing, X. Sun, *Angew. Chemie - Int. Ed.* **2015**, *54*, 9351.

[54] G. Kresse, J. Furthmüller, *Comput. Mater. Sci.* **1996**, *6*, 15.

[55] J. P. Perdew, K. Burke, M. Ernzerhof, *Phys. Rev. Lett.* **1996**, *77*, 3865.

[56] S. Grimme, J. Antony, S. Ehrlich, H. Krieg, *J. Chem. Phys.* **2010**, *132*, 154104.
